# Genotype–Phenotype Relations for the Atypical Parkinsonism Genes:MDSGene Systematic Review

**DOI:** 10.1002/mds.28517

**Published:** 2021-03-19

**Authors:** Christina Wittke, Sonja Petkovic, Valerija Dobricic, Susen Schaake, MDS-endorsed PSP Study Group, Gesine Respondek, Anne Weissbach, Harutyun Madoev, Joanne Trinh, Eva-Juliane Vollstedt, Neele Kuhnke, Katja Lohmann, Marija Dulovic Mahlow, Connie Marras, Inke R. König, Maria Stamelou, Vincenzo Bonifati, Christina M. Lill, Meike Kasten, Hans-Jürgen Huppertz, Günter Höglinger, Christine Klein

**Affiliations:** 1 Institute of Neurogenetics, University of Luebeck, Luebeck, Germany; 2 Department of Neurology, Technische Universität München, Munich, Germany; 3 German Center for Neurodegenerative Diseases (DZNE), Munich, Germany; 4 Toronto Western Hospital, University of Toronto, Toronto, Ontario, Canada; 5 Institute of Medical Biometry and Statistics, University of Luebeck, Luebeck, Germany; 6 Parkinson’s Disease and Movement Disorders Department, HYGEIA Hospital, Athens, Greece; 7 School of Medicine, European University of Cyprus, Nicosia, Cyprus; 8 Neurology Clinic, Philipps-University, Marburg, Germany; 9 Department of Clinical Genetics, Erasmus Medical Center, Rotterdam, The Netherlands; 10 Department of Psychiatry and Psychotherapy, University of Luebeck, Luebeck, Germany; 11 Swiss Epilepsy Center, Klinik Lengg AG, Zurich, Switzerland; 12 Department of Neurology, Hannover Medical School, Hannover, Germany

**Keywords:** atypical parkinsonism, genetics, Parkinson’s disease, systematic review, MDSGene, red flags

## Abstract

This Movement Disorder Society Genetic mutation database Systematic Review focuses on monogenic atypical parkinsonism with mutations in the *ATP13A2, DCTN1, DNAJC6, FBXO7, SYNJ1*, and *VPS13C* genes. We screened 673 citations and extracted genotypic and phenotypic data for 140 patients (73 families) from 77 publications. In an exploratory fashion, we applied an automated classification procedure via an ensemble of bootstrap-aggregated (“bagged”) decision trees to distinguish these 6 forms of monogenic atypical parkinsonism and found a high accuracy of 86.5% (95% CI, 86.3%–86.7%) based on the following 10 clinical variables: age at onset, spasticity and pyramidal signs, hypoventilation, decreased body weight, minimyoclonus, vertical gaze palsy, autonomic symptoms, other nonmotor symptoms, levodopa response quantification, and cognitive decline. Comparing monogenic atypical with monogenic typical parkinsonism using 2063 data sets from Movement Disorder Society Genetic mutation database on patients with *SNCA, LRRK2, VPS35, Parkin, PINK1*, and *DJ-1* mutations, the age at onset was earlier in monogenic atypical parkinsonism (24 vs 40 years; *P* = 1.2647 × 10^−12^) and levodopa response less favorable than in patients with monogenic typical presentations (49% vs 93%). In addition, we compared monogenic to nonmonogenic atypical parkinsonism using data from 362 patients with progressive supranuclear gaze palsy, corticobasal degeneration, multiple system atrophy, or frontotemporal lobar degeneration. Although these conditions share many clinical features with the monogenic atypical forms, they can typically be distinguished based on their later median age at onset (64 years; IQR, 57–70 years). In conclusion, age at onset, presence of specific signs, and degree of levodopa response inform differential diagnostic considerations and genetic testing indications in atypical forms of parkinsonism.

This article on monogenic atypical parkinsonism from the MDSGene Systematic Review series complements previous reviews on genetic parkinsonism with clinically overall typical Parkinson’s disease (PD) and dominant (*SNCA, LRRK2, VPS35*)^1^ or recessive inheritance (*Parkin, PINK1, DJ1*).^2^ The Movement Disorder Society Genetic mutation database (MDSGene; www.mdsgene.org) provides a comprehensive online resource linking reported genetic mutations with movement disorder phenotypes and other demographic and clinical information.^3,4^ It lists 6 forms of monogenic parkinsonism with prominent additional features atypical for PD (PARK-*ATP13A2*, PARK-*DNAJC6*, PARK-*FBXO7*, PARK-*SYNJ1*, PARK-*VPS13C*, and PARK-*DCTN1*), such as spasticity, prominent cognitive impairment, and other nonmotor features. It is important to note that several additional genes have been linked to atypical parkinsonism including those where (1) a prominent other movement disorder is typically present (e.g., *PLA2G6*, which will be covered in conjunction with other forms of dystonia-parkinsonism), (2) developmental delay is the presenting or an early feature (e.g., *RAB30B*), or (3) associations awaiting independent confirmation. Another limitation of the selection of genes for this review is that the field of PD genetics is in constant flux, with candidates being confirmed, refuted, or newly identified in rapid succession.

Although mutations in *ATP13A2, SYNJ1, DNAJC6, FBXO7*, and *VPS13C* act in a recessive manner, dominantly inherited mutations in *DCTN1* cause Perry syndrome. While monogenic parkinsonism with clinically typical presentations has been extensively covered in numerous review articles over the past 2 decades, there is an overall scarcity of comprehensive reviews on the atypical forms with the exception of a previously published book chapter on clinicogenetic relationships of atypical parkinsonism.^5^ The present review largely extends and updates this chapter and contains several additional features.

With special emphasis on clinical red flags and genetic testing considerations, we compared demographic, clinical, and genetic features of the different monogenic atypical parkinsonism forms among each other employing an automated classification procedure based on machine learning in an exploratory fashion. With this, we aimed to investigate in an objective and quantitative way whether it is in principle possible to differentiate the 6 monogenic forms of parkinsonism on the basis of their clinical signs and symptoms and to assess whether a few symptoms may be sufficient for differentiation (and may potentially serve as red flags in clinical practice). Second, we systematically contrasted phenotypic features of monogenic atypical parkinsonism with those of the overall clinically typical monogenic forms as well as with, third, nonmonogenic atypical parkinsonism, including multiple system atrophy (MSA), progressive supranuclear gaze palsy (PSP), corticobasal degeneration (CBD), and frontotemporal lobar degeneration (FTLD).

## Methods

The systematic literature search and data extraction procedure followed the MDSGene protocol,^1,2^ and protocol and data have been posted online at MDSGene (www.mdsgene.org).

### Literature Search and Eligibility Criteria

A systematic literature search for publications on *ATP13A2, DNAJC6, SYNJ1, FBXO7, VPS13C*, and *DCTN1* mutation carriers was performed using the PubMed database (http://www.pubmed.gov) applying gene-specific search terms (Table S1). All peer-reviewed and original articles in English, published until February 12, 2020, were assessed for eligibility using the title, abstract, or full text, as necessary. Every publication that described at least 1 affected carrier of biallelic *ATP13A2, DNAJC6, SYNJ1, FBXO7*, or *VPS13C* or heterozygous *DCTN1* mutations was included in the present study. Furthermore, all articles were screened for references referring to additional articles on affected mutation carriers. These articles were also included in the MDSGene database. For an overview of the literature search as well as the filtering procedure, see Figure S1. A list of all eligible articles can be found in Supplementary Methods 1.

### Inclusion and Exclusion Criteria for Genetic Variants and Study Participants

Variants with a minor allele frequency (MAF) ≥ 1% based on ethnicity, with the maximum MAF in the ExAC Browser (http://exac.broadinstitute.org), dbSNP (http://www.ncbi.nlm.nih.gov/snp/), and/or unaffected control individuals screened for the variant of interest in the respective publication, were excluded from this investigation.

Clinical data were extracted for all mutation carriers with parkinsonism with at least 1 element of clinical information recorded as previously described.^1^ To systematically distinguish between PD and atypical parkinsonism, we applied the absolute exclusion criteria for PD, resulting in atypical parkinsonism from the MDS clinical diagnostic criteria for PD^6^ whenever possible.

### Data Collection Process

Demographic, genetic, and clinical data were extracted from eligible articles according to a standard protocol.^1^ The list of extracted variables is available in Table S2. In case a patient had been reported more than once in subsequent publications, we combined all information. The genetic data comprised physical location, genomic, cDNA, and protein nomenclature, and zygosity (homozygous, compound-heterozygous, and heterozygous). To avoid and to be able to complete missing nomenclature whenever possible, we used the information available from the publication, Ensembl (http://www.ensembl.org), and MutationTaster databases (http://www.mutationtaster.org). All mutations were mapped to GRCh37/hg19, and nomenclatures were based on the transcripts *ATP13A2* ENST00000326735, *DNAJC6* ENST00000371069, *SYNJ1* ENST00000382499, *FBX O7* ENST00000266087, *VPS13C* ENST00000261517, and *DCTN1* ENST00000361874 (http://www.ensembl.org), respectively. Variants were classified as “definitely pathogenic,” “probably pathogenic,” “possibly pathogenic,” or “benign” as previously described (http://www.mdsgene.org/methods^1,2,7,8^ and Supplementary Methods 2). This system has been adapted from the recommendations from the American College of Medical Genetics to better reflect information on segregation of pathogenic variants and to improve the distinction of “‘variants of unknown significance.” Variants scored as benign were excluded from further analyses (Table S4).

### Data Collection on Nonmonogenic Atypical Parkinsonism

Data were collected after obtaining written informed consent of participants and/or guardians and formal ethical approval by the relevant research ethics committee of the Technical University of Munich and the other participating centers (Ludwig-Maximilians-University, Munich, Germany; University Hospital, Bordeaux, France; King’s College, London, UK; Lund University, Sweden; Erasmus Medical Center, Rotterdam, The Netherlands; Hospital Clinic-IDIBAPS, Barcelona, Spain; University of Saskatchewan, Saskatoon, Canada; Johns Hopkins University, Baltimore, Maryland, USA; University of Pennsylvania, Philadelphia, PA, USA). Only cases with a neuropathological diagnosis of PSP,^9-11^ CBD,^10,12^ MSA,^13^ and 4R-tau-negative FTLD,^14^ according to published criteria, were included. The database of nonmonogenic atypical parkinsonism comprised 362 patients. Clinical features used for comparison included AAO, age at death, disease duration, sex, cognitive dysfunction, hallucinations, presence of supranuclear gaze palsy, abnormal saccades or pursuit, dysarthria, autonomic dysfunction, presence of falls, and levodopa responsiveness.

### Statistical Analyses

To compare clinical and demographic data between groups of individuals, we used SPSS 25.0.0.1 (IBM, Armonk, NY) for statistical analyses. Data were not normally distributed; thus, we applied the Mann–Whitney *U* test to compare between groups. To account for multiple testing, we used a Bonferroni adjusted α level of 0.0125 (0.05/4).

### Exploratory Automated Classification of Monogenic Atypical Parkinsonism

We used an ensemble of bootstrap-aggregated (“bagged”) decision trees as implemented in the so-called TreeBagger method of MATLAB (R2020a; The Math Works, Inc., Natick, MA). The 4 analyses based on this method (i.e., regarding overall classification performance, the relative importance of the clinical input variables, effect of a reduced set of input variables, and effect of prior knowledge of the diagnoses frequencies) are described in detail in Supplementary Methods 3.

## Results

**FIG. 1.**
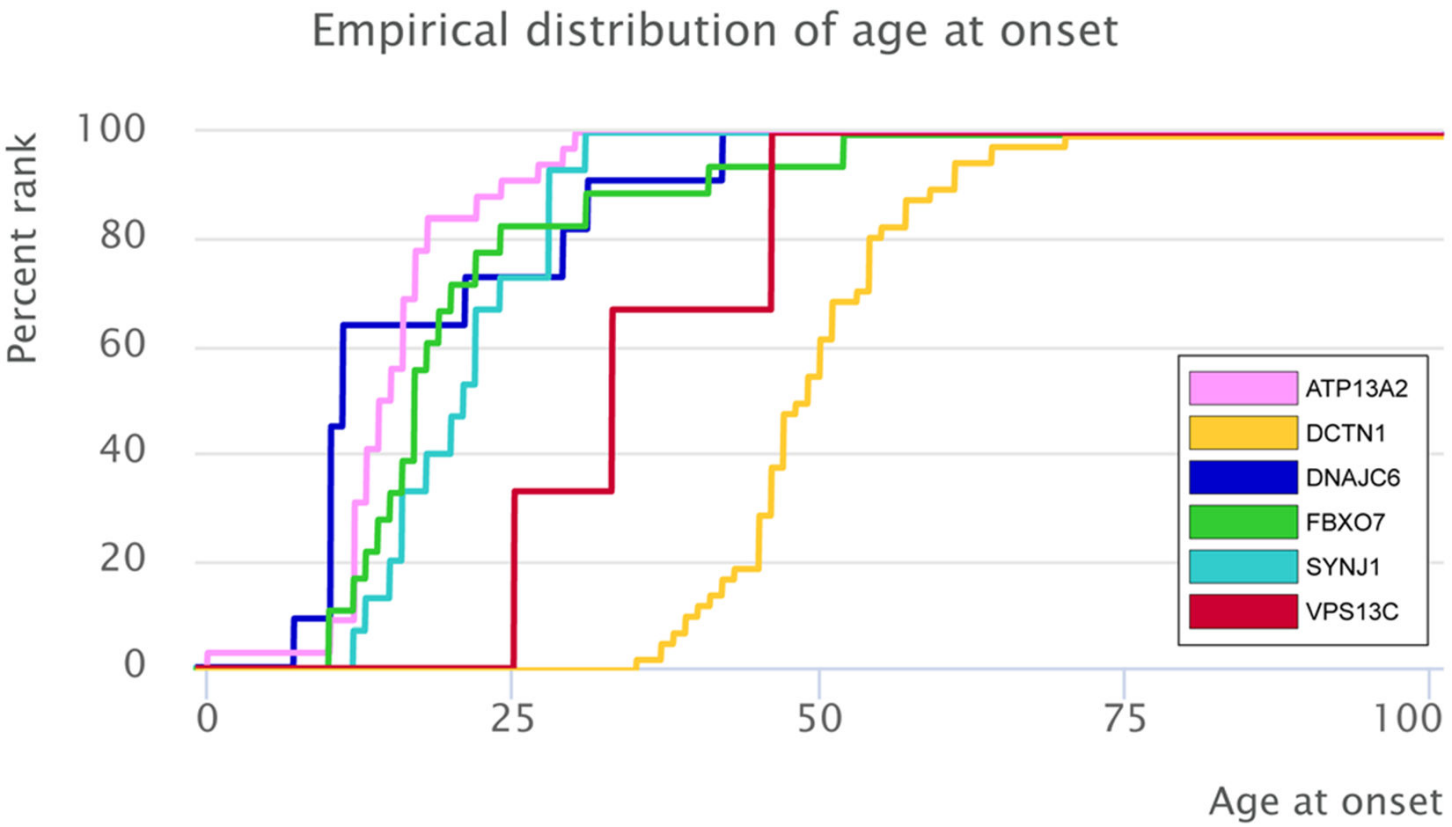
Empirical distribution of the age at onset for *ATP13A2, DNAJC6, FBXO7, SYNJ1, VPS13C*, and *DCTN1*.

Our PubMed literature search yielded a total of 673 citations, among which 77 studies were eligible for data extraction (Fig. S1, Supplementary Methods 1) and included information on 140 patients from 73 families. The median AAO across the 127 patients with this information available was 24 years (interquartile range [IQR], 15–47 years). AAO differed between carriers of mutations in the 5 recessive genes and carriers of mutations in the dominantly inherited *DCTN1* (*P* = 2.7 × 10^−19^; Table S5 and Fig. S2). The youngest median AAO was reported for *DNAJC6* mutation carriers (11 years; IQR, 10–29 years), the highest for *DCTN1* mutation carriers (49 years; IQR, 46–54 years; Fig. 1, Table S5). The percentage of women across all patients was 42.5% (n = 55; 5% missing data). Patients originated from 29 different countries (Fig. S3A-F). Genetic data for 57 pathogenic variants were extracted across the 6 analyzed genes. Most of the variants were classified as probably pathogenic (n = 40, 70.1%), 13 as definitely pathogenic (22.8%), and 4 variants as possibly pathogenic (7.1%); see Table S6. Missense mutations represented the most frequent mutation type in genes linked to atypical parkinsonism (n = 29, 50.9%). The remaining variants comprised 12 frameshift mutations (21.1%), 9 nonsense mutations (15.8%), 5 splice-site mutations (8.8%), 1 silent mutation (1.8%), and 1 structural variant (1.8%); see Figure S4A-F. Overall, 36 of 47 index patients carrying variants in the recessive genes *ATP13A2, SYNJ1, DNAJC6, FBXO7*, or *VPS13C* (76.6%) were homozygous, and 11 were compound-heterozygous. For the *DCTN1* gene, all 26 index patients carried a heterozygous mutation. The following paragraphs list phenotypic and mutational details by gene.

### ATP13A2

**FIG. 2.**
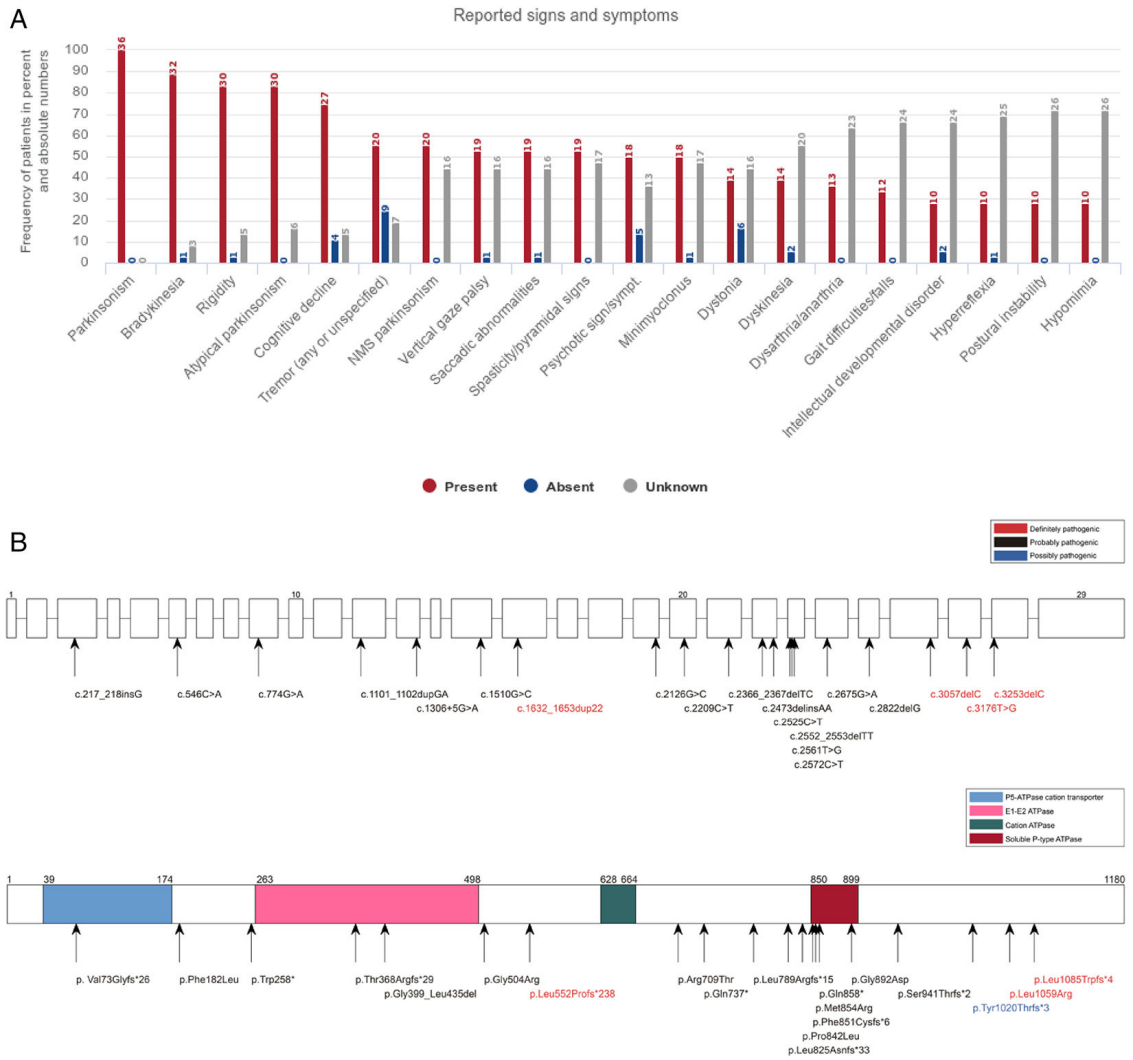
Clinical and genetic findings in patients with *ATP13A2* mutations according to MDSGene. (**A**) Reported signs and symptoms are sorted according to their frequency in *ATP13A2* mutation carriers (limited to signs and symptoms with a frequency of at least 25%). (**B**) Schematic representation of the *ATP13A2* gene (upper scheme) and protein (lower scheme) with mutations listed in MDSGene. Splice-site mutations are not depicted in the protein because of unpredictable effect.

Thirty-six PARK-*ATP13A2* patients originated from 19 families are spread across the globe (missing data, 3 families; Fig. S3A). The median AAO was 14 years (IQR, 12–17 years), with 77.8% showing a juvenile AAO (AAO < 20 years); see Table S5 and Figure S2A. Frequencies of signs and symptoms are summarized in Figure 2A. Overall, atypical parkinsonism was reported in 83.3% of the patients (n = 30). Apart from cardinal signs of parkinsonism, cognitive decline was the most common clinical feature present, in 75.0% of cases with available information (n = 27). Other common reported atypical features comprised saccadic abnormalities, vertical gaze palsy, spasticity/pyramidal signs (all n = 19, 52.8%), minimyoclonus (n = 18, 50.0%), and psychotic signs and symptoms, that is, psychosis (n = 18, 50.0%). These and additional signs and symptoms are reported in Figure 2A; presenting signs are listed in Figure S2A. Levodopa therapy was implemented in 86.7% of patients (n = 26, missing data: 6 patients), resulting in a good (n = 9, 34.7%), moderate (n = 8, 30.8%), or poor (n = 6, 23.1%) treatment response (Fig. S5A), with dyskinesia the most commonly reported side effect.

Data extraction revealed 19 different variants for the *ATP13A2* gene. Seventy-five percent of all index patients (27 patients) carried a homozygous mutation, and 25.0% (9 patients) were compound-heterozygous (although specific information on zygosity was rarely provided). In addition, 28 unaffected family members from 10 families were reported to carry the respective causal mutation in the heterozygous state. The most common mutation type was a frameshift mutation (9 mutations, 45.0%), followed by missense (7 mutations, 35.0%), nonsense (3 mutations, 15.0%), and splice-site (1 mutation, 5.0%) mutations (Fig. S4A and Table S6). A detailed overview of the location of mutations alongside their pathogenicity status in the *ATP13A2* gene and ATP13A2 protein is depicted in Figure 2B.

### DNAJC6

The 11 *DNAJC6* mutation carriers originated from 5 families (Fig. S3B). The median AAO of all *DNAJC6* mutation carriers was 11 years (IQR, 10–29 years), with the majority (63.4%) having a juvenile AAO (Table S5 and Fig. S2B). Detailed clinical information is summarized in Figure S6A. In addition to the cardinal parkinsonism signs, spasticity/pyramidal signs and dysarthria/anarthria were the most common clinical features. Two patients were described to suffer from “typical PD.”^15^ Nine of the patients (81.8%) received levodopa therapy, with 7 having a good/excellent response (77.8%); see Figure S5B. However, 75.0% of the patients (n = 6 of 8) subsequently developed dyskinesia (n = 3) and psychiatric side effects (n = 4).

A total of 5 different homozygous pathogenic variants were reported in the *DNAJC6* gene. The largest described *DNAJC6* family comprises 4 affected and 9 unaffected members who are all carriers of the nonsense mutation p. Gln791* in the homozygous and heterozygous state, respectively (Fig. S4B, Table S6, and Fig. S6B).

### FBXO7

The 26 *FBXO7* mutation carriers originated from 10 families (Fig. S3C). The median AAO of all *FBXO7* mutation carriers was 17 years (IQR, 14–21 years), with the majority (n = 17, 70.6%) having a juvenile AAO (-Table S5 and Fig. S2C). Detailed clinical data are summarized in Figure S7A. The cardinal parkinsonism signs were common, as were spasticity/pyramidal signs in 73.1% of the cases. Overall, atypical parkinsonism symptoms were reported in 92.3% of patients (n = 24; Fig. S7A). Initial signs are listed in Figure S2C. Eighteen patients (69.2%) received levodopa therapy, with 54.4% of the patients (n = 6) with available information responding well, 27.3% (n = 3) moderately responding, and 18.2% (n = 2) showing a minimal response (Fig. S5C).

Seven different pathogenic variants were reported in the *FBXO7* gene, all in the homozygous or compound-heterozygous state. In addition, 19 family members from 5 families were reported to carry the respective causal genotype in the heterozygous state, none of whom showed any clinical signs or symptoms. The nonsense mutation p.Arg498* was the most frequent mutation and found in 6 index patients, all in the homozygous state (Fig. S4C, Table S6, and Fig. S7B).

### SYNJ1

For *PARK-SYNJ1*, the search yielded a total of 17 patients who originated from 9 families (Fig. S3D). The median AAO of all patients was 22 years (IQR, 16–28 years) with the majority (n = 9, 52.9%) showing an early AAO, 35.3% with a juvenile AAO (n = 6), and 11.7% (n = 2) with a late AAO (Table S5 and Fig. S2D).

Detailed clinical data are summarized in Figure S8A. In addition to the cardinal signs, the most commonly reported features were dystonia (n = 11, 64.7%), gait difficulties (n = 8, 47.1%), cognitive decline, postural instability, hypomimia (all n = 7, 41.2%), and dysarthria/anarthria (n = 6, 35.3%); see Figure S8A. Levodopa therapy was administered to 88.2% of patients (n = 15) and beneficial in 52.9% (n = 9); see Figure S5D. Two patients were reported to benefit from clonazepam combined with pramipexole.^16^

In the 17 *PARK-SYNJ1* patients, 9 different mutations were detected in the homozygous or compound-heterozygous state (Fig. S4D and Table S6). The most frequent mutation, c.773G > A, was found in 4 index patients, 3 in the homozygous and 1 in the compound-heterozygous states (Fig. S4D and Fig. S8B).

### VPS13C

Only 4 PARK-*VPS13C* patients from 4 families (Fig. S3E) are currently reported precluding the calculation of percentages. AAO was available for 2 of the patients and reported as 25 and 33 years (Fig. S2E). In addition to the cardinal parkinsonism signs, the most frequent atypical parkinsonism signs and symptoms were gait difficulties/falls, hyperreflexia, swallowing disorder, and cognitive decline found in 3 patients (Figs. S9A and S2E). For 3 patients with available information, levodopa response was indicated as “moderate” (Fig. S5E).

All 6 *VPS13C* mutations were private mutations (Fig. S4E) and homozygous or compound-heterozygous. Eleven family members from 4 families were reported to carry the causal genotype in the heterozygous state and to not show any clinical signs or symptoms. Two of the 6 *VPS13C* mutations were frameshift mutations (Fig. S4E). All 6 sequence variants were classified as “probably pathogenic” (Table S6 and Fig. S9B).

### DCTN1 (*Perry Syndrome*)

**FIG. 3.**
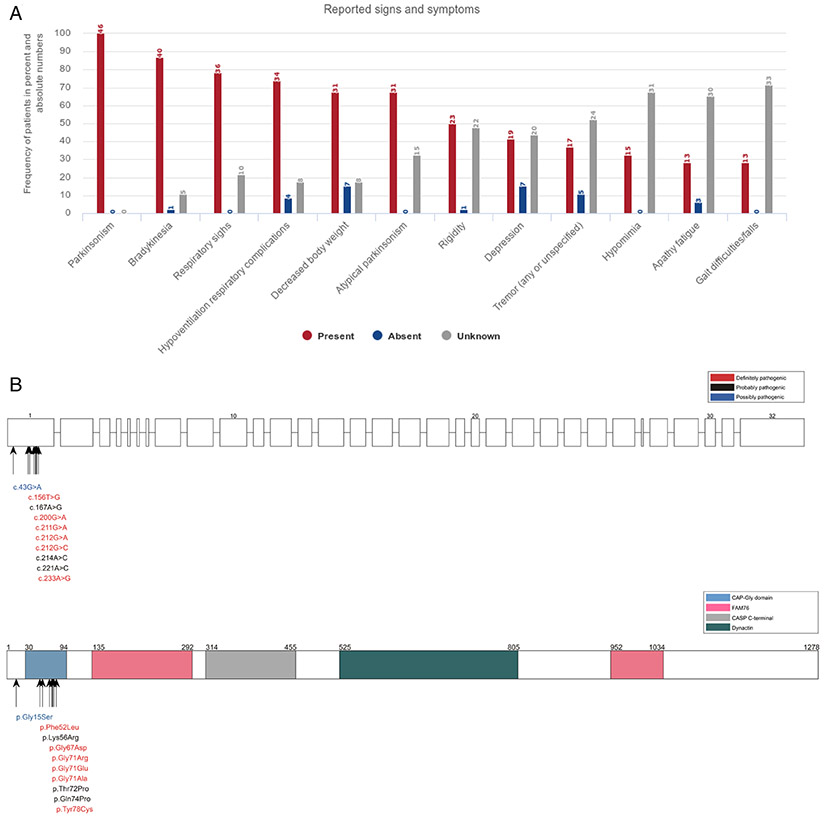
(**A**) Reported signs and symptoms in PD patients with *DCTN1* mutations. Signs and symptoms are listed according to their frequency (limited to signs and symptoms with a frequency of at least 25%). (**B**) Schematic representation of the *DCTN1* gene (upper scheme) and protein (lower scheme) and mutations listed in MDSGene.

The 46 PARK-*DCTN1* patients with *DCTN1* mutations linked to Perry syndrome originated from 26 families (Fig. S3F). The median AAO of all patients was 49 years (IQR, 46–54 years), with the majority (89.1%) showing late onset, with AAO > 40 years (Table S5 and Fig. S2F). Detailed clinical data are summarized in Figure 3A. Apart from the cardinal parkinsonism signs, hypoventilation/respiratory complications in 73.9% (n = 34), weight loss (indicated as decreased body weight in the figure) in 67.4% (n = 31), depression in 41.3% (n = 19), hypomimia in 32.6% (n = 15), apathy/fatigue, and gait difficulties/falls in 28.3% (n = 13) were the most common clinical features. For the 30 patients with reported initial signs and symptoms (missing data, 34.8%; n = 16), 33.3% (n = 10) presented with depression and apathy/fatigue and 26.6% (n = 9) with bradykinesia (n = 8); see Figure S2F. Twenty-six patients (56.5%) received levodopa therapy including 92.3% of responders (n = 24); see Figure S5F. Side effects of levodopa therapy were described in 16.7% of patients (n = 5), most frequently dyskinesia (n = 4).

A total of 10 different, heterozygous pathogenic missense variants were reported in the *DCTN1* gene (Fig. S4F and Table S6). The amino acid residue 71 (glycine) is repeatedly affected by mutations with 3 different substitutions (arginine, glutamic acid, alanine) found in 57.7% of affected families (n = 15); see Figures S4F and 3B.

#### Classification of Monogenic Atypical Parkinsonism

Four different analyses were performed to classify monogenic atypical parkinsonism.

**FIG. 4.**
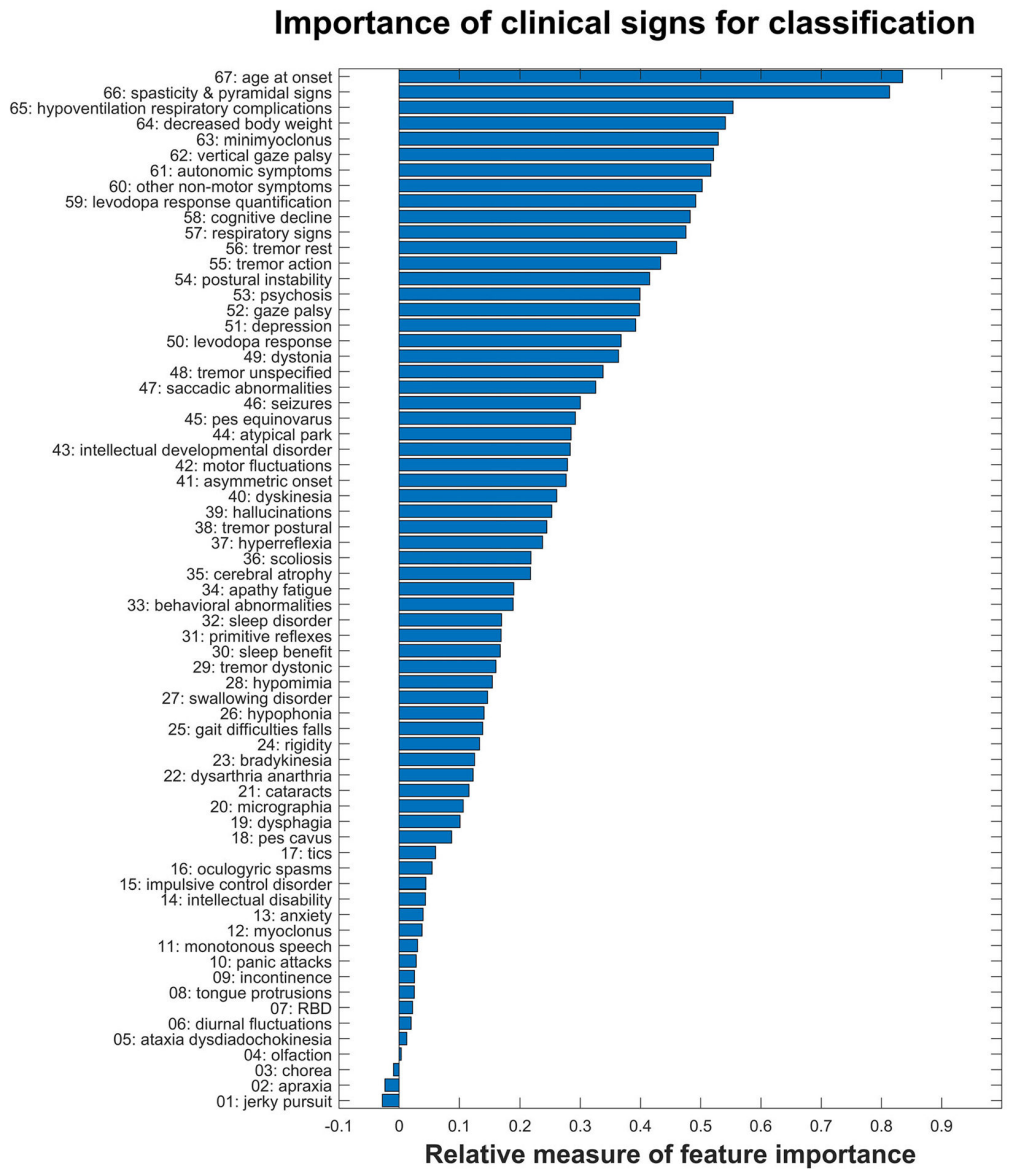
Relative importance of the 67 clinical variables for classification performance to distinguish the 6 forms of monogenic atypical parkinsonism. The first 10 clinical features contribute 86.5% of the classification accuracy.

In analysis 1, our classifier’s ability to distinguish monogenic atypical parkinsonism forms from each other according to their clinical characteristics and its performance in new and unseen data were estimated by 2 approaches, that is from “out-of-bag” (OOB) predictions and by leave-one-out cross-validation (LOOCV). Both approaches yielded almost identical results, differing by less than 1% in all analyses. For analysis 1, the OOB approach resulted in a total accuracy (TA) of 91.7% (95%CI, 90.%8–91.4%) and a balanced accuracy (BA) of 81.3% (95%CI, 81.0%–81.6%), whereas TA and BA for LOOCV were 91.0% (95%CI, 90.8%–91.2%) and 81.2% (95%CI, 81.1%–81.4%), respectively. For brevity, the presentation of results (for individual diagnosis groups, corresponding figures, and subsequent analyses 2–4) will be limited to those of the LOOCV approach. Figure S10 shows the confusion matrix for all 6 classes/diagnosis groups, as averaged across 20 repetitions, together with results for the true-positive rate (TPR), false-negative rate, positive predictive value, and false discovery rate. The smallest diagnosis group (i.e., *VPS13C*), consisting of only 4 members, had the lowest sensitivity (50%). For all other groups, the sensitivities (TPR) ranged from 73% (for *DNAJC6*) to 100% (for *DCTN1*). When assessing the importance of the different clinical signs for classification, the following 10 clinical variables showed the highest importance (in descending order of contribution): age at onset and spasticity and pyramidal signs, followed by hypoventilation and respiratory complications, decreased body weight, minimyoclonus, vertical gaze palsy, autonomic symptoms, other nonmotor symptoms, levodopa response quantification, and cognitive decline. Figure 4 presents the corresponding results for all 67 clinical variables included. Additional, exploratory analyses are included in Supplementary Methods 4.

#### Comparison of Clinical-Genetic Information Across Different Forms of Monogenic Clinically Typical and Atypical Parkinsonism

The MDSGene database includes clinical information on 930 patients with dominant, clinically typical (*SNCA, LRRK2, VPS35*)^1^ PD and 1127 patients with recessive, clinically typical (*Parkin, PINK1, DJ1*)^2^ monogenic PD. Patients with clinically typical, recessive monogenic PD had an earlier AAO than those with dominant forms (*P* = 3.5 × 10^−211^); likewise, patients with recessive atypical PD had an earlier AAO than those with a dominant form (*P* = 2.7 × 10^−19^). Patients with dominant, clinically typical monogenic PD presented with the highest median AAO (55 years), followed by patients with dominant, clinically atypical parkinsonism (49 years), recessive, clinically typical monogenic PD (31 years), and recessive, clinically atypical parkinsonism (16 years); see Table S5. In recessively inherited, clinically typical PD, the most frequently observed initial signs were tremor (65%), bradykinesia (25%), and dystonia (15%),^2^ which was similar to the atypical recessive forms of parkinsonism (bradykinesia in 58%, tremor in 33%, and rigidity in 22% of the patients; Table S7A). A good or even excellent response to levodopa therapy was observed in ~93% of patients reported with dominant and recessive clinically typical PD compared with 54%, and 36% in recessive and dominant clinically atypical parkinsonism, respectively (Table S7B).

#### Comparison of Monogenic Atypical Parkinsonism With Nonmonogenic Atypical Parkinsonism

To identify monogenic mimics of nonmonogenic atypical parkinsonism, information on 362 patients was used including clinical data on PSP (202 cases), CBD (55 cases), MSA (51 cases), and FTLD (54 cases). Demographic and clinical features are summarized in Table 1. The nonmonogenic atypical parkinsonism patients had a median AAO of 64 years (IQR, 57–70 years; Table S5). Cognitive decline, dysarthria/anarthria, and falls were the most frequent signs among all patients with nonmonogenic atypical parkinsonism. The largest clinical overlap was observed for PARK-*ATP13A2* and PSP, which frequently presented with cognitive decline (PSP, 63.9%; PARK-*ATP13A2*, 75%), vertical gaze palsy (PSP, 68.8%; PARK-*ATP13A2*, 52.8%), abnormal saccades (PSP, 63.9%; PARK-*ATP13A2*, 52.8%), dysarthria/anarthria (PSP, 68.8%; PARK-*ATP13A2*, 36.2%), and gait difficulty/falls (PSP, 76.2%; PARK-*ATP13A2*, 33.3%). PARK-*DNAJC6* and PARK-*DCTN1* mimic features of MSA including spasticity and respiratory problems, respectively.

## Discussion

The present MDSGene review on monogenic atypical parkinsonism contains data on a total of 140 patients with mutations in 6 different genes (*ATP13A2, DCTN1, DNAJC6, FBXO7, SYNJ1,and VPS13C*). We recapitulate many of the previously described findings, including early onset of a clinically atypical form of parkinsonism with a rather poor levodopa treatment response compared with monogenic typical PD. One of the most striking findings in our 2 previous reviews on monogenic typical PD was the proportion of missing phenotypic data. Our present data set mostly consists of case reports and family studies, which are less prone to data missingness compared with mutational screens or mixed types of studies (Fig. S11). Regarding the location of mutations, it was observed that variants in the 5 recessive genes were spread across the entire coding region of the gene, whereas variants in *DCTN1* were reported exclusively in the first exon. More than two-thirds of the variants were scored as probably pathogenic and the remainder as definitely or possibly pathogenic. Variants c.773G > A in *DCTN1* and c.1492C > T in *FBXO7* may be considered mutational hot spots because they were reported in 30% and 50% of the index patients, respectively.

Although our classification analysis was exploratory in nature and lacking validation because of current unavailability of an independent data set, we demonstrated that it is principally possible to differentiate these 6 monogenic forms of monogenic atypical PD based on their clinical signs and symptoms. However, it remains to be seen if and how this may be generalizable to a larger sample with a greater number of different forms of PD. We were able to identify 10 clinical variables that contribute most significantly to the differentiation and apparently more so than the remainder of the other 57 signs and symptoms collected for the present review. When limiting the number of input parameters to these 10 most important clinical variables, total accuracy reached >86%. For this, a large number of decision trees combined in a machine-learning approach were required to capture the multitude of input variables and to model the associated probabilities for the 6 conditions in question. In view of the partially missing data for certain features across all 6 forms of monogenic atypical parkinsonism, the accuracy of the classification achieved in this way is remarkable and probably a consequence of the defining feature of Random Forests that each decision tree is trained with only a subset of all available clinical variables. Not surprisingly, AAO and pyramidal signs stood out as the most important “red flags,” followed by a series of more specific clinical signs. As expected, the classification performance worsened with decreasing case numbers, especially for conditions with few reported cases, and it should be noted that the estimates of classification accuracies in this study (i.e., “out-of-bag” predictions and cross-validation) carry the risk of a positive bias.^17^ As soon as available, a validation on an independent data set is warranted.

Early-onset atypical parkinsonism may also be the predominant phenotype in recessively inherited dopa-responsive dystonias in other pallidopyramidal disorders including neurodegeneration with brain iron accumulation in young-onset Huntington’s disease or Niemann–Pick type C disease.^18,19^ A detailed description of these additional differential diagnoses is beyond the scope of the present review. In addition to genetic testing, they are typically revealed through clinical and imaging clues.^18-20^ The later age at onset and the frontal features caused by *DCTN1* mutations are shared by patients with other mutations in genes implicated in frontotemporal dementia/parkinsonism, such as *GRN* and *C9ORF72*.^21^ In addition, weight loss and hypoventilation present in patients with *DCTN1* mutations are typically also found in patients with stiffness or atypical parkinsonism because of DPPX and IGLON5 antibodies, respectively.^22-24^

**FIG. 5.**
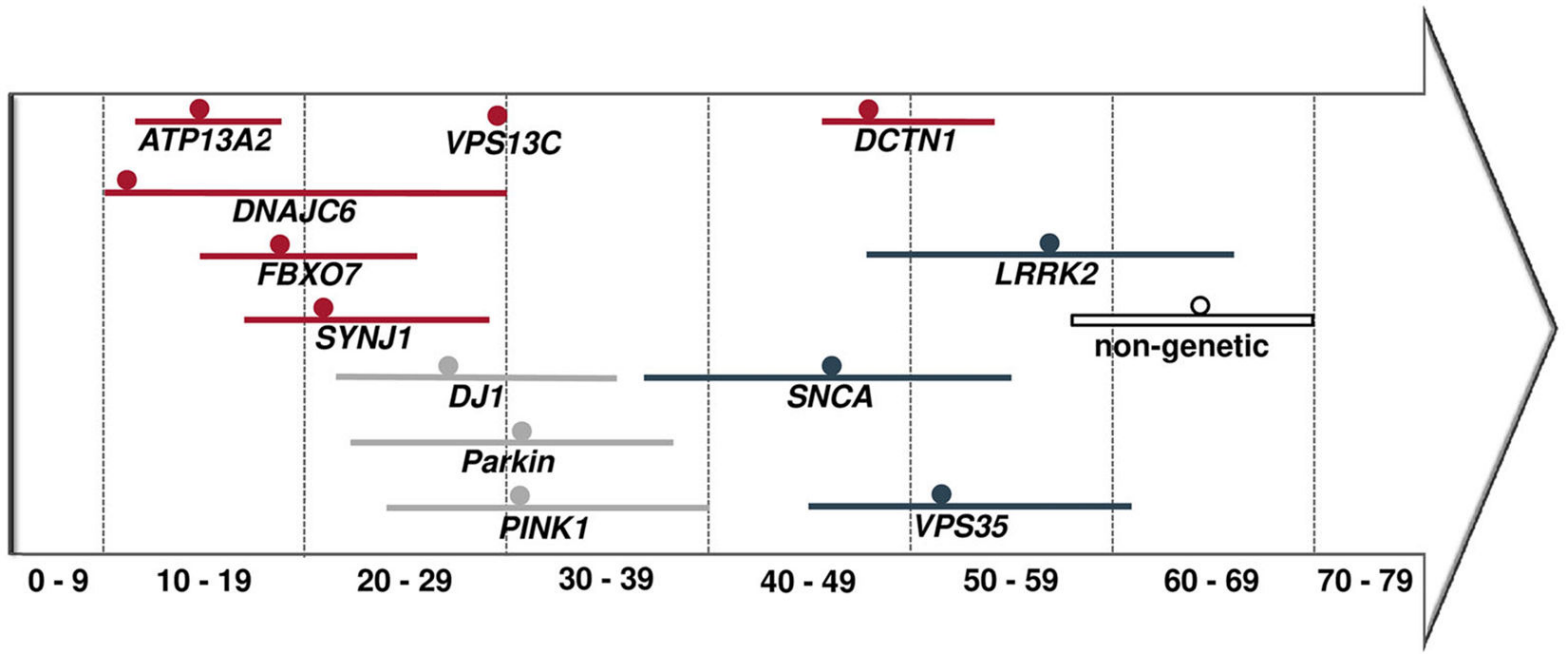
Comparison of the ranges and medians of AAOs in years. Colored lines, ranges (no range for *VPS13C* available); dots, median; red, AAO for monogenic atypical parkinsonism; gray, monogenic recessive PD; blue, monogenic dominant PD; boxed, nonmonogenic atypical parkinsonism.

When comparing clinically typical dominant (PARK-*SNCA*, PARK-*LRRK2*, PARK-*VPS35*)^1^ and recessive (PARK-*Parkin*, PARK-*PINK1*, and PARK-*DJ1*)^2^ forms of monogenic PD with the atypical forms investigated here, the AAO was lowest overall in the recessive atypical group, with a median AAO of 16 years, followed by the recessive typical forms (31 years), the dominant atypical (49 years) and the dominant typical (55 years) forms and is highest in patients with nonmonogenic atypical parkinsonism (64 years). Moreover, combining all available data on AAO for both monogenic typical and atypical forms revealed that for all recessive forms, median AAO was before 40 years and for almost all dominant forms, it was after 40 years (Fig. 5). In addition to the overall absence of atypical features in the above-mentioned forms of clinically typical monogenic parkinsonism, levodopa response is more favorable (93%) than in patients with monogenic atypical presentations (49.0%).

Monogenic and nonmonogenic atypical parkinsonism present with several overlapping clinical features. However, the median AAO was overall significantly higher in nonmonogenic atypical parkinsonism. In fact, in our large data set, an AAO ≤ 40 and ≤ 50 years precludes a clinical diagnosis of PSP^25^ and CBD,^26^ respectively. Regarding specific forms of monogenic atypical parkinsonism, *ATP13A2* mutation carriers present with a syndrome that shares several features with PSP, that is, parkinsonism and cognitive decline, saccadic abnormalities, vertical gaze palsy, and falls as in classical PSP,^25,27^ as well as myoclonus and dystonia as in classical CBD.^26^ Overlapping signs of the clinical syndrome of *DNAJC6* mutations and MSA include parkinsonism, pyramidal signs, dysarthria/anarthria, and dystonia (respiratory signs are a red flag for MSA).^28^ However, an AAO of <30 years is not compatible with a clinical diagnosis of MSA.^28^ *FBXO7* mutations may cause a syndrome similar to primary progressive aphasia, particularly when coexisting with cognitive decline and behavioral abnormalities. This syndrome may be observed in patients with underlying PSP or CBD pathology as well as in other forms of FTLD.^29^ Psychosis, however, is not typically observed in PSP and CBD.^30^ Other distinctive clinical clues for *FBXO7* mutations may be predominant spasticity and *pes equinovarus*. The clinical spectrum of *SYNJ1* mutations may be reminiscent of that of PSP, CBD, and MSA. Overlapping features include dystonia, postural instability and falls, cognitive decline, hypomimia, and dysarthria. However, seizures and earlier age at onset are distinctive features of *SYNJ1* mutations. PARK-VPS13C shares features with PSP with predominant postural instability, dysphagia, and cognitive decline. *DCTN1* mutations may present with features of PSP and CBD including atypical parkinsonism, cognitive decline, saccadic abnormalities, vertical gaze palsy, falls, myoclonus, and dystonia. Best clinical discriminators are family history, hallucinations and psychosis, as well as hypoventilation/respiratory complications.

Taken together, although age at onset, the presence of specific clinical signs, and degree of levodopa response inform differential diagnostic considerations and genetic testing indications in atypical forms of parkinsonism, additional investigations are warranted including but not limited to (1) postmortem studies in patients with monogenic atypical parkinsonism and comparison of the findings with those from monogenic typical parkinsonism and nonmonogenic atypical parkinsonism, (2) exploring potential pathophysiological links between forms of atypical parkinsonism with a high(er) level of shared phenotypic features, (3) prioritizing forms of (monogenic and nonmonogenic) atypical parkinsonism with phenotypic overlap for the development and testing of targeted therapies.

## Supplementary Material

Supplementary Material

## Figures and Tables

**TABLE 1.** Clinical information on nonmonogenic atypical parkinsonism patients

	PSP	CBD	FTLD	MSA	Total
Number of patients	202	55	54	51	362
Age at onset (years), median (range)	66.0 (61–72)	61.0 (58–71)	55.5 (30–72)	59.0 (49–58)	64.0 (30–72)
Cognitive decline	138 (68.3%)	42 (76.4%)	49 (90.7%)	15 (29.4%)	244 (67.4%)
Hallucinations	6 (3.0%)	1 (1.8%)	0 (0%)	7 (13.7%)	14 (3.9%)
Vertical gaze palsy	139 (68.8%)	9 (16.4%)	1 (1.9%)	5 (9.8%)	154 (42.5%)
Abnormal saccades	129 (63.9%)	18 (32.7%)	4 (7.4%)	14 (27.5%)	165 (45.6%)
Dysarthria/anarthria	139 (68.8%)	21 (38.2%)	14 (25.9%)	47 (92.2%)	221 (61.0%)
Autonomic dysfunction	65 (32.2%)	11 (20.0%)	4 (7.4%)	44 (86.3%)	124 (34.3%)
Levodopa response	33 (16.3%)	4 (7.3%)	0 (0%)	20 (39.2%)	57 (15.8%)

## Appendix

Members of the MDS-endorsed PSP study group who contributed to the collection of the nongenetic case series: Thomas Arzberger, MD,^1,2^ Yaroslau Compta, MD,^3^ Elisabet Englund, MD,^4^ Leslie W. Ferguson, MD,^5^ Ellen Gelpi, MD,^6,7^ Sigrun Roeber, MD,^2^ Armin Giese, MD,^2^ Murray Grossman, MD,^8^ David J. Irwin, MD,^8^ Wassilios G. Meissner, MD, PhD,^9,10,11^ Christer Nilsson, MD,^4^ Alexander Pantelyat, MD,^12^ Alex Rajput, MD,^5^ John C. van Swieten, MD,^13^ and Claire Troakes, PhD, MSc.^14^

^1^Department of Psychiatry and Psychotherapy, University Hospital, Ludwig-Maximilians-University Munich, Munich, Germany.

^2^Center for Neuropathology and Prion Research, Ludwig-Maximilians-University Munich, Munich, Germany.

^3^Parkinson’s Disease & Movement Disorders Unit, Hospital Clínic/IDIBAPS/CIBERNED/European Reference Network for Rare Neurological Diseases (ERN-RND)/Institut de Nerociències, Universitat de Barcelona, Catalonia, Spain.

^4^Department of Clinical Sciences, Division of Neurology, Lund University, Lund, Sweden.

^5^Division of Neurology, Royal University Hospital, University of Saskatchewan, Saskatoon, Canada.

^6^Neurological Tissue Bank and Neurology Department, Hospital Clínic de Barcelona, Univesitat de Barcelona, IDIBAPS, CERCA, Barcelona, Catalonia, Spain.

^7^Institute of Neurology, Medical University of Vienna, Vienna, Austria.

^8^Frontotemporal Degeneration Center, Department of Neurology, University of Pennsylvania, Pennsylvania, USA.

^9^Univ. de Bordeaux, Institut des Maladies Neurodé-generatives, UMR 5293, Bordeaux, France.

^10^Service de Neurologie, Hôpital Pellegrin, CHU de Bordeaux, Bordeaux, France.

^11^Department of Medicine, University of Otago, Christchurch, and New Zealand Brain Research Institute, Christchurch, New Zealand.

^12^Department of Neurology, Johns Hopkins University, Baltimore, Maryland, USA.

^13^Department of Neurology, Erasmus Medical Centre, Rotterdam, The Netherlands.

^14^London Neurodegenerative Diseases Brain Bank, Institute of Psychiatry, Psychology and Neuroscience, Kings College London, London, UK.
