# Supplementary material for: Genotype–Phenotype Relations for the Atypical Parkinsonism Genes:MDSGene Systematic Review
Source: Mov Disord. Author manuscript; Available in PMC 2022 Jul 1. (PMC9070562; doi:10.1002/mds.28517)

**Supplement**

**1. Supplementary Methods**

**Supplementary Methods 1. Reference list of MDSGene for A) *ATP13A2*, B) *DNAJC6*, C) *FBXO7*, D) *SYNJ1*, E) *VPS13C,* and F) *DCTN1***

**A) *ATP13A2***

1. Pietrzak A, Badura-Stronka M, Kangas-Kontio T, Felczak P, Kozubski W, Latos-Bielenska A, Wierzba-Bobrowicz T, Florczak-Wyspianska J. Clinical and ultrastructural findings in an ataxic variant of Kufor-Rakeb syndrome. Folia Neuropathol. 2019;57(3):285-294.

2. McNeil-Gauthier AL, Brais B, Rouleau G, Anoja N, Ducharme S. Successful treatment of psychosis in a patient with Kufor-Rakeb syndrome with low dose aripiprazole: a case report. Neurocase 2019 Jun-Aug;25(3-4):133-137.

3. Inzelberg R, Estrada-Cuzcano A, Laitman Y, De Vriendt E, Friedman E, Jordanova A. Kufor-Rakeb Syndrome/PARK9: One Novel and One Possible Recurring Ashkenazi ATP13A2 Mutation. J Parkinsons Dis. 2018;8(3):399-403.

4. Bouhouche A, Tesson C, Regragui W, et al. Mutation Analysis of Consanguineous Moroccan Patients with Parkinson's Disease Combining Microarray and Gene Panel. Front Neurol. 2017 Oct 31;8:567.

5. Abbas MM, Govindappa ST, Sheerin UM, Bhatia KP, Muthane UB. Exome Sequencing Identifies a Novel Homozygous Missense ATP13A2 Mutation. Mov Disord Clin Pract. 2016 Apr 26;4(1):132-135.

6. van de Warrenburg BP, Schouten MI, de Bot ST, et al. Clinical exome sequencing for cerebellar ataxia and spastic paraplegia uncovers novel gene-disease associations and unanticipated rare disorders. Eur J Hum Genet. 2016 Oct;24(10):1460-1466.

7. Martino D, Melzi V, Franco G, Kandasamy N, Monfrini E, Di Fonzo A. Juvenile dystonia-parkinsonism syndrome caused by a novel p.S941Tfs1X ATP13A2 (PARK9) mutation. Parkinsonism Relat Disord. 2015 Nov;21(11):1378-1380.

8. Malakouti-Nejad M, Shahidi GA, Rohani M, et al. Identification of p.Gln858* in ATP13A2 in two EOPD patients and presentation of their clinical features. Neurosci Lett. 2014 Aug 8;577:106-111.

9. Bras J, Verloes A, Schneider SA, Mole SE, Guerreiro RJ. Mutation of the parkinsonism gene ATP13A2 causes neuronal ceroid-lipofuscinosis. Hum Mol Genet. 2012 Jun 15;21(12):2646-2650.

10. Eiberg H, Hansen L, Korbo L, et al. Novel mutation in ATP13A2 widens the spectrum of Kufor-Rakeb syndrome (PARK9). Clinical genetics 2012;82(3):256-263.

11. Lai HJ, Lin CH, Wu RM. Early-onset autosomal-recessive parkinsonian-pyramidal syndrome. Acta Neurol Taiwan 2012 Sept;21(3):99-107.

12. Park JS, Mehta P, Cooper AA, et al. Pathogenic effects of novel mutations in the P-type ATPase ATP13A2 (PARK9) causing Kufor-Rakeb syndrome, a form of early-onset parkinsonism. Hum Mutat 2011 Aug;32(8):956-964.

13. Crosiers D, Ceulemans B, Meeus B, et al. Juvenile dystonia-parkinsonism and dementia caused by a novel ATP13A2 frameshift mutation. Parkinsonism Relat Disord 2011 Febr;17(2):135-138.

14. Behrens MI, Brüggemann N, Chana P, Venegas P, Kägi M, Parrao T, Orellana P, Garrido C, Rojas CV, Hauke J, Hahnen E, González R, Seleme N, Fernández V, Schmidt A, Binkofski F, Kömpf D, Kubisch C, Hagenah J, Klein C, Ramirez A. Clinical spectrum of Kufor-Rakeb syndrome in the Chilean kindred with ATP13A2 mutations. Mov Disord. 2010 Sept 15;25(12):1929-1937.

15. Brüggemann N, Hagenah J, Reetz K, Schmidt A, Kasten M, Buchmann I, Eckerle S, Bähre M, Münchau A, Djarmati A, van der Vegt J, Siebner H, Binkofski F, Ramirez A, Behrens MI, Klein C. Recessively inherited parkinsonism: effect of ATP13A2 mutations on the clinical and neuroimaging phenotype. Arch Neurol. 2010 Nov;67(11):1357-1363.

16. Paisán-Ruiz C, Guevara R, Federoff M, Hanagasi H, Sina F, Elahi E, Schneider SA, Schwingenschuh P, Bajaj N, Emre M, Singleton AB, Hardy J, Bathia KP, Brandner S, Lees AJ, Houlden H. Early-onset L-dopa-responsive parkinsonism with pyramidal signs due to ATP13A2, PLA2G6, FBXO7 and spatacsin mutations. Mov Disord. 2010 Sept 15;25(12):1791-800.

17. Schneider SA, Paisán-Ruiz C, Quinn NP, Lees AJ, Houlden H, Hardy J, Bhatia KP. ATP13A2 mutations (PARK9) cause neurodegeneration with brain iron accumulation. Mov Disord. 2010 Jun 15;25(8):979-984.

18. Ning YP, Kanai K, Tomiyama H, et al. PARK9-linked parkinsonism in eastern Asia: mutation detection in ATP13A2 and clinical phenotype. Neurology 2008 Apr 15;70(16 Pt 2):1491-1493.

19. Di Fonzo A, Chien HF, Socal M, et al. ATP13A2 missense mutations in juvenile parkinsonism and young onset Parkinson disease. Neurology 2007 May 8;68(19):1557-1562.

20. Ramirez A, Heimbach A, Grundemann J, et al. Hereditary parkinsonism with dementia is caused by mutations in ATP13A2, encoding a lysosomal type 5 P-type ATPase. Nat Genet. 2006 Oct;38(10):1184-1191.

21. Williams DR, Hadeed A, al-Din AS, Wreikat AL, Lees AJ. Kufor Rakeb disease: autosomal recessive, levodopa-responsive parkinsonism with pyramidal degeneration, supranuclear gaze palsy, and dementia. Mov Disord. 2005 Oct;20(10):1264-1271.

22. Hampshire DJ, Roberts E, Crow Y, Bond J, Mubaidin A, Wriekat AL, Al-Din A, Woods CG. Kufor Rakeb syndrome, pallido-pyramidal degeneration with supranuclear upgaze paresis and dementia, maps to 1p36. J Med Genet. 2001 Oct;38(10):680-682.

23. Quinn NP, Goadsby PJ, Lees AJ. Hereditary juvenile parkinsonism with pyramidal signs and mental retardation. Eur J Neurol 1995 Mar;2(1):23-26.

24. Najim al-Din AS, Wriekat A, Mubaidin A, Dasouki M, Hiari M. Pallido-pyramidal degeneration, supranuclear upgaze paresis and dementia: Kufor-Rakeb syndrome. Acta Neurol Scand. 1994 May;89(5):347-352.

25. De Volder AG, Cirelli S, de Barsy T, Brucher JM, Bol A, Michel C, Goffinet AM. Neuronal ceroid-lipofuscinosis: preferential metabolic alterations in thalamus and posterior association cortex demonstrated by PET. J Neurol Neurosurg Psychiatry 1990 Dec;53(12):1063-1067.

26. Tomé FMS, Brunet P, Fardeau M, Hentati F, Reix J. Familial disorder of the central and peripheral nervous systems with particular cytoplasmic lamellated inclusions in peripheral nerves, muscle satellite cells, and blood capillaries. Acta Neuropathol. 1985;68:209-217.

27. Carlier G, Dubru JM. Familial juvenile Parkinsonism. Acta Paediatr Belg. 1979 Apr-Jun;32(2):123-127.

**B) *DNAJC6***

1. Elsayed LE, Drouet V, Usenko T, et al. A Novel Nonsense Mutation in DNAJC6 Expands the Phenotype of Autosomal-Recessive Juvenile-Onset Parkinson's Disease. Ann Neurol. 2016 Feb;79(2):335-337.

2. Olgiati S, Quadri M, Fang M, et al. DNAJC6 Mutations Associated With Early-Onset Parkinson's Disease. Ann Neurol. 2016 Feb;79(2):244-256.

3. Köroğlu Ç, Baysal L, Cetinkaya M, Karasoy H, Tolun A. DNAJC6 is responsible for juvenile parkinsonism with phenotypic variability. Parkinsonism Relat Disord. 2013 Mar;19(3):320-324.

4. Edvardson S, Cinnamon Y, Ta-Shma A, et al. A deleterious mutation in DNAJC6 encoding the neuronal-specific clathrin-uncoating co-chaperone auxilin, is associated with juvenile parkinsonism. PloS One 2012;7(5):e36458.

**C) *FBXO7***

1. Wei L, Ding L, Li H, et al. Juvenile-onset parkinsonism with pyramidal signs due to compound heterozygous mutations in the F-Box only protein 7 gene. Parkinsonism Relat Disord. 2018 Feb;47:76-79.

2. Conedera S, Apaydin H, Li Y, et al. FBXO7 mutations in Parkinson's disease and multiple system atrophy. Neurobiol Aging. 2016 Apr;40:192.e191-192.e195.

3. Lohmann E, Coquel AS, Honore A, et al. A new F-box protein 7 gene mutation causing typical Parkinson's disease. Mov Disord. 2015 Jul;30(8):1130-1133.

4. Gündüz A, Eken AG, Bilgic B, Hanagasi HA, Bilgüvar K, Günel, Basak AN, Ertan S. FBXO7-R498X mutation: phenotypic variability from chorea to early onset parkinsonism within a family. Parkinsonism Relat Disord. 2014 Nov;20(11):1253-1256.

5. Yalcin-Cakmakli G, Olgiati S, Quadri M, et al. A new Turkish family with homozygous FBXO7 truncating mutation and juvenile atypical parkinsonism. Parkinsonism Relat Disord. 2014 Nov;20(11):1248-1252.

6. Paisan-Ruiz C, Guevara R, Federoff M, et al. Early-onset L-dopa-responsive parkinsonism with pyramidal signs due to ATP13A2, PLA2G6, FBXO7 and spatacsin mutations. Mov Disord. 2010 Sept 15;25(12):1791-1800.

7. Di Fonzo A, Dekker MC, Montagna P, et al. FBXO7 mutations cause autosomal recessive, early-onset parkinsonian-pyramidal syndrome. Neurology 2009 Jan 20;72(3):240-245.

8. Shojaee S, Sina F, Banihosseini SS, Kazemi MH, Kalhor R, Shahidi GA, Fakhrai-Rad H, Ronaghi M, Elahi E. Genome-wide linkage analysis of a Parkinsonian-pyramidal syndrome pedigree by 500 K SNP arrays. Am J Hum Gen. 2008 Jun;82(6):1375-1384.

9. Hanagasi HA, Lees A, Johnson JO, Singleton A, Emre M. Smoking-responsive juvenile-onset Parkinsonism. Mov Disord. 2007 Jan;22(1):115-119.

**D) *SYNJ1***

1. Xie F, Chen S, Cen ZD, Chen Y, Yang DH, Wang HT, Zhang BR, Luo W. A novel homozygous SYNJ1 mutation in two siblings with typical Parkinson’s disease. Parkinsonism Relat Disord. 2019 Dec;69:134-137.

2. Hong D, Cong L, Zhong S, He Y, Xin L, Gao X, Zhang J. Clonazepam improves the symptoms of two siblings with novel variants in the SYNJ1 gene. Parkinsonism Relat Disord. 2019 May;62(221-225).

3. Romdhan S, Sakka S, Farhat N, Triki S, Dammak M, Mhiri C. A Novel SYNJ1 Mutation in a Tunisian Family with Juvenile Parkinson’s Disease Associated with Epilepsy. J Mol Neurosci. 2018 Oct;66(2):273-278.

4. Taghavi S, Chaouni R, Tafakhori A, et al. A Clinical and Molecular Genetic Study of 50 Families with Autosomal Recessive Parkinsonism Revealed Known and Novel Gene Mutations. Mol Neurobiol. 2018 Apr;55(4):3477-3489.

5. Rauschendorf MA, Jost M, Stock F, et al. Novel compound heterozygous synaptojanin-1 mutation causes l-dopa-responsive dystonia-parkinsonism syndrome. Mov Disord. 2017 Mar;32(3):478-480.

6. Kirola L, Behari M, Shishir C, Thelma BK. Identification of a novel homozygous mutation Arg459Pro in SYNJ1 gene of an Indian family with autosomal recessive juvenile Parkinsonism. Parkinsonism Relat Disord. 2016 Oct;31:124-128.

7. De Rosa A, Pellegrino T, Pappata S, et al. Non-motor symptoms and cardiac innervation in SYNJ1-related parkinsonism. Parkinsonism Related Disord. 2016 Feb;23:102-105.

8. Drouet V, Lesage S. Synaptojanin 1 mutation in Parkinson's disease brings further insight into the neuropathological mechanisms. Biomed Res. Int. 2014;2014:289728.

9. Olgiati S, De Rosa A, Quadri M, et al. PARK20 caused by SYNJ1 homozygous Arg258Gln mutation in a new Italian family. Neurogenetics 2014 Aug;15(3):183-188.

10. Picillo M, Ranieri A, Orefice G, Bonifati V, Barone P. Clinical progression of SYNJ1-related early onset atypical parkinsonism: 3-year follow up of the original Italian family. J Neurol. 2014 April;261(4):823-824.

11. Quadri M, Fang M, Picillo M, et al. Mutation in the SYNJ1 gene associated with autosomal recessive, early-onset Parkinsonism. Hum Mutat. 2013 Sep;34(9):1208-1215.

12. Krebs CE, Karkheiran S, Powell JC, et al. The Sac1 domain of SYNJ1 identified mutated in a family with early-onset progressive Parkinsonism with generalized seizures. Hum Mutat 2013 Sep;34(9):1200-1207.

***E*) *VPS13C***

1. Darvish H, Bravo P, Tafakhori A, et al. Identification of a large homozygous VPS13C deletion in a patient with early-onset Parkinsonism. Mov Disord. 2018 Dec;33(12):1968-1970.

2. Lesage S, Drouet V, Majounie E, et al. Loss of VPS13C Function in Autosomal-Recessive Parkinsonism Causes Mitochondrial Dysfunction and Increases PINK1/Parkin-Dependent Mitophagy. Am J Human Genet. 2016 Mar 3;98(3):500-513.

**F) *DCTN1***

1. Konno T, Ross OA, Teive HAG, Slawek J, Dickson DW, Wszolek ZK. DCTN1-related neurodegeneration: Perry syndrome and beyond. Parkinsonism Relat Disord. 2017 Aug;41:14-24.

2. Umemoto G, Tsuboi Y, Furuya H, et al. Dysphagia in Perry Syndrome: Pharyngeal Pressure in Two Cases. Cae Rep Neurol. 2017 Jun 14;9(2):161-167.

3. Perez-Soriano A, Arena JE, Dinelle K, et al. PBB3 imaging in Parkinsonian disorders: Evidence for binding to tau and other proteins. Mov Disord. 2017 Jul;32(7):1016-1024.

4. Gustavsson EK, Trinh J, Guella I, et al. DCTN1 p.K56R in progressive supranuclear palsy. Parkinsonism Relat Disord. 2016 Jul;28:56-61.

5. Mishima T, Fujioka S, Kurisaki R, et al. Impulse control disorders and punding in Perry syndrome. Parkinsonism Relat Disord. 2015 Nov;21(11):1381-1382.

6. Tacik P, Fiesel FC, Fujioka S, et al. Three families with Perry syndrome from distinct parts of the world. Parkinsonism Relat Disord. 2014 Aug;20(8):884-888.

7. Felicio AC, Dinelle K, Agarwal PA, et al. In vivo dopaminergic and serotonergic dysfunction in DCTN1 gene mutation carriers. Mov Disord. 2014 Aug;29(9):1197-1201.

8. Araki E, Tsuboi Y, Daechsel J, et al. A novel DCTN1 mutation with late-onset parkinsonism and frontotemporal atrophy. Mov Disord. 2014 Aug;29(9):1201-1204.

9. Pretelt F, Castaneda Cardona C, Tacik P, Ross OA, Wszolek ZK. Latin America's first case of Perry syndrome and a new treatment option for respiratory insufficiency. J Neurol 2014 May;261(3):620-621.

10. Chung EJ, Hwang JH, Lee MJ, et al. Expansion of the clinicopathological and mutational spectrum of Perry syndrome. Parkinsonism Relat Disord. 2014 Apr;20(4):388-393.

11. Caroppo P, Le Ber I, Clot F, et al. DCTN1 mutation analysis in families with progressive supranuclear palsy-like phenotypes. JAMA Neurol. 2014 Feb;71(2):208-215.

12. Aji BM, Medley G, O'Driscoll K, Larner AJ, Alusi SH. Perry syndrome: a disorder to consider in the differential diagnosis of Parkinsonism. J Neurol Sci. 2013 Jul;330(1-2):117-118.

13. Ohshima S, Tsuboi Y, Yamamoto A, et al. Autonomic failures in Perry syndrome with DCTN1 mutation. Parkinsonism Rel Disord. 2010 Nov;16(9):612-614.

14. Newsway V, Fish M, Rohrer JD, et al. Perry syndrome due to the DCTN1 G71R mutation: a distinctive levodopa responsive disorder with behavioral syndrome, vertical gaze palsy, and respiratory failure. Mov Disord. 2010 Apr;25(6):767-770.

15. Saka E, Topcuoglu MA, Demir AU, Elibol B. Transcranial sonography in Perry syndrome. Parkinsonism Relat Disord. 2010 Jan;16(1):68-70.

16. Wider C, Dachsel JC, Farrer MJ, Dickson DW, Tsuboi Y, Wszolek ZK. Elucidating the genetics and pathology of Perry syndrome. J Neurol Sci. 2010 Feb;289(1-2):149-154.

17. Farrer MJ, Hulihan MM, Kachergus JM, et al. DCTN1 mutations in Perry syndrome. Nat Genet. 2009 Feb;41(2):163-165.

18. Wider C, Dickson DW, Stoessl AJ, et al. Pallidonigral TDP-43 pathology in Perry syndrome. Parkinsonism Relat Disord. 2009 May;15(4):281-286.

19. Tsuboi Y, Wszolek ZK, Kusuhara T, Doh-ura K, Yamada T. Japanese family with parkinsonism, depression, weight loss, and central hypoventilation. Neurology 2002 Apr 9;58(7):1025-1030.

20. Bhatia KP, Daniel SE, Marsden CD. Familial parkinsonism with depression: a clinicopathological study. Ann Neurol. 1993 Dec;34(6):842-847.

21. Roy EP, 3rd, Riggs JE, Martin JD, Ringel RA, Gutmann L. Familial parkinsonism, apathy, weight loss, and central hypoventilation: successful long-term management. Neurology 1988 Apr;38(4):637-639.

22. Purdy A, Hahn A, Barnett HJ, et al. Familial fatal Parkinsonism with alveolar hypoventilation and mental depression. Ann Neurol. 1979 Dec;6(6):523-531.

23. Perry TL, Bratty PJ, Hansen S, Kennedy J, Urquhart N, Dolman CL. Hereditary mental depression and Parkinsonism with taurine deficiency. Arch Neurol. 1975 Feb;32(2):108-113.

**Supplementary Methods 2. Pathogenicity scoring implemented in MDSGene**

Potential pathogenicity of reported variants was classified as “possible”, “probable”, or “definite” based on the following criteria: i) co-segregation with disease in the reported pedigrees and/or the number of reported mutation carriers, ii) frequency in ~120,000 ethnically diverse individuals from the gnomAD (Genome Aggregation Database) browser (http://gnomad.broadinstitute.org/), iii) CADD (“Combined Annotation Dependent Depletion") score as an in-silico measure of deleteriousness of genetic variants [Kircher et al. (2014). “A general framework for estimating the relative pathogenicity of human genetic variants”. Nat Genet. 2014. 46:310-315. DOI: 10.1038/ng.2892, Rentzsch et al. (2018). “CADD: predicting the deleteriousness of variants throughout the human genome”. Nucleic Acids Res. 2018 Oct 29. DOI: 10.1093/nar/gky1016 ] , and iv) reported molecular evidence from in-vivo and/or in-vitro studies. Each evidence domain was divided into four categories each accumulating specific “points”, weighted by category (see **Supplementary Table 3**). Evidence domains “co-segregation with disease” and “presence of mutation-specific positive functional data” received the strongest weights in the pathogenicity grading. Finally, points were summed across categories and pathogenicity was graded as follows: benign (<5 points), possibly pathogenic (5-9 points), probably pathogenic (10-14 points), definitely pathogenic (>14 points). Reported genetic variants that have been classified as benign using this scoring algorithm are not included in MDSGene (https://www.mdsgene.org/methods).

**Supplementary Methods 3. Detailed descriptions for the automated classification of monogenic atypical parkinsonism forms by means of clinical signs**

For automated classification of monogenic atypical parkinsonism forms by means of their clinical signs, we used an ensemble of bootstrap-aggregated (‘bagged’) decision trees as implemented in the so-called ‘TreeBagger’ method of MATLAB (R2020a; The Math Works, Inc., Natick, MA, USA). Every tree in this ensemble is grown on an independently drawn bootstrap replica of the training data. Observations not included in this replica (usually 1/3 of all observations) are ‘out of bag’ for this tree and can later be employed for validation, i.e., the ‘out-of-bag’ error is used as an estimate of the generalization error. In addition to the randomization of training *cases*, the training *variables* (here: the clinical signs) are also randomly selected from a subfraction of all possible variables for each decision split at the tree nodes during the growth of the trees. In summary, this corresponds to the ‘Random Forests’ or ‘Random Decision Forests’ method described by L. Breiman and A. Cutler [Breiman L *(2001). "Random Forests".* Machine Learning.*45 (1): 5–32.* DOI*:* 10.1023/A:1010933404324*.*]. Compared to normal decision trees, building such a forest of uncorrelated decision trees based on randomly selected training cases and variables has the advantage of offering more resistance to overtraining and overfitting [*Kleinberg E (1996). "An Overtraining-Resistant Stochastic Modeling Method for Pattern Recognition".* Annals of Statistics*. 24 (6): 2319–2349.* DOI*:* 10.1214/aos/1032181157]. After training, it can be used to predict a previously unseen patient's class affiliation, based on the majority decision of the included decision trees.

In the present study, 67 clinical variables of 140 patients from 6 diagnosis groups were used to train the classifier described above. ‘Age at onset’ of disease (in years) was the only numerical variable, ‘levodopa response quantification’ was the only variable with relative grading (‘unknown’, ‘minimal/intermittent’, ‘mild’, ‘moderate’, ‘good/excellent’); all others were categorical and encoded the presence or absence of a specific clinical sign (e.g., symptoms of sleep disorder, cognitive decline, incontinence, etc. (**Supplementary Table 2**). It is noteworthy that the table of clinical variables was only sparsely filled; i.e., in many cases, no information on the presence or absence of the particular clinical sign was available. On average and per clinical variable, data was missing in 78% of patients (range: 8-99%). Nevertheless, the answer category ‘unknown’ was utilized at the nodes of the decision trees like a third possibility, besides ‘yes’ or ‘no’ for the presence or absence of a certain clinical sign. This is because the absence of specific information also carries information. For example, an accumulation of the response category ‘unknown’ in a particular diagnosis group might reflect that the clinical sign in question is not particularly typical or discriminatory for this diagnosis.

The specific implementation of the ‘TreeBagger’ method as classifier in our study utilized the following (mostly MATLAB default) parameters:

- Minimum number of observations per tree leaf = 1 (MATLAB default for classification)
- Number of variables to select at random for each decision split at the tree nodes = square root of the total number of variables, rounded up to next integer value (MATLAB default for classification)
- Prior probabilities for each class = 'Empirical' (this determines the prior class probabilities from the actual frequencies of the diagnosis groups; MATLAB default)
- Number of decision trees = 1000 (this number was chosen because preliminary tests with different numbers of decision trees had shown that more than 1000 trees did not lead to a relevant improvement of the classification accuracy)

Analysis 1: To estimate the accuracy of the trained classifier for new and unseen data, the classification performance was determined in two different ways:

1. Every tree in this ensemble is grown on an independently drawn bootstrap replica of the training data. Observations not included in this replica (usually 1/3 of all observations) are ‘out of bag’ (OOB) for this tree and can later be employed for validation, i.e. the OOB error is used as an estimate of the generalization error. From predictions for OOB observations, i.e. for a specific patient to classify, the prediction of the trained ensemble classifier was determined only from those decision trees which have not been trained with the patient data
2. By means of classical leave-one-out cross-validation (LOOCV)

We have chosen to compare these two approaches because there is an ongoing discussion whether classification with ‘Random Forests’ requires cross-validation or whether relying on the predictions for the OOB observations is enough to estimate the performance in new data (e.g. cf. https://datascience.stackexchange.com/questions/6510/does-modeling-with-random-forests-require-cross-validation). Both approaches (OOB and LOOCV) were repeated 20 times, with permutated order of training cases, to obtain average performance measures. Total accuracy (TA) and balanced accuracy (BA) were used together with 95% confidence intervals to assess the overall classification performance of the trained classifier. Total accuracy was defined as the proportion of true results among the total number of classifications. In contrast, balanced accuracy is the average sensitivity of all classes / diagnosis groups and provides additional information as it avoids the overestimation of classification performance attributed to imbalanced class sizes. True positive rates (TPR; i.e., recall or sensitivity), false-negative rates (FNR), positive predictive values (PPV; i.e., precision), and false discovery rates (FDR) were calculated for the individual diagnosis groups.

Analysis 2: To assess the importance of the different clinical signs for classification, we determined a dimensionless relative measure of importance for each predictor variable. For any variable, this measure (called ‘OOBPermutedPredictorDeltaError’ in MATLAB´s ‘TreeBagger’ method) is the increase in prediction error if the values of that variable are permuted across the out-of-bag observations (so that this random distribution removes any possible correlation between the clinical sign in question and a particular diagnosis group). The measure is computed for every tree, then averaged over the entire ensemble, and divided by the standard deviation over the entire ensemble. The whole analysis was repeated 100 times, with subsequent averaging of results.

**Supplementary Methods 4. Additional, exploratory analyses to classify monogenic atypical parkinsonism**

When repeating analysis 1 based only on the five most important clinical variables, TA and BA were reduced to 76.5% (95%CI: 76.4-76.5%) and 56.1% (95%CI: 56.0-56.1%), respectively. The sensitivities (TPR) for the single diagnosis groups were 86% for *ATP13A2*, 98% for *DCTN1*, 36% for *DNAJC6*, 81% for *FBXO7*, 35% for *SYNJ1* and 0% for *VPS13C*. Using the 10 most important variables (listed above), TA and BA were 86.5% (95%CI: 86.3-86.7%) and 73.7% (95%CI: 73.5-73.8%), respectively, and the sensitivities for the diagnosis groups amounted to 83% for *ATP13A2*, 100% for *DCTN1*, 73% for *DNAJC6*, 96% for *FBXO7*, 65% for *SYNJ1* and 25% for *VPS13C*.

When repeating analysis 1 with prior class probability set to ‘Uniform’ (i.e., without prior knowledge about class probabilities/frequency of diagnoses), TA minimally dropped (by less than 1%) to 90.4% (95%CI: 90.0-90.7%), while BA increased substantially (by about 5%) to a total of 86.6% (95%CI: 86.2-87.0%), respectively. The sensitivities for the underlying diagnosis groups amounted to 92% for *ATP13A2*, 94% for *DCTN1*, 82% for *DNAJC6*, 89% for *FBXO7*, 88% for *SYNJ1*, and 75% for *VPS13C*. Hence, the use of prior knowledge about the frequencies of the diagnosis groups, as was the case for analyses 1-3, did not provide any undue advantage and did not relevantly distort the performance results upwards.

**2. Supplementary Tables**

**Supplementary Table 1. Search terms for the literature search in PubMed (February 12^th^, 2020)**

| **Disease-Gene** | **Search term** |
| --- | --- |
| PARK- *ATP13A2* | (dystonia OR parkinson* OR ataxi* OR cerebellar OR “spastic paraplegia” OR “paroxysmal movement” OR tremor OR myoclon* OR chorea OR choreo* OR “spastic paraplegia” OR “spastic paraparesis” OR HSP OR “movement disorder” OR dyskinesia OR hyperkine* OR Strümpell* OR “Kufor-Rakeb Syndrome”) AND (HSA9947 OR “ATPase Cation Transporting 13A2” OR “ATPase 13A2” OR “ATPase Type 13A2” OR PARK9 OR ATP13A2 OR CLN12 OR KRPPD OR SPG78 OR 1p36) AND "english"[Language] |
| PARK-*DNAJC6* | (dystonia OR parkinson* OR ataxi* OR cerebellar OR “spastic paraplegia” OR “paroxysmal movement” OR tremor OR myoclon* OR chorea OR choreo* OR “spastic paraplegia” OR “spastic paraparesis” OR HSP OR “movement disorder” OR dyskinesia OR hyperkine* OR Strümpell*) AND (DNAJC6 OR DJC6 or PARK19 or “DnaJ (Hsp40) Homolog, Subfamily C, Member 6“ OR Auxilin OR “DnaJ (Hsp40) Homolog, Subfamily B, Member 6“ OR “DnaJ Homolog Subfamily C Member 6“ or KIAA0473 OR 1p31) AND "english"[Language] |
| PARK-*FBXO7* | (dystonia OR parkinson* OR ataxi* OR cerebellar OR “spastic paraplegia” OR “paroxysmal movement” OR tremor OR myoclon* OR chorea OR choreo* OR “spastic paraplegia” OR “spastic paraparesis” OR HSP OR “movement disorder” OR dyskinesia OR hyperkine* OR Strümpell*) AND (“F-Box Protein 7” OR “F-Box Only Protein 7” OR FBX7 OR PARK15 OR FBX07 OR PKPS OR FBX OR 22q12) AND "english"[Language] |
| PARK-*SYNJ1* | (dystonia OR parkinson* OR ataxi* OR cerebellar OR “spastic paraplegia” OR “paroxysmal movement” OR tremor OR myoclon* OR chorea OR choreo* OR “spastic paraplegia” OR “spastic paraparesis” OR HSP OR “movement disorder” OR dyskinesia OR hyperkine* OR Strümpell*) AND (SYNJ1 OR EIEE53 OR INPP5G OR PARK20 OR “Synaptojanin 1” OR “Phosphoinositide 5-Phosphatase” OR “Inositol Polyphosphate-5-Phosphatase G” OR 21q22) AND "english"[Language] |
| PARK-*VPS13C* | (ataxia OR ataxic OR cerebellar OR channelopathy OR dystonia OR dystonic OR parkinson* OR “paroxysmal movement” OR tremor OR myoclon* OR chorea OR choreo* OR choreatic OR “spastic paraplegia” OR” spastic paraparesis” OR HSP OR Strümpell* OR hyperkinetic OR “movement disorder” OR dyskinesia OR dyskinetic) AND (PARK23 OR VPS13C OR “Vacuolar Protein Sorting 13 Homolog C” OR “Vacuolar Protein Sorting-Associated Protein 13C” OR 15q22) AND "english"[Language] |
| PARK-*DCTN1* | (ataxia OR ataxic OR cerebellar OR channelopathy OR dystonia OR dystonic OR parkinson* OR paroxysmal movement OR tremor OR myoclon* OR chorea OR choreo* OR choreatic OR spastic paraplegia OR spastic paraparesis OR HSP OR Strümpell* OR hyperkinetic OR “movement disorder” OR dyskinesia OR dyskinetic OR “Perry syndrome” OR “amyotrophic lateral sclerosis”) AND (DCTN1 OR DAP-150 OR DP-150 OR P135 OR dynactin OR “p150, glued homologue” OR 2p13.1) AND "english"[Language] |

**Supplementary Table 2. List of variables: Demographic, clinical and genetic information extracted using the MDSGene protocol**

| **Patient characterization** |
| --- |
| individual patient ID |
| family ID |
| **Demographic information** |
| ethnicity |
| country of origin |
| sex |
| **Clinical information** |
| general |
| positive family history for the mutation-associated phenotype |
| consanguinity |
| age at examination in years |
| age at disease onset in years |
| disease duration in years |
| age at clinical diagnosis in years |
| age at death |
| motor signs and symptoms |
| parkinsonism |
| bradykinesia |
| rigidity |
| tremor (in general) |
| rest tremor |
| action tremor |
| postural tremor |
| dystonic tremor |
| postural instability |
| dyskinesia |
| dystonia |
| (mini)myoclonus |
| presence of atypical signs and symptoms |
| primitive reflexes |
| hyperreflexia |
| diurnal fluctuations as motor symptom fluctuations throughout the day |
| oculugyric spasms |
| saccadic abnormalities |
| jerky pursuit |
| gaze palsy |
| swallowing difficulty |
| swallowing disorder |
| hypophonia |
| dysarthria or anarthria |
| hypoventilation |
| hypomimia |
| apraxia |
| micrographia |
| ataxia dysdiadochokinesia |
| spasticity |
| dysphagia |
| gait difficulties |
| sleep benefit as restoration of mobility upon awakening from sleep |
| levodopa responsiveness |
| levodopa response quantification classified as minimal/intermittent, moderate or good/excellent |
| motor fluctuations in context of medication-intake |
| motor instrument like UPDRSIII or Hoehn&Yahr and, if available, their scoring |
| asymmetric onset |
| reported initial signs and symptoms |
| non-motor signs and symptoms |
| presence of non-motor signs and symptoms associated with parkinsonism |
| non-motor symptoms instrument |
| seizures |
| cataracts |
| olfactory impairment |
| incontinence |
| pes cavus |
| weight loss |
| autonomic symptoms |
| apathy or fatique |
| sleep disorder |
| behavioral abnormalities |
| impulsive control disorder |
| tics |
| cognitive decline |
| development delay |
| intellectual developmental disorder |
| psychotic symptoms |
| psychosis scale |
| depression |
| depression scale |
| hallucinations |
| anxiety |
| anxiety scale |
| **Genetic information** |
| genetic status of all tested family members with number of homozygous, heterozygous and wildtype ones in affected and not affected state |
| gene to which the mutation refers |
| physical location of the mutation (on the plus strand) |
| observed mutated bases on the plus strand |
| reference bases on the plus strand |
| genomic description of the mutation according to the nomenclature of the human genome variation society (HGVS) |
| coding description of the mutation according to the nomenclature of the HGVS |
| protein description of the mutation according to the nomenclature of the HGVS |
| genotype of the respective mutation |
| type of mutation |
| highest minor allel frequency (MAF) of the respective mutation found in gnomAD v.2.1.1 |
| pathogenicity status classified as benign, possibly, probably or definitely pathogenic |
| CADD score as one parameter for pathogenicity scoring |

**Supplementary Table 3. Pathogenicity scoring scheme implemented in MDSGene**

| **Gene** | **Chr. position** | **DNA change** | **Amino acid change** | **CADD score** | **Frequency (gnomAD)** | **Segregation points** | **Number of functional studies** |
| --- | --- | --- | --- | --- | --- | --- | --- |
| ATP13A2 | 1:17326989 | c.746C>T | p.Ala249Val | 3 | 8.02E-03 | 0/6 | 0 |
| ATP13A2 | 1:17313330 | c.3205G>A | p.Ala1069Thr | 13 | 2.65E-03 | 0/6 | 0 |
| DNAJC6 | 1:65855392 | c.1468+83del | Intronic | 0 | 9.18E-03 | 2/6 | 0 |
| SYNJ1 | 21:34012088 | c.3707C>T | p.Thr1236Met | 2 | 2.37E-03 | 2/6 | 0 |

**Supplementary Table 4. List of variants that were scored as benign and subsequently excluded from analyses. Segregation was scored as previously described (http://www.mdsgene.org/methods).**

**Supplementary Table 5. Overview of the median age at onset (IQR) in non-genetic atypical and monogenic typical PD/ atypical parkinsonism**

| **Type of PD** | **Mode of inheritance** | **Gene** | **Number of patients** | **Median age at onset (IQR)** | **Degree of missing data** |
| --- | --- | --- | --- | --- | --- |
| Atypical parkinsonism | Recessive | *ATP13A2* | 34 | 14 (12-17) | 5.6% |
|  |  | *DNAJC6* | 11 | 11 (10-29) | 0.0% |
|  |  | *FBXO7* | 17 | 17 (14-21) | 34.6% |
|  |  | *SYNJ1* | 17 | 22 (16-28) | 0.0% |
|  |  | *VPS13C* | 2 | 29 (25-33) | 50% |
|  |  | **All** | **81** | **16 (12-22)** | **13.8%** |
|  | Dominant | ***DCTN1*** | **46** | **49 (46-54)** | **0.0%** |
|  | Non-monogenic | ***none*** | **342** | **64 (57-70)** | **5.5%** |
| Typical PD | Recessive | *Parkin* | 1000 | 31 (23-38) |  |
|  |  | *PINK1* | 151 | 32 (25-40) |  |
|  |  | *DJ1* | 33 | 27 (22-34) |  |
|  |  | **All** | **1184** | **31 (24-38)** | **10.0%** |
|  | Dominant | *SNCA* | 146 | 46 (36-54) |  |
|  |  | *LRRK2* | 724 | 57 (47-65) |  |
|  |  | *VPS35* | 67 | 52 (45-61) |  |
|  |  | **All** | **870** | **55 (46-63)** | **7.2%** |

**Supplementary Table 6. Summary of different types of pathogenicity per gene and number of mutations**

| **Gene** | **Definitely pathogenic** | **Probably pathogenic** | **Possibly pathogenic** | **Number of mutations/gene** |
| --- | --- | --- | --- | --- |
| *ATP13A2* | 7 (35.0%) | 13 (65.0%) | 0 | 20 |
| *DNAJC6* | 0 | 4 (80.0%) | 1 (20%) | 5 |
| *SYNJ1* | 1 (11.1%) | 7 (77.8%) | 1 (11.1%) | 9 |
| *FBXO7* | 3 (42.9%) | 4 (57.1%) | 0 | 7 |
| *VPS13C* | 0 | 6 (100%) | 0 | 6 |
| *DCTN1* | 2 (20.0%) | 6 (60.0%) | 2 (20.0%) | 10 |
| **Number of mutation/type of pathogenicity** | 13 (22.8%) | 40 (70.1%) | 4 (7.1%) | 57 |

**Supplementary Table 7 A. Initial signs and symptoms in monogenic typical PD/atypical parkinsonism**

| **Type of PD** | **Mode of inheritance** | **Most frequent initial signs/symptoms** |
| --- | --- | --- |
| Atypical parkinsonism | Recessive | Bradykinesia (58%), n=42 |
|  |  | Tremor (33%), n=24 |
|  |  | Rigidity (22%), n=16  *Missing data: n=21* |
|  | Dominant | Depression (33%), n=10 |
|  |  | Apathy/fatigue (33%), n=10 |
|  |  | Bradykinesia (27%), n=8  *Missing data: n=16* |
| Typical PD | Recessive | Tremor (65%), n=216 |
|  |  | Bradykinesia (25%), n=84 |
|  |  | Dystonia (15%), n=51  *Missing data: n=849* |
|  | Dominant | Tremor (59%), n=301 |
|  |  | Bradykinesia (41%), n=211 |
|  |  | Rigidity (19%), n=98  *Missing data: n=427* |

**Supplementary Table 7 B. Levodopa response in monogenic typical PD and atypical parkinsonism**

| **Type of PD** | **Mode of inheritance** | **Good/**  **excellent** | **Moderate** | **Minimal** |
| --- | --- | --- | --- | --- |
| Typical PD | Dominant | 340 (93%) | 12 (3%) | 14 (4%) |
|  | Recessive | 281 (92%) | 14 (5%) | 9 (3%) |
|  | **Total** | **621 (93%)** | **26 (4%)** | **23 (3%)** |
| Atypical parkinsonism | Dominant | 8 (36%) | 8 (36%) | 6 (28%) |
|  | Recessive | 27 (54%) | 15 (30%) | 8 (16%) |
|  | **Total** | **35 (49%)** | **23 (26%)** | **14 (16%)** |

**3. Supplementary Figures**

**Supplementary Figure 1. Systematic screening for eligible records.**


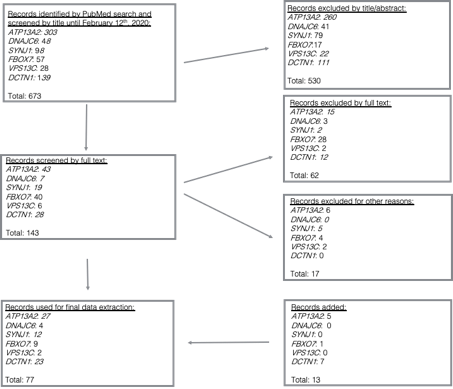


Figure legend. Inclusion and exclusion of manuscripts is documented for each level of screening (title, abstract, full text). Date of the literature search: February 12^th^, 2020

**Supplementary Figure 2. Distribution of age at onset and initial signs and symptoms in monogenic atypical parkinsonism patients**

| **A) *ATP13A2*** | |
| --- | --- |
| 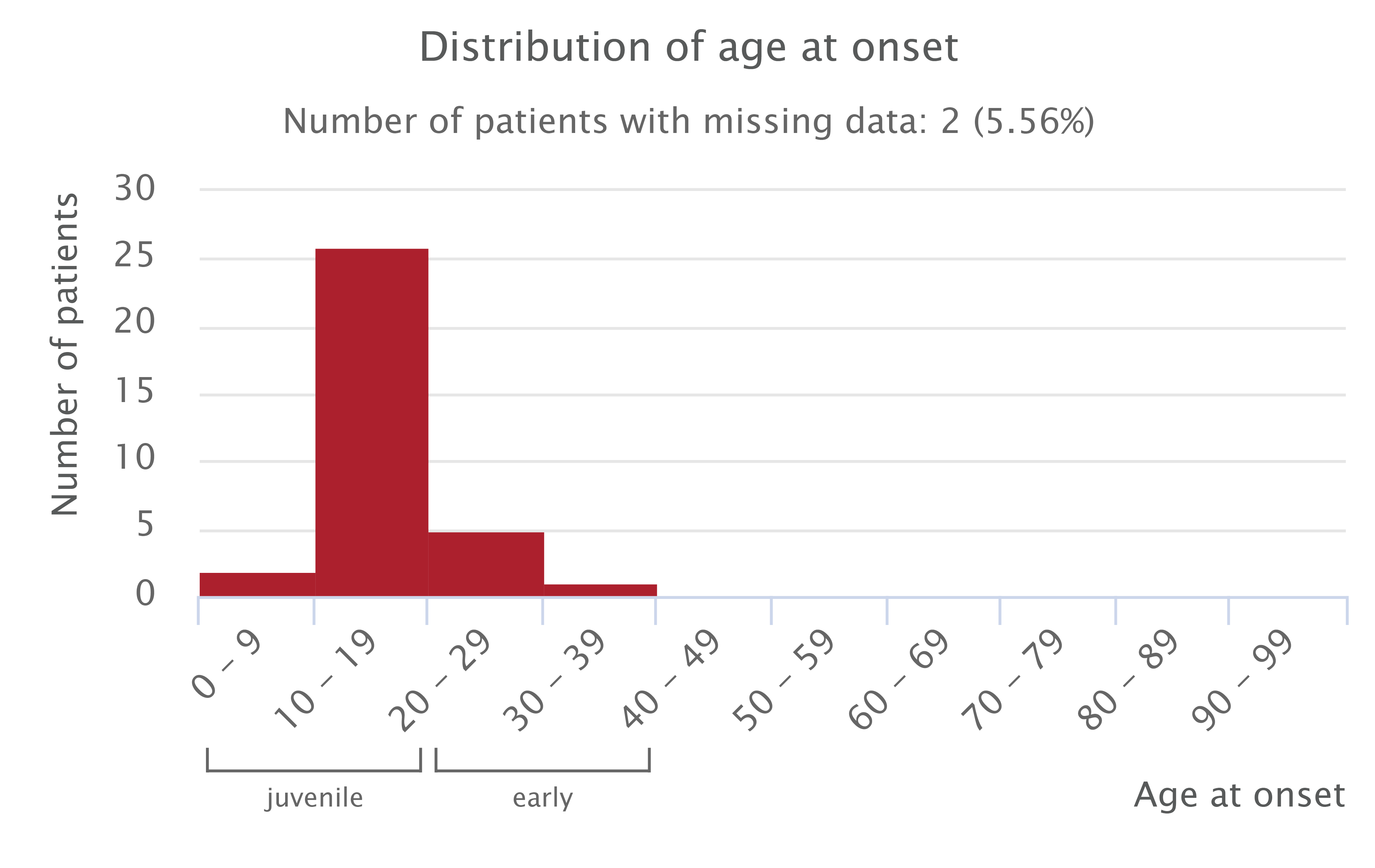 | 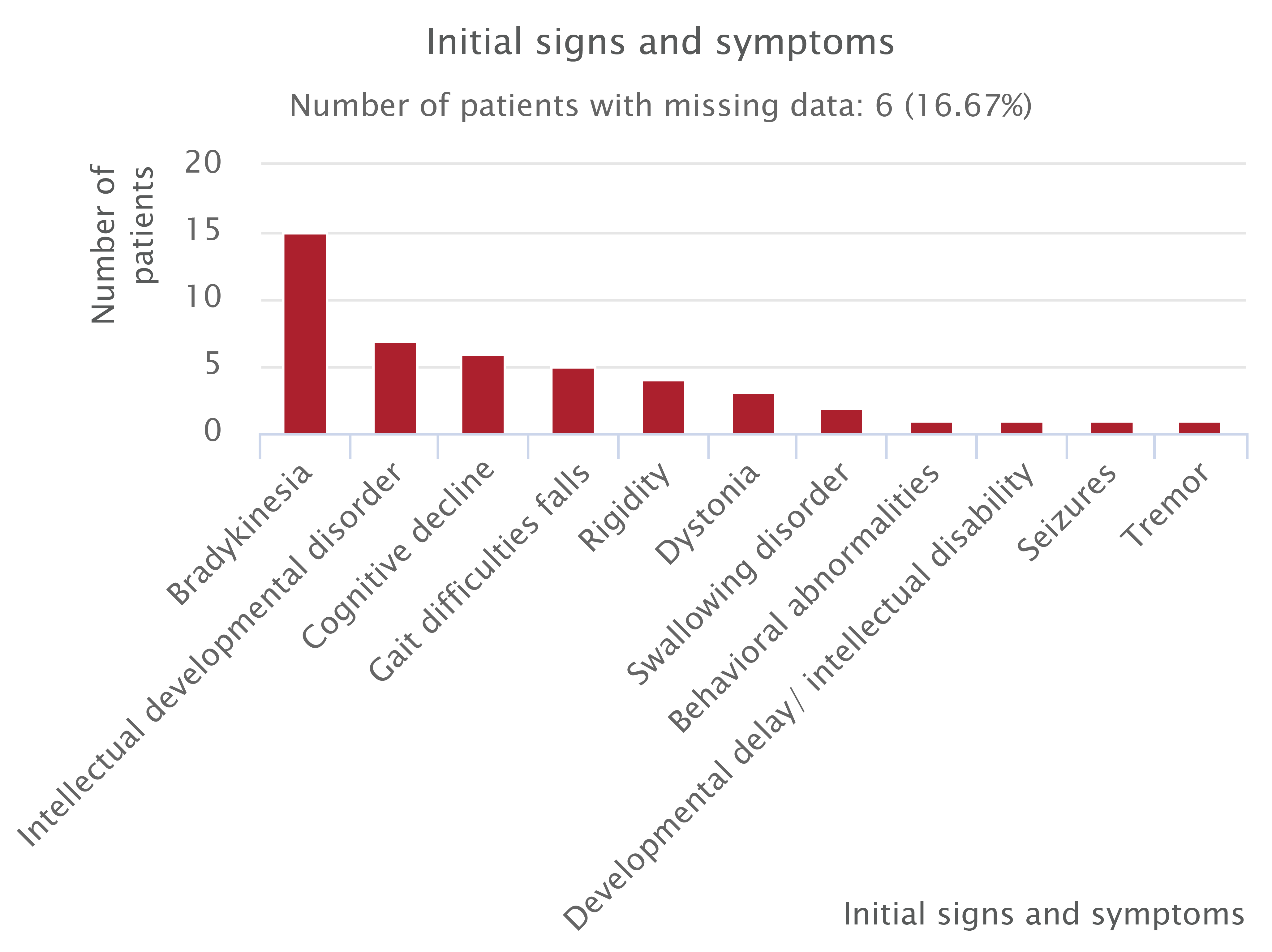 |
| **B) *DNAJC6*** | |
| 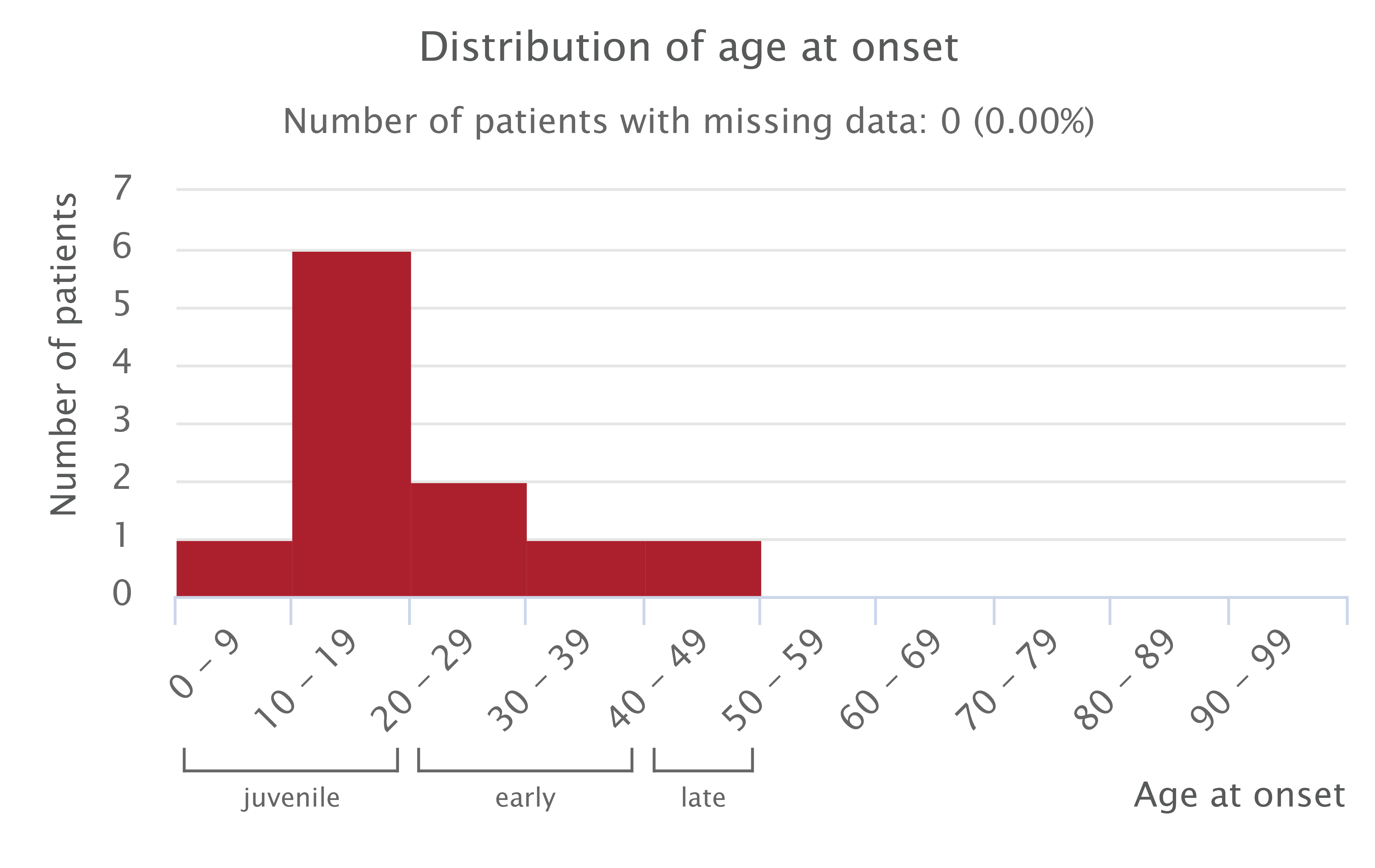 | 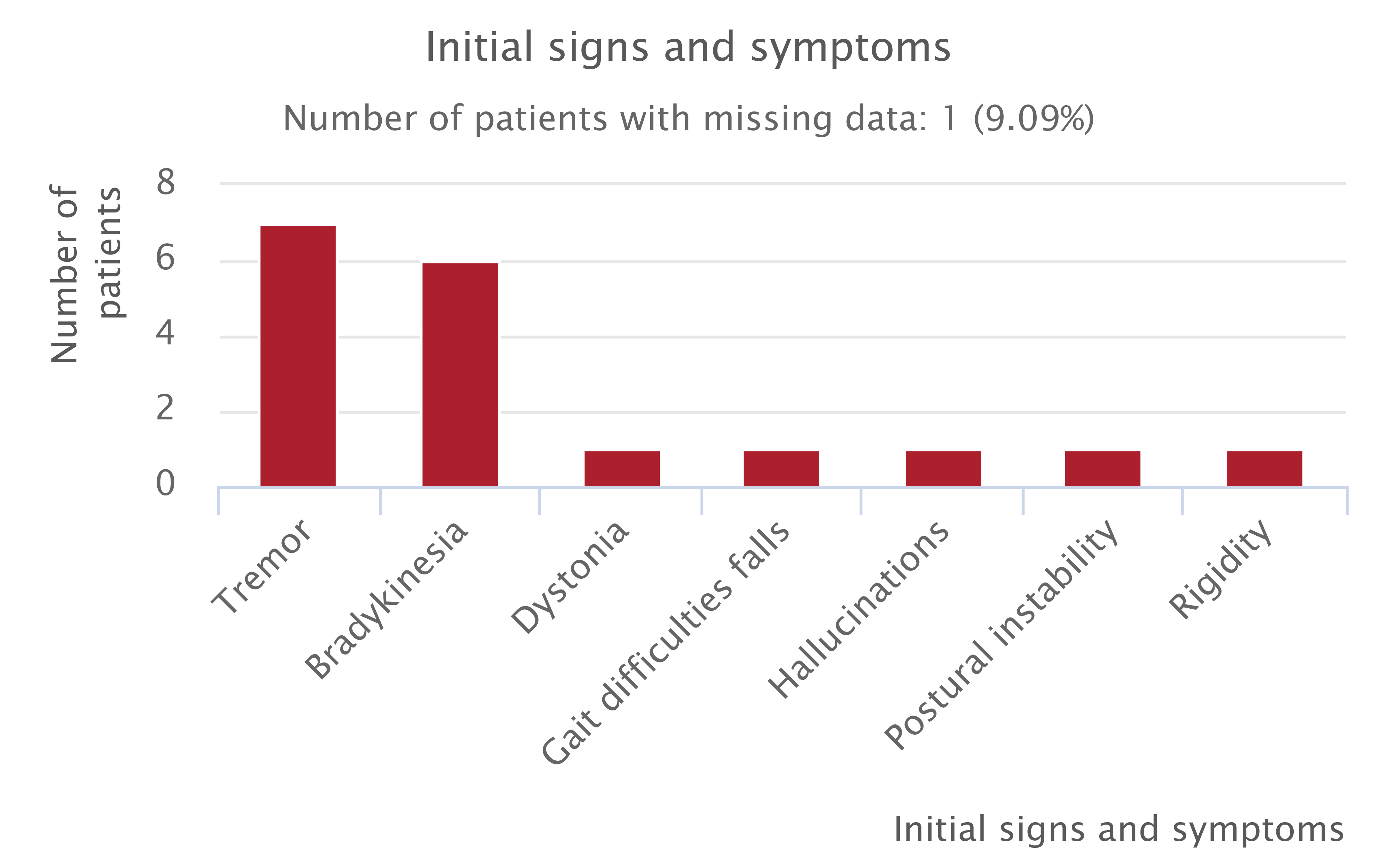 |
| **C) *FBXO7*** | |
| 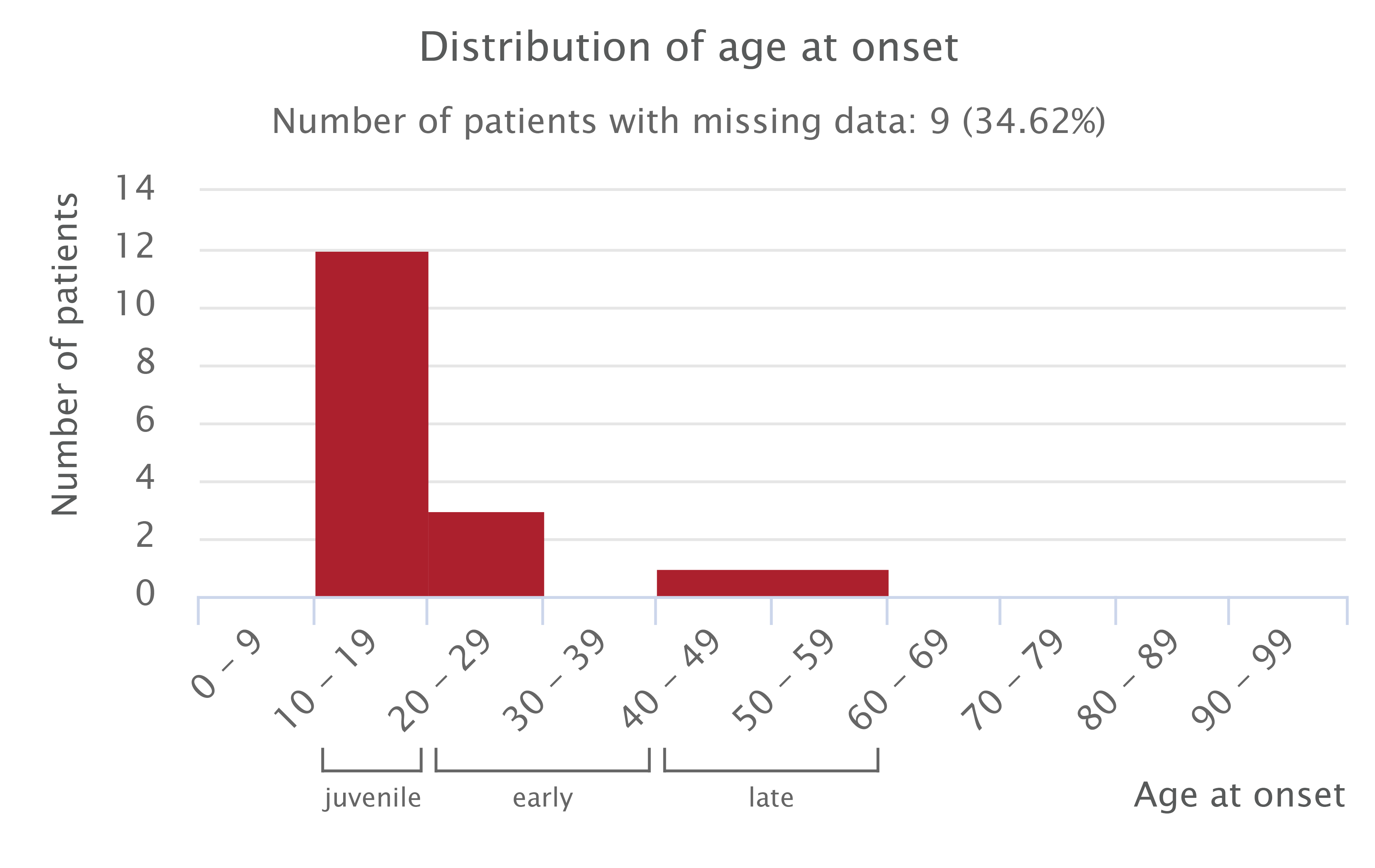 | 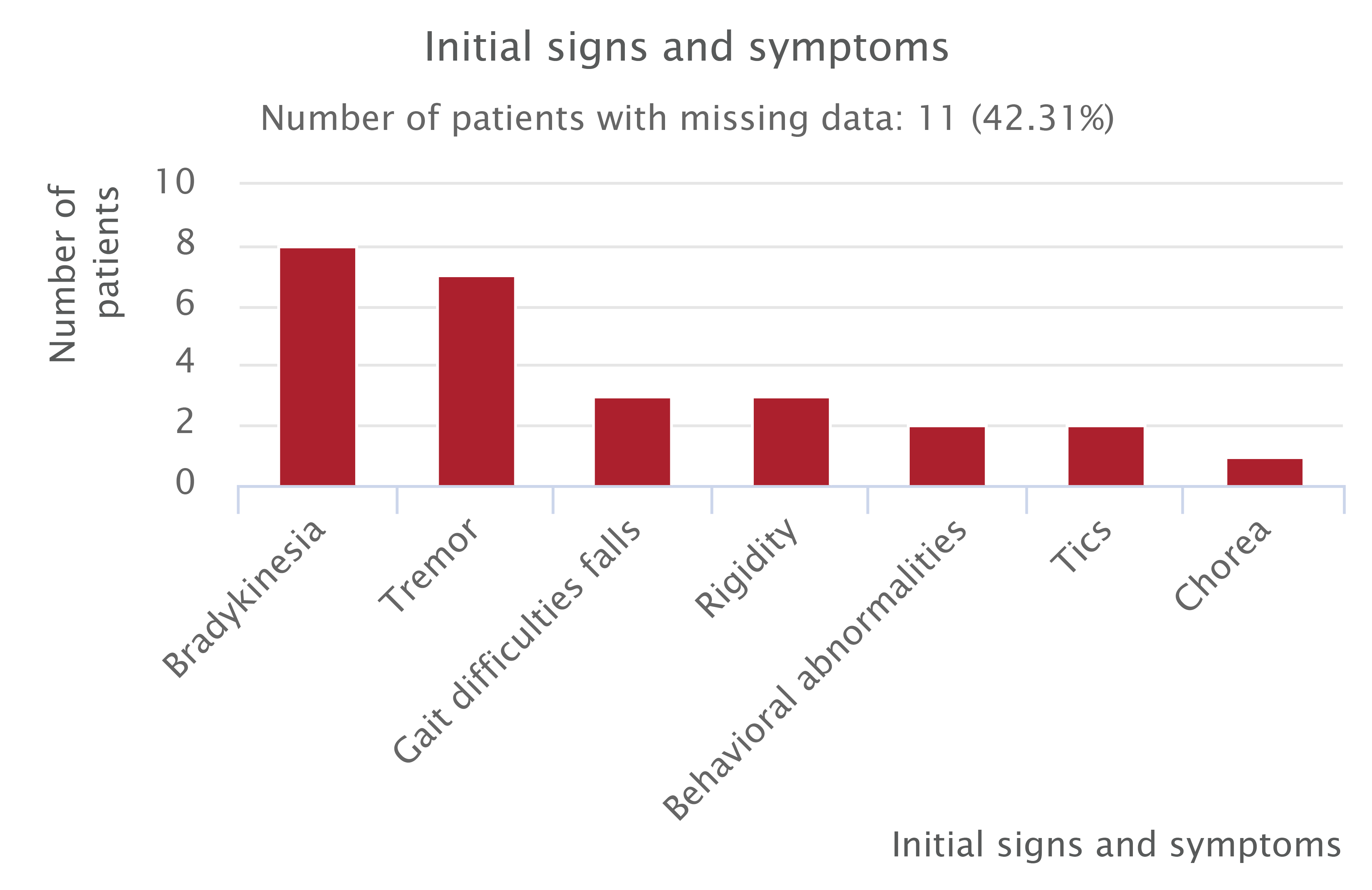 |
| **D) *SYNJ1*** | |
| 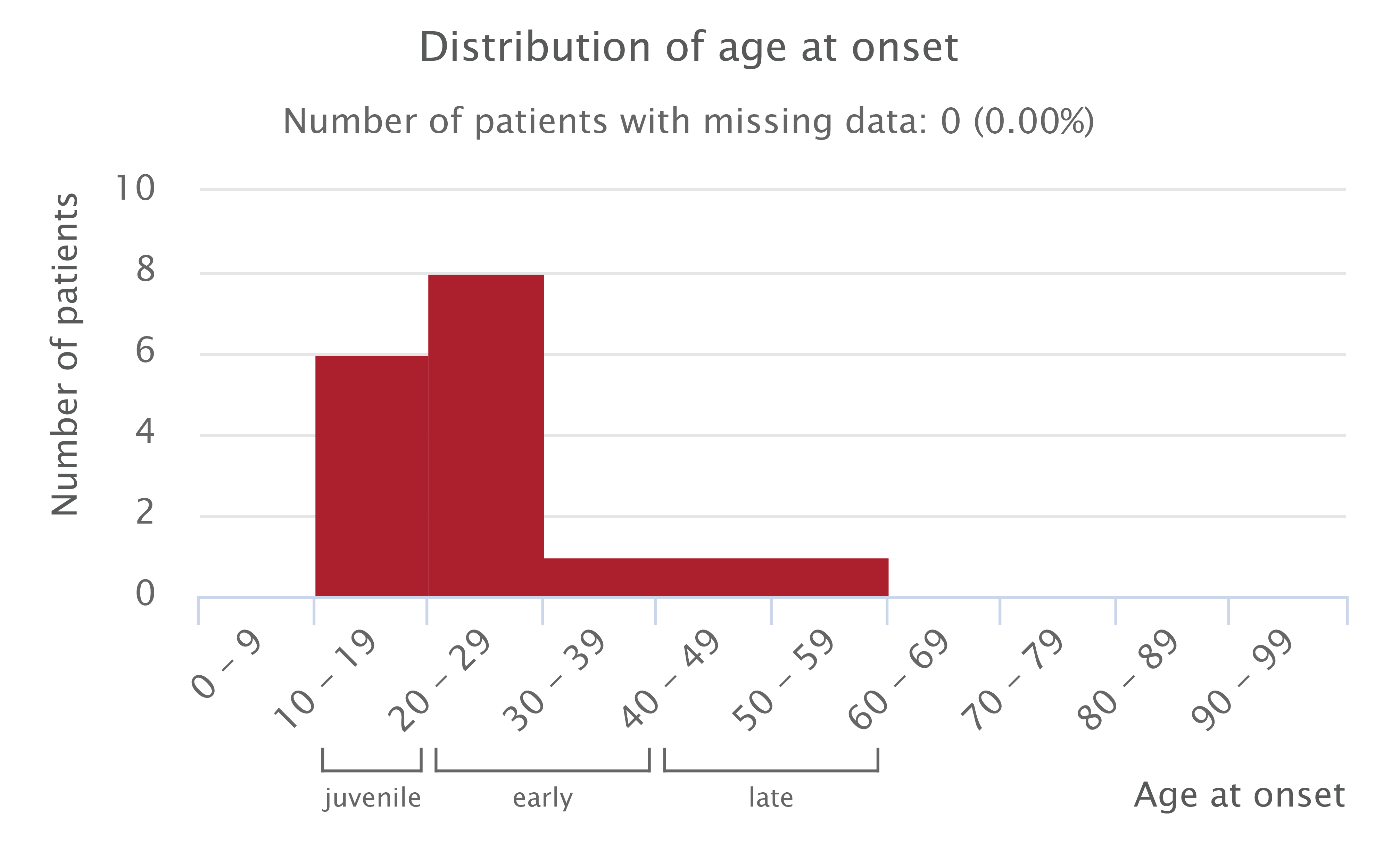 | 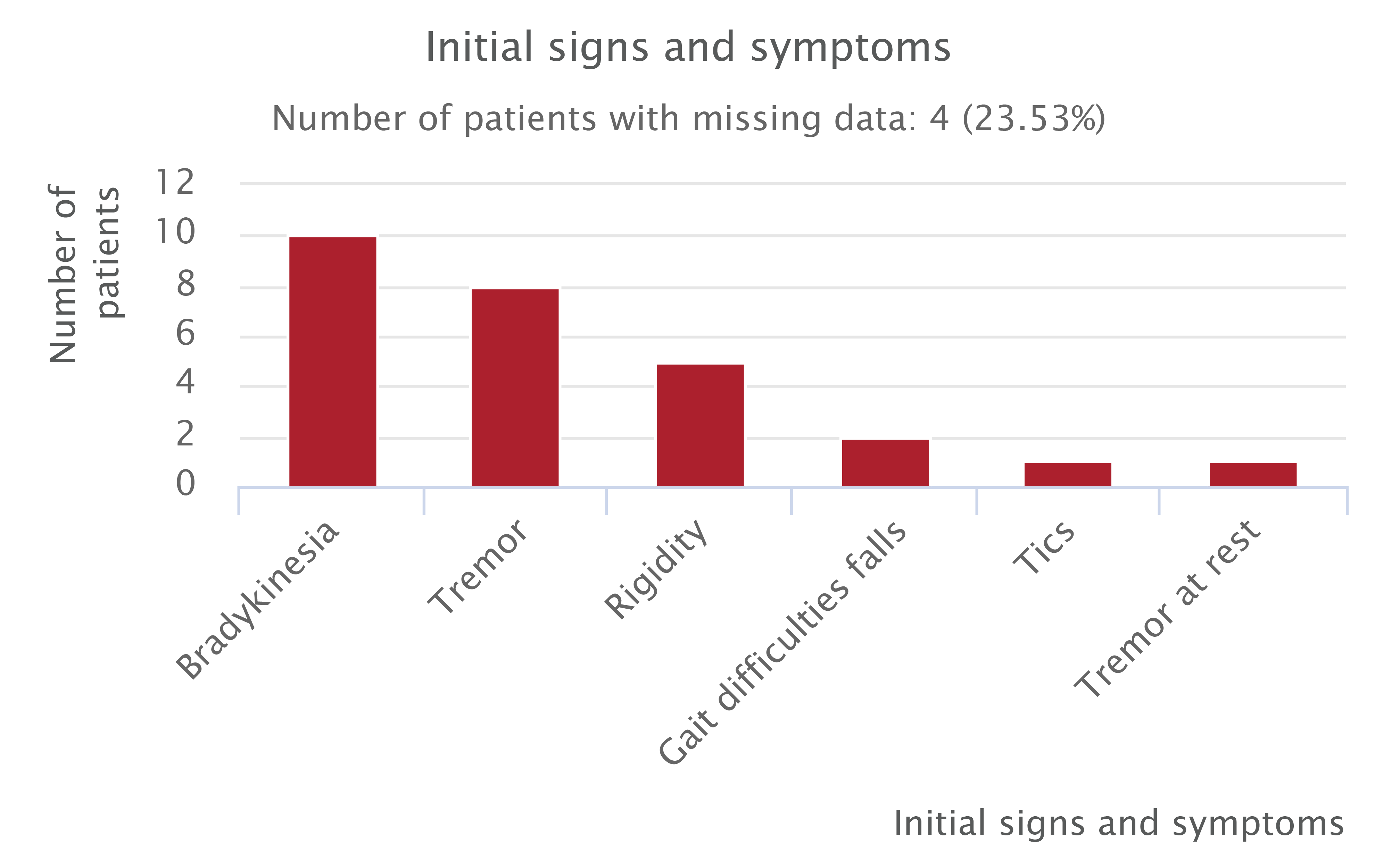 |
| **E) *VPS13C*** | |
| 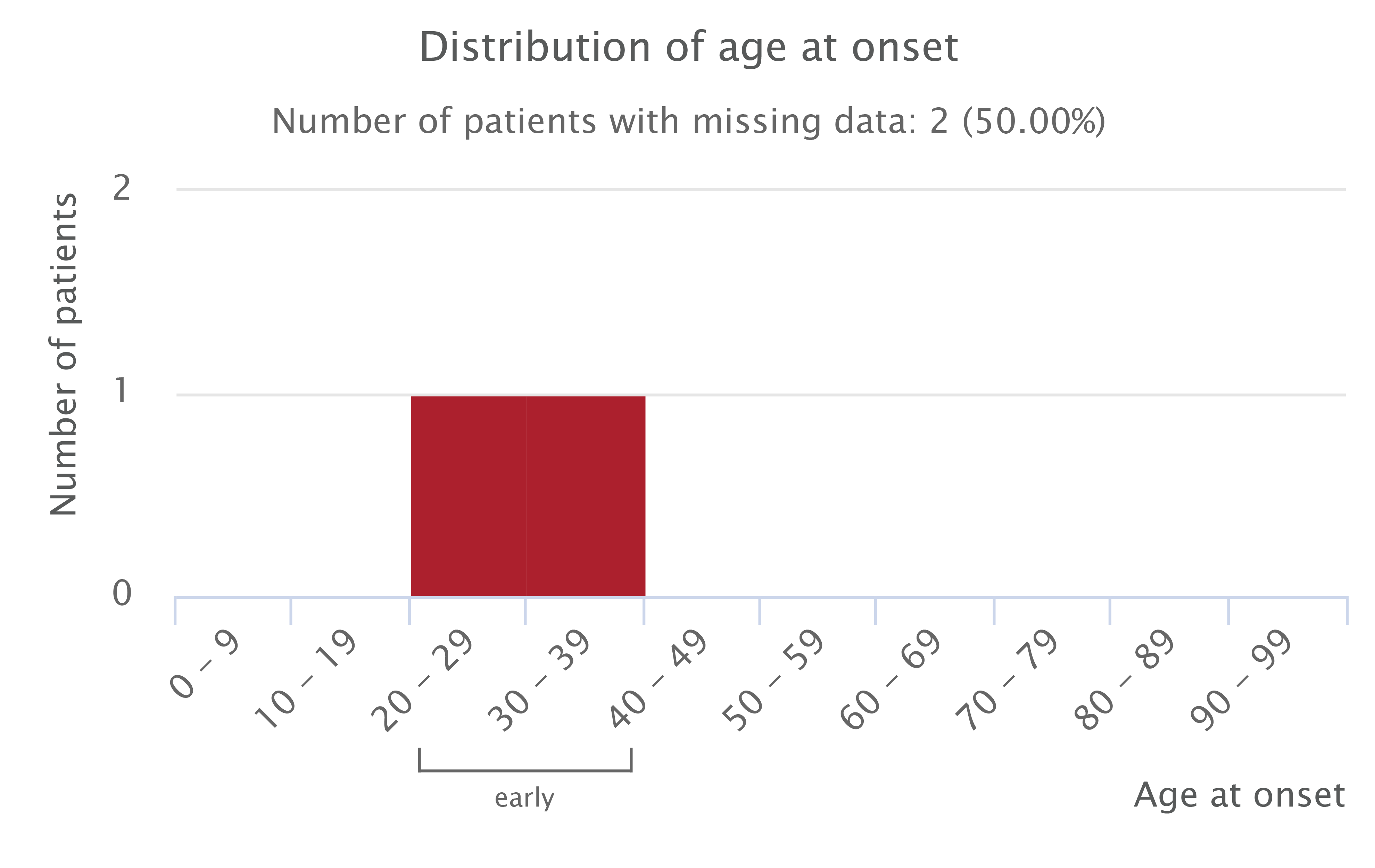 | 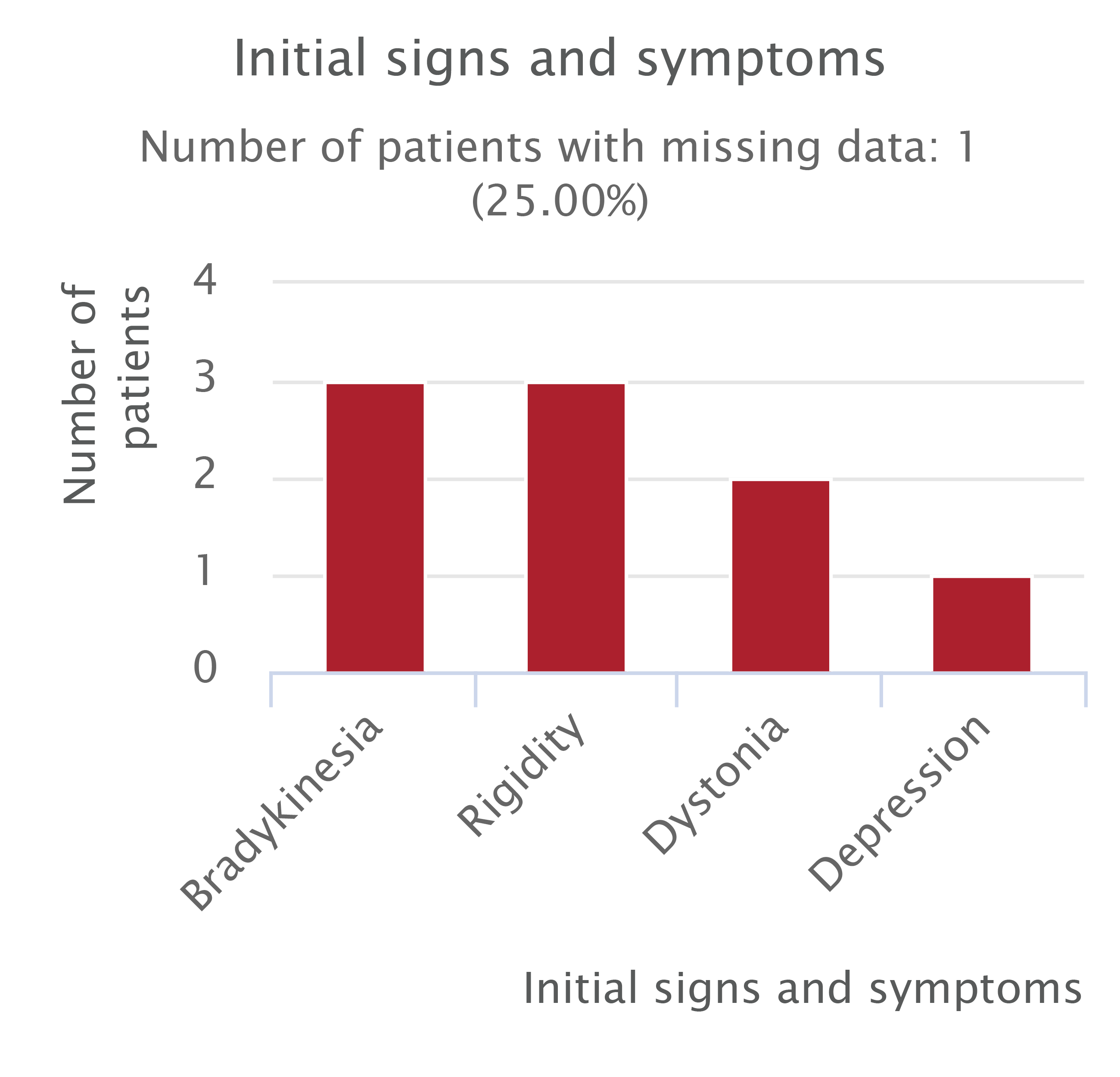 |
| **F) *DCTN1*** | |
| 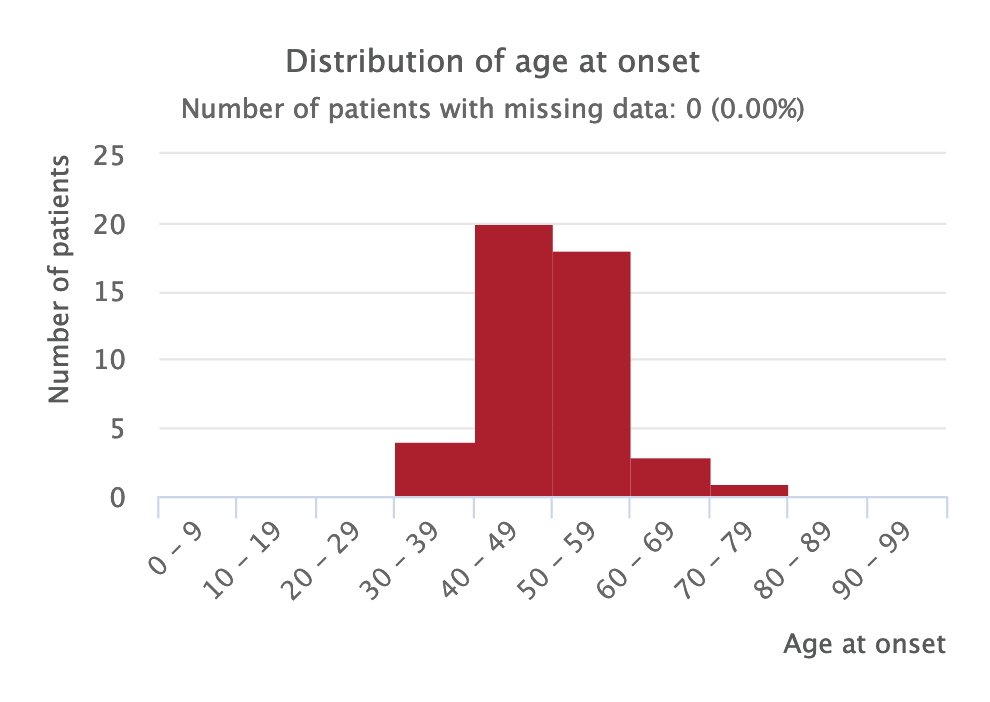 | 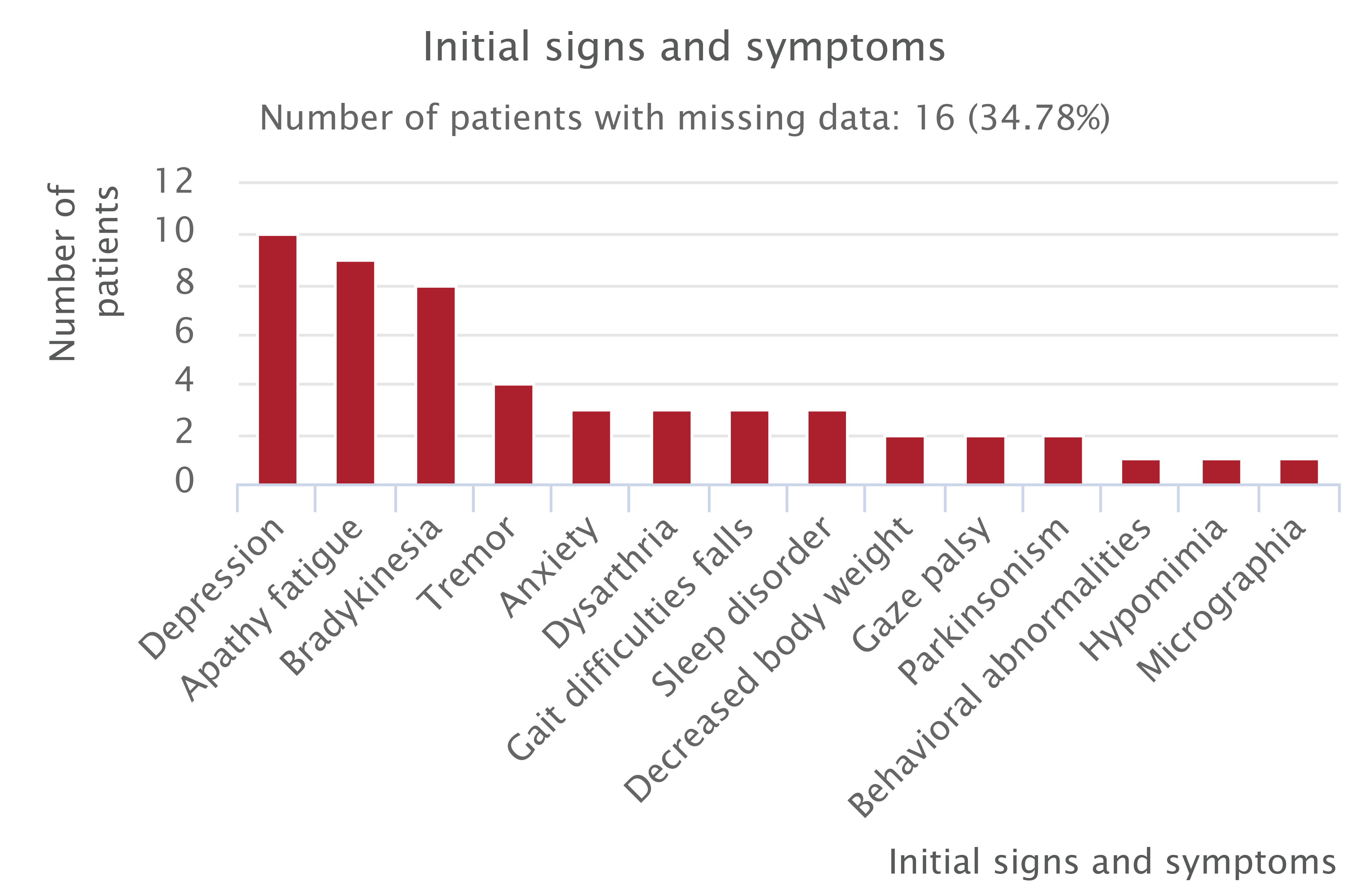 |

Figure legend. The distribution of the age at onset for patients is shown in ten years intervals.

For additional information on small fractions not specified here, use www.mdsgene.org.

**Supplementary Figure 3. Country of origin of atypical parkinsonism families**

1. ***ATP13A2***


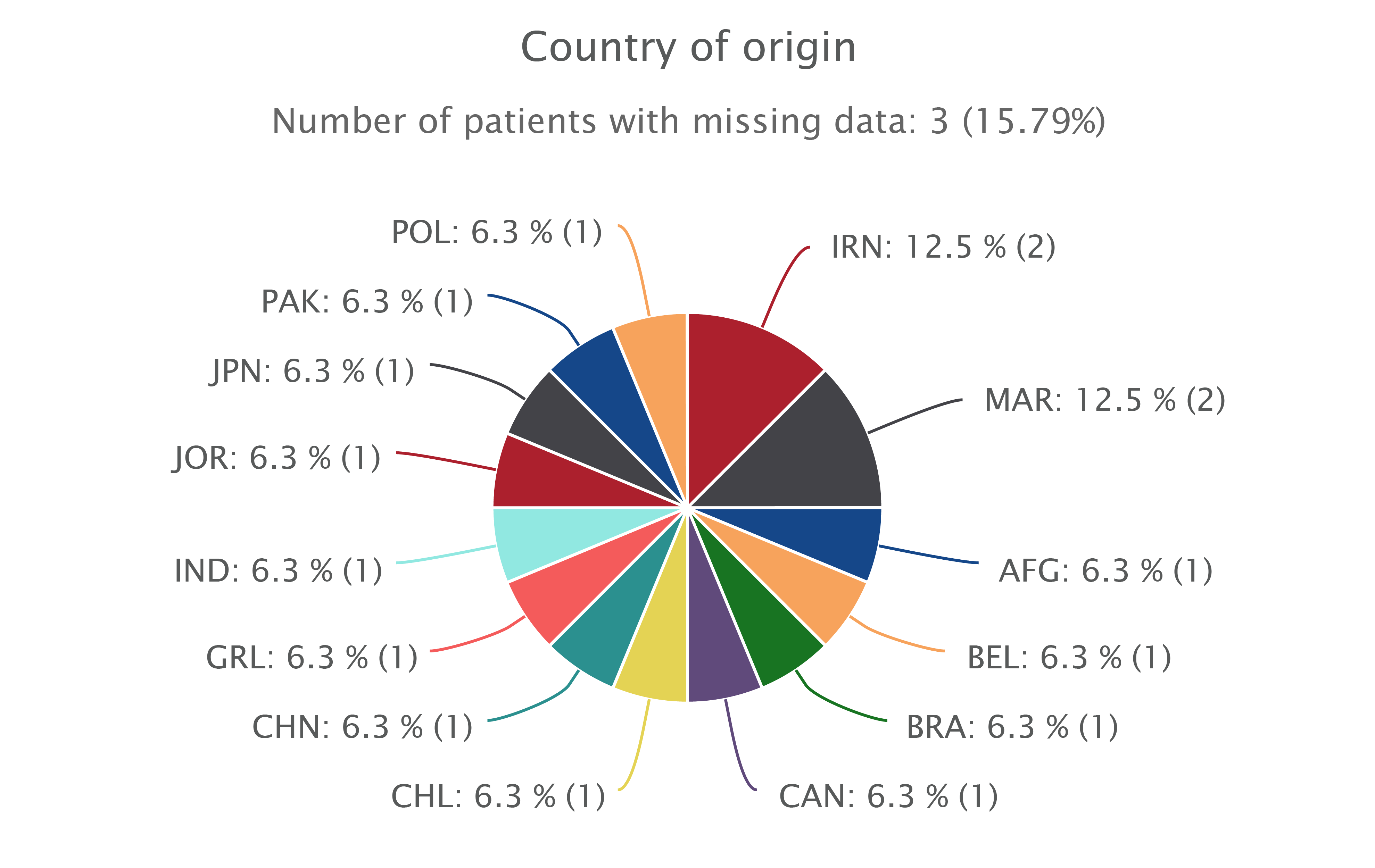


1. ***DNAJC6***


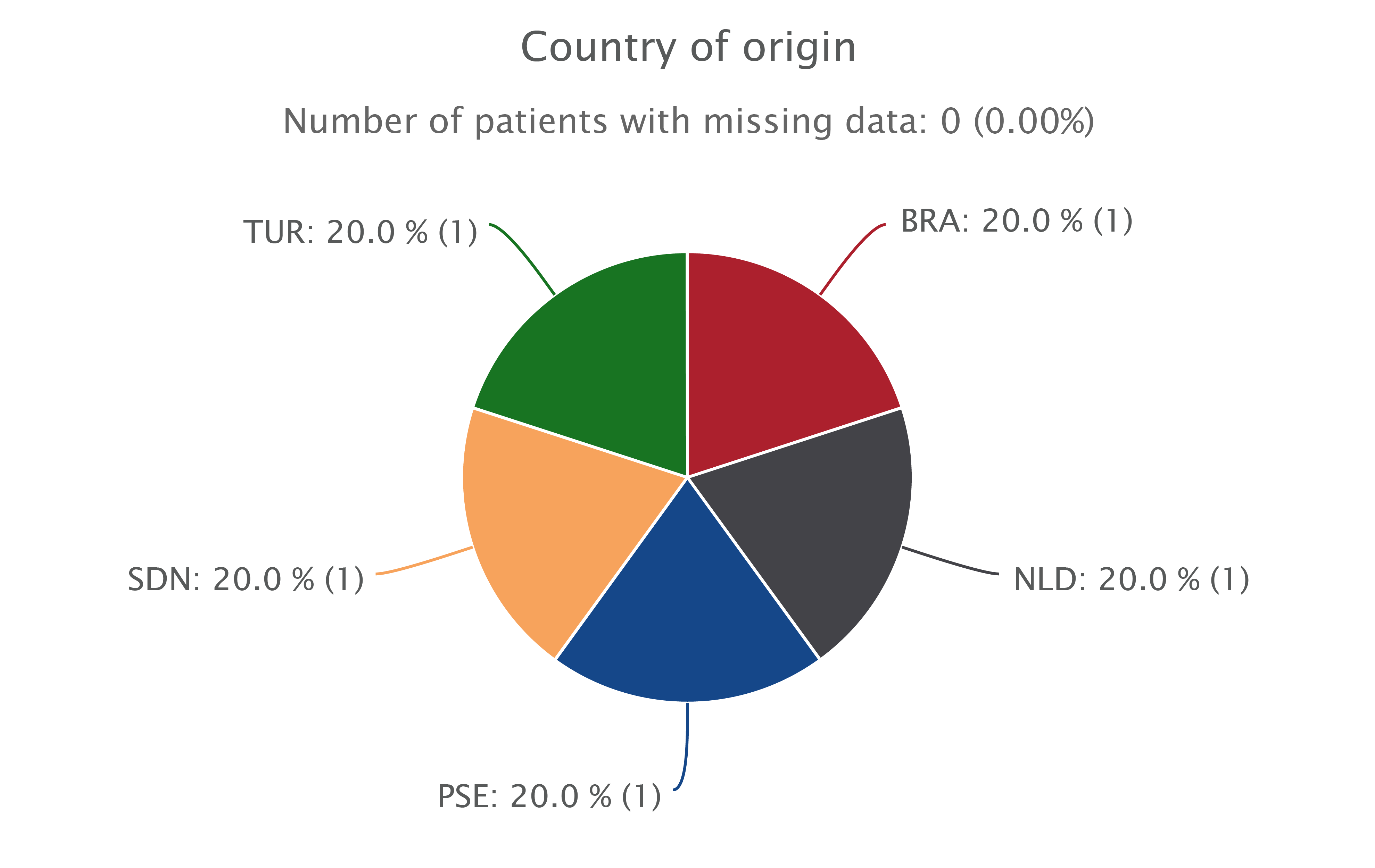


1. ***FBXO7***


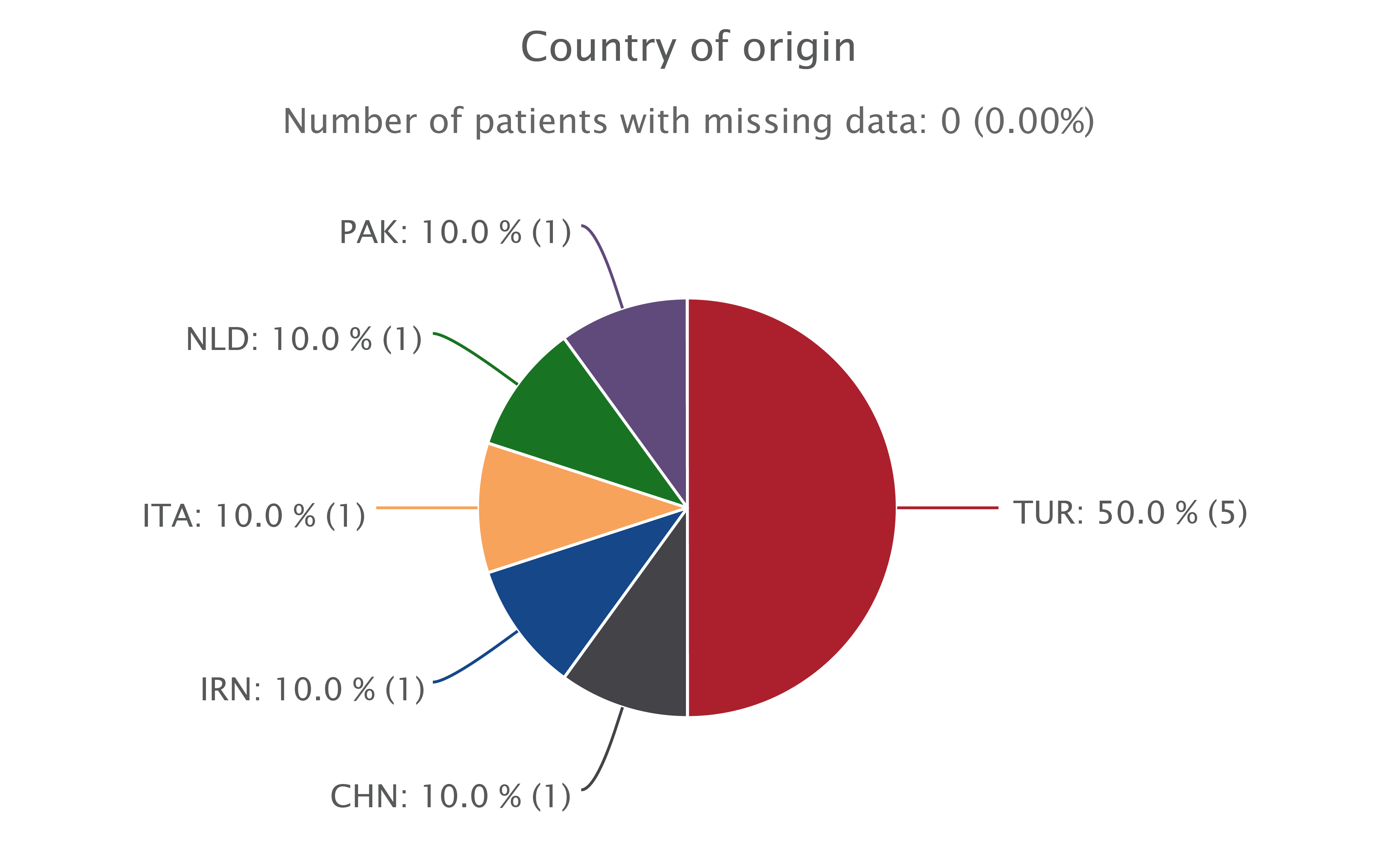


1. ***SYNJ1***


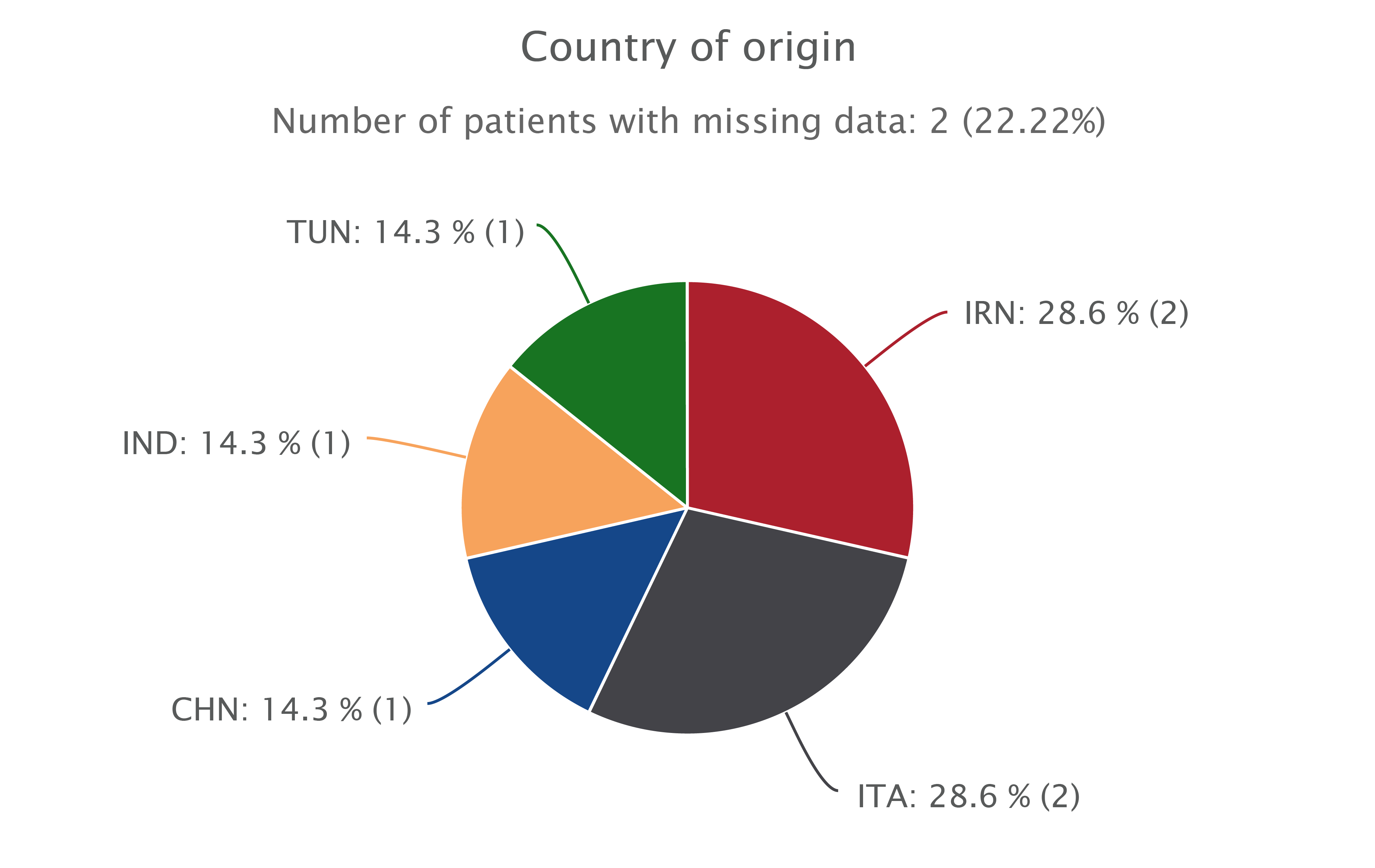


**E) *VPS13C***


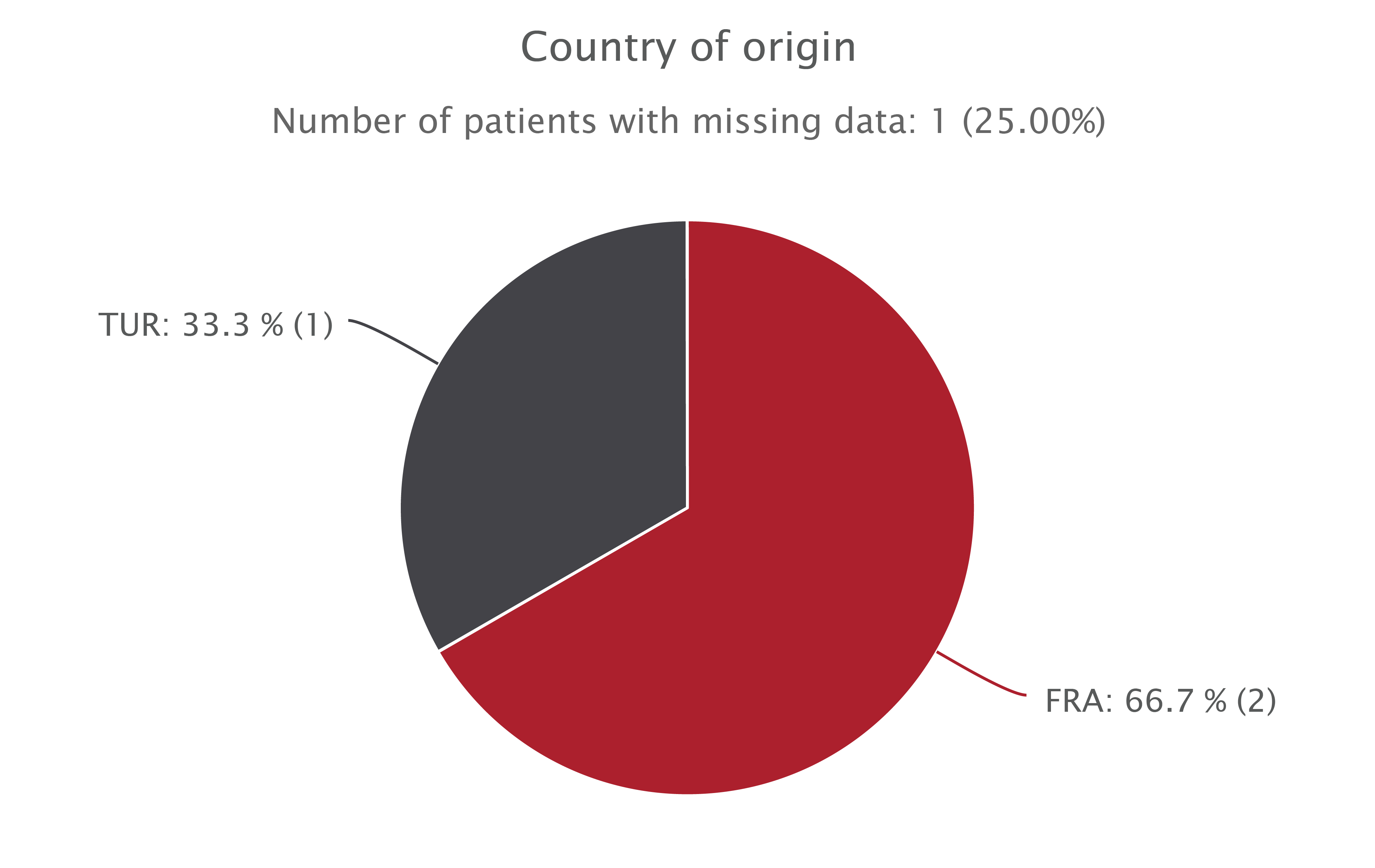


**F) *DCTN1***


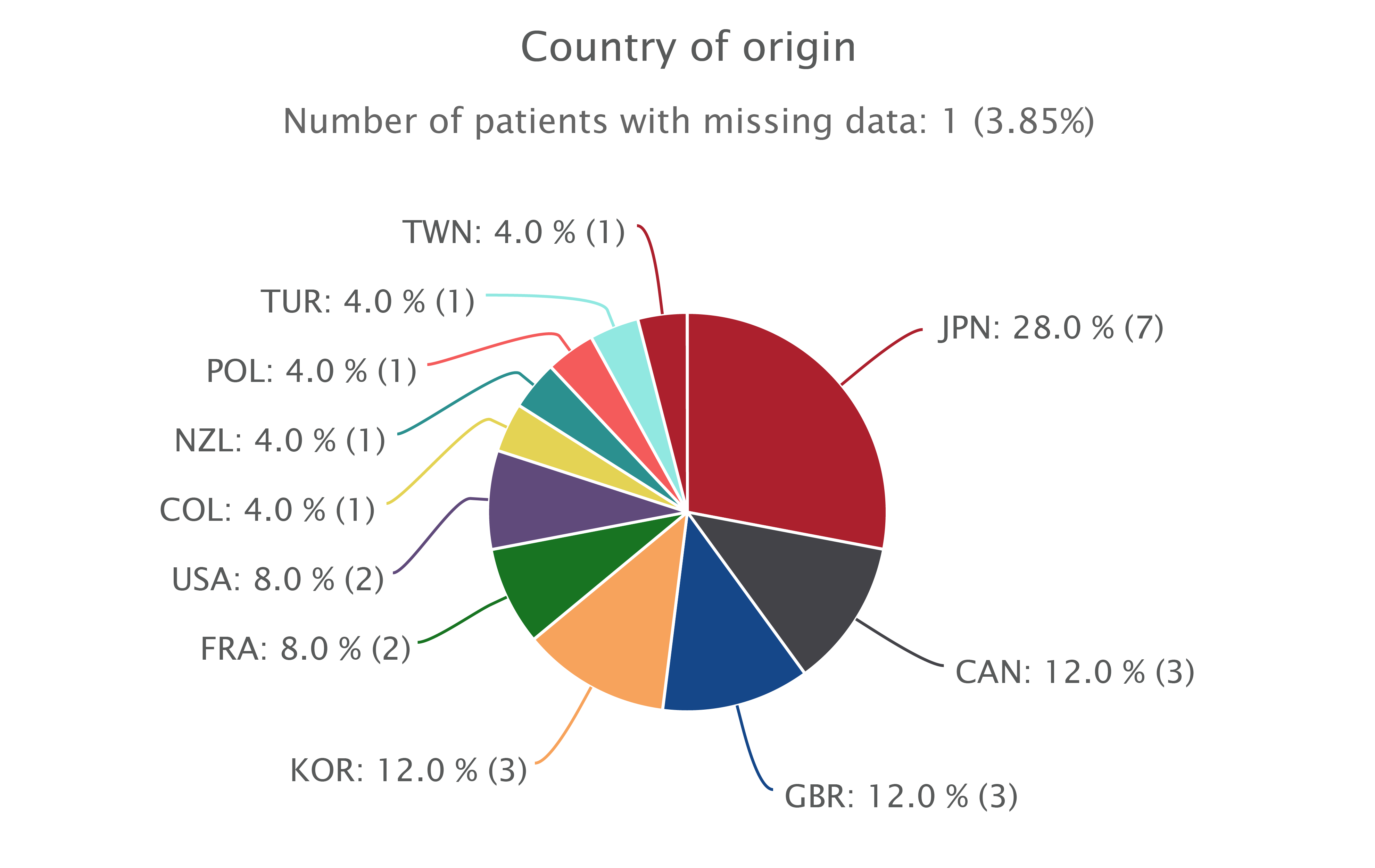


Figure legend. Countries are represented by the three-letter Code ISO 3166-1 alpha-3 published by the International Organization for Standardization. For additional information on small fractions not specified here, please see www.mdsgene.org

**Supplementary Figure 4. Frequency of the most common mutations and functional consequences of all 56 different mutations reported in atypical parkinsonism index patients**

***A) ATP13A2*:**


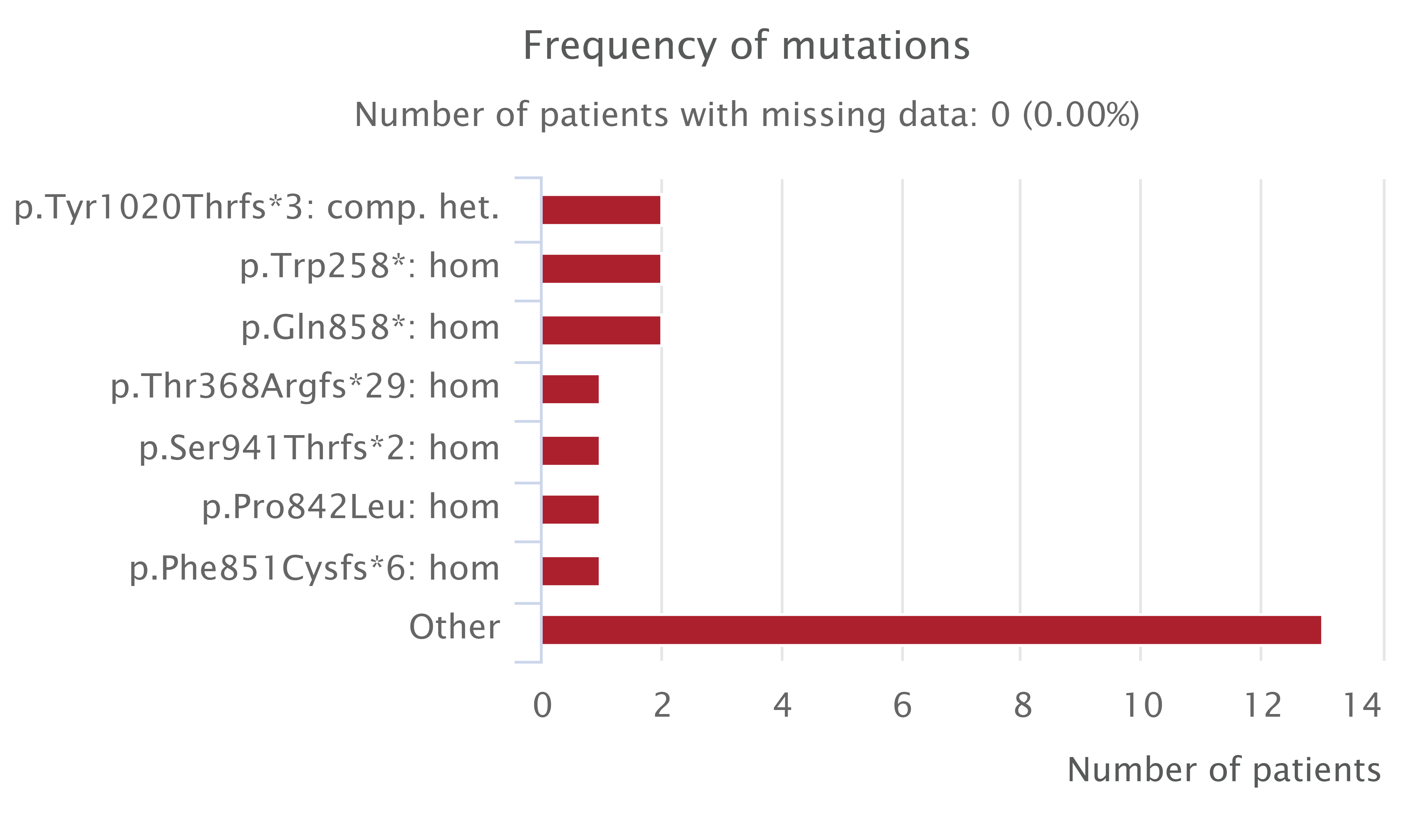


**B) *DNAJC6*:**


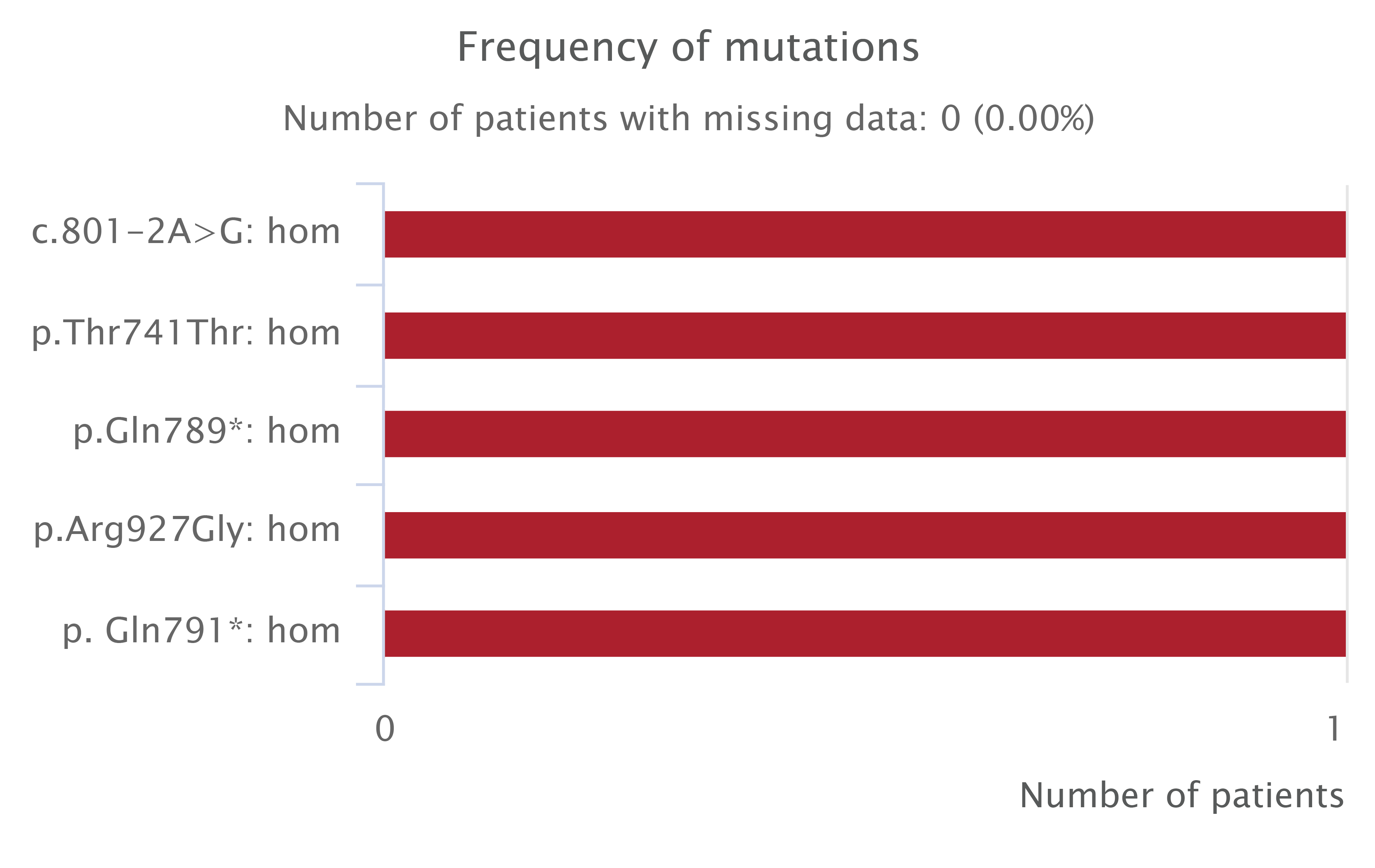


**C) *FBXO7*:**


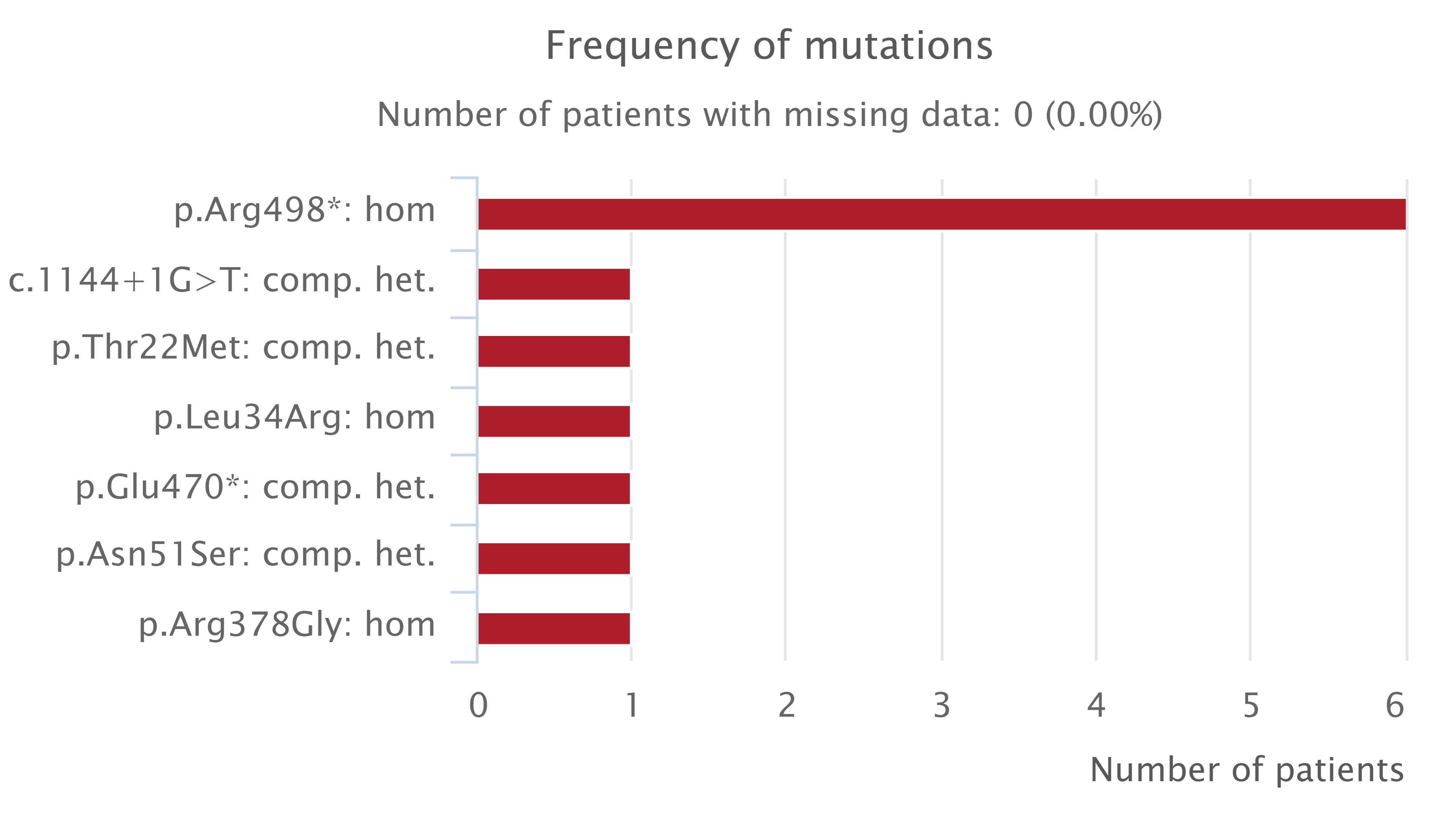


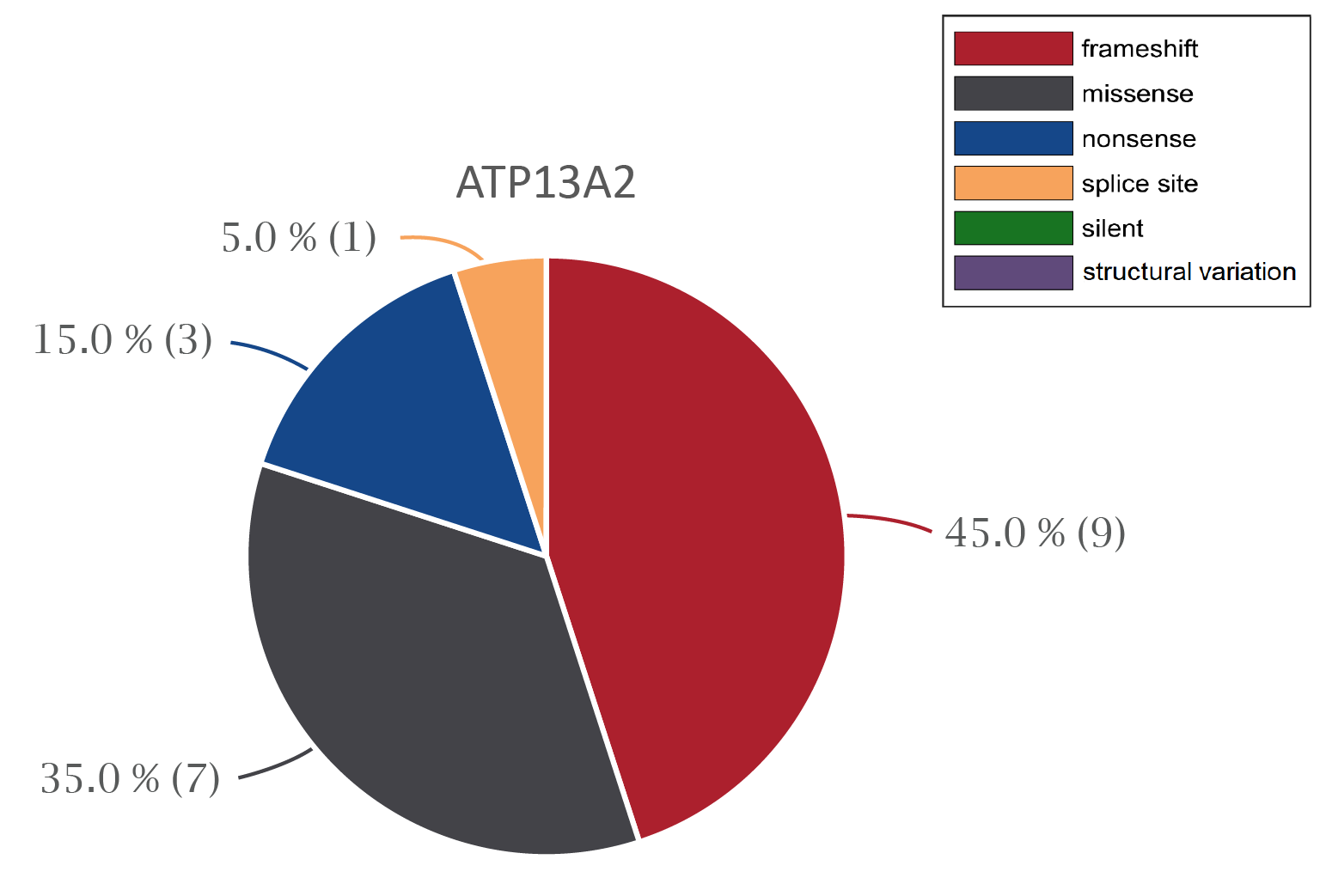


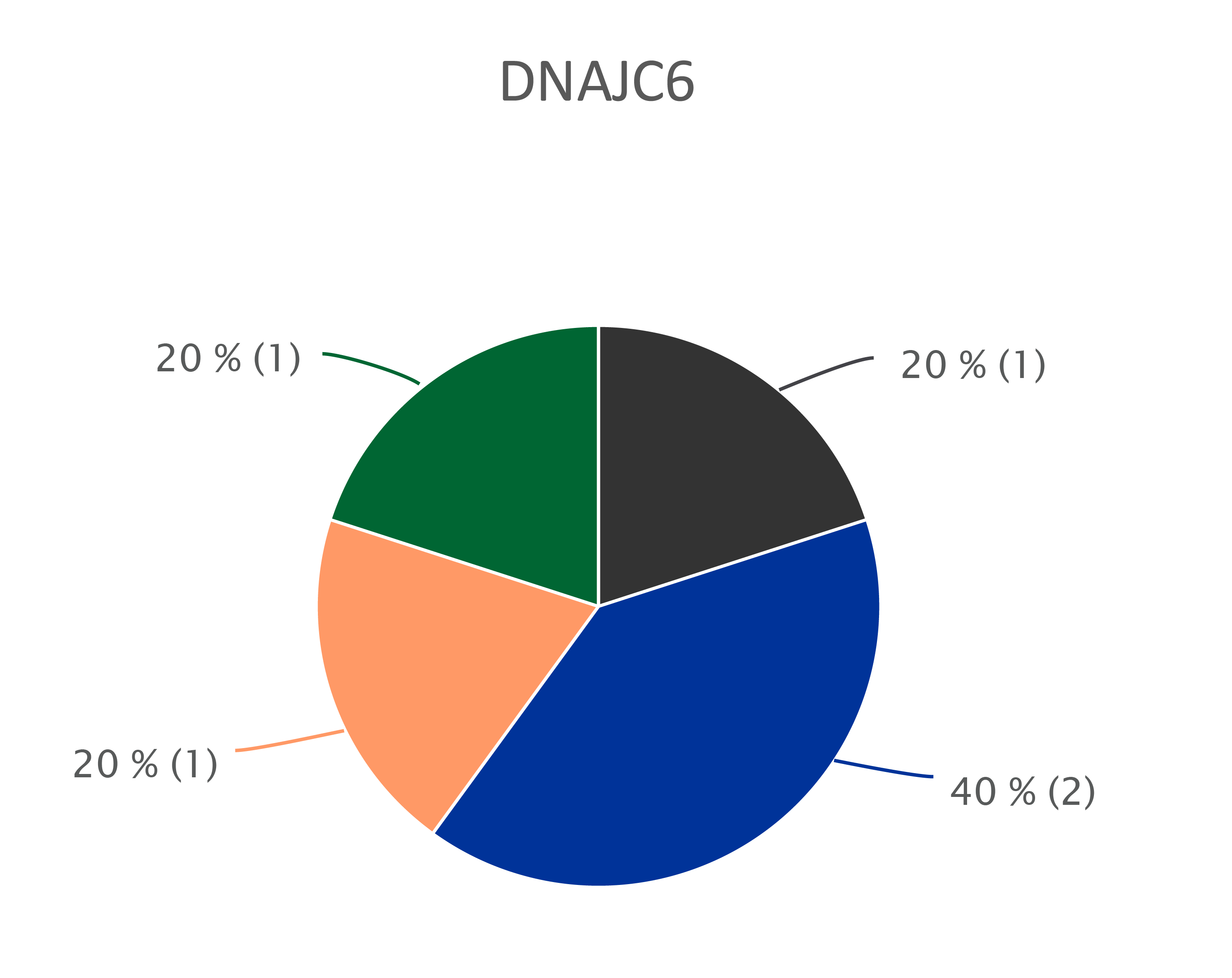


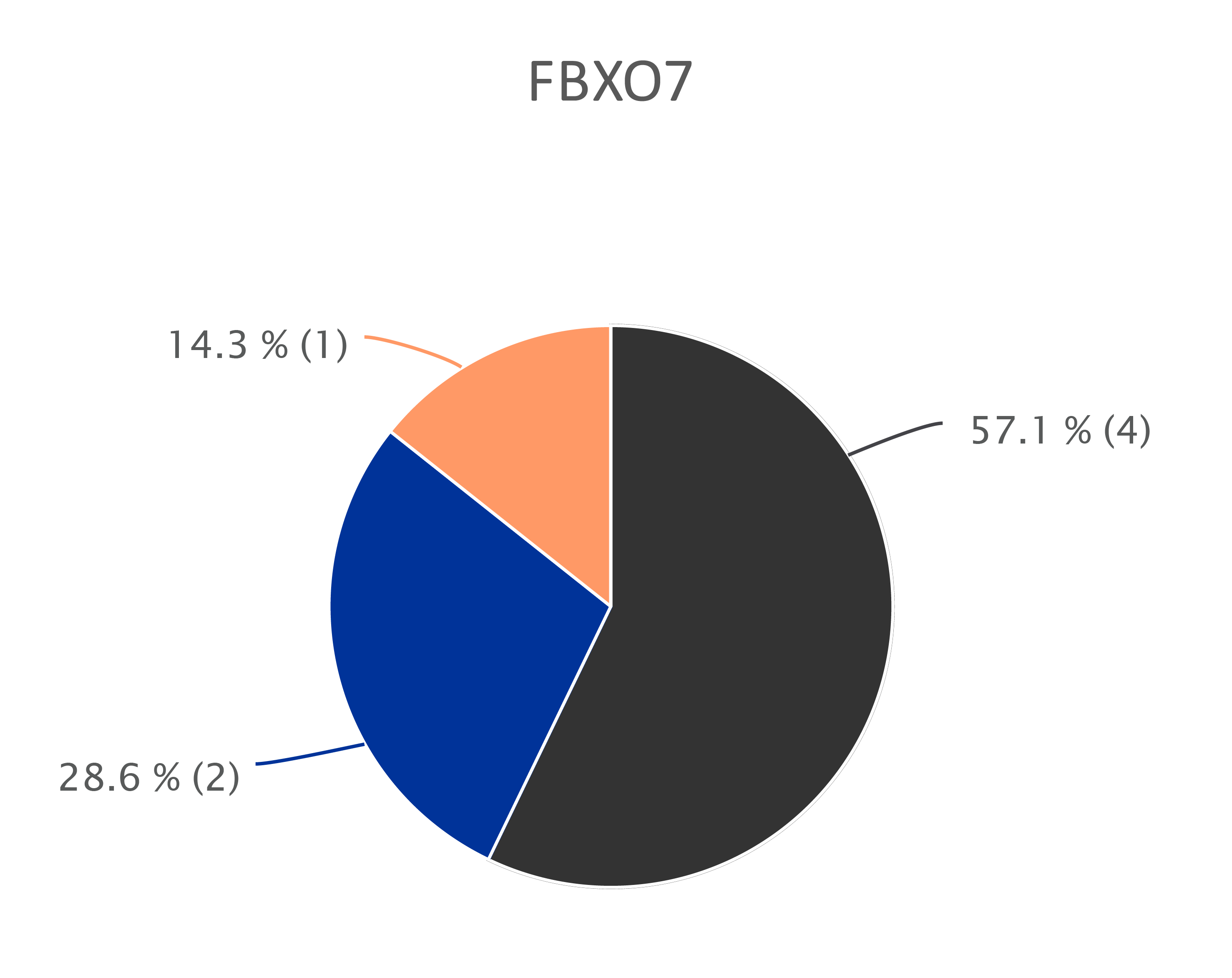


1. ***SYNJ1*:**


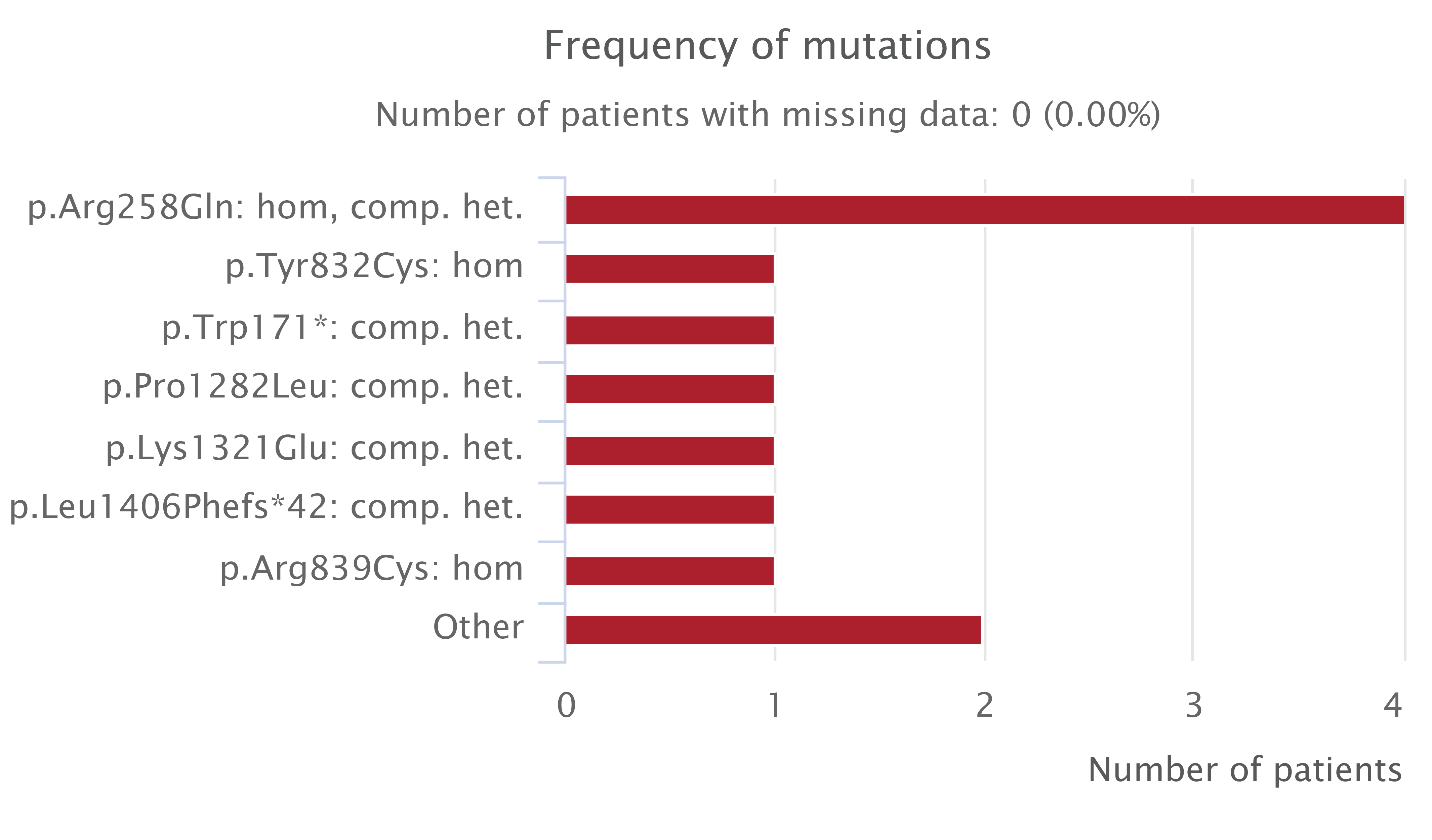


1. ***VPS13C*:**


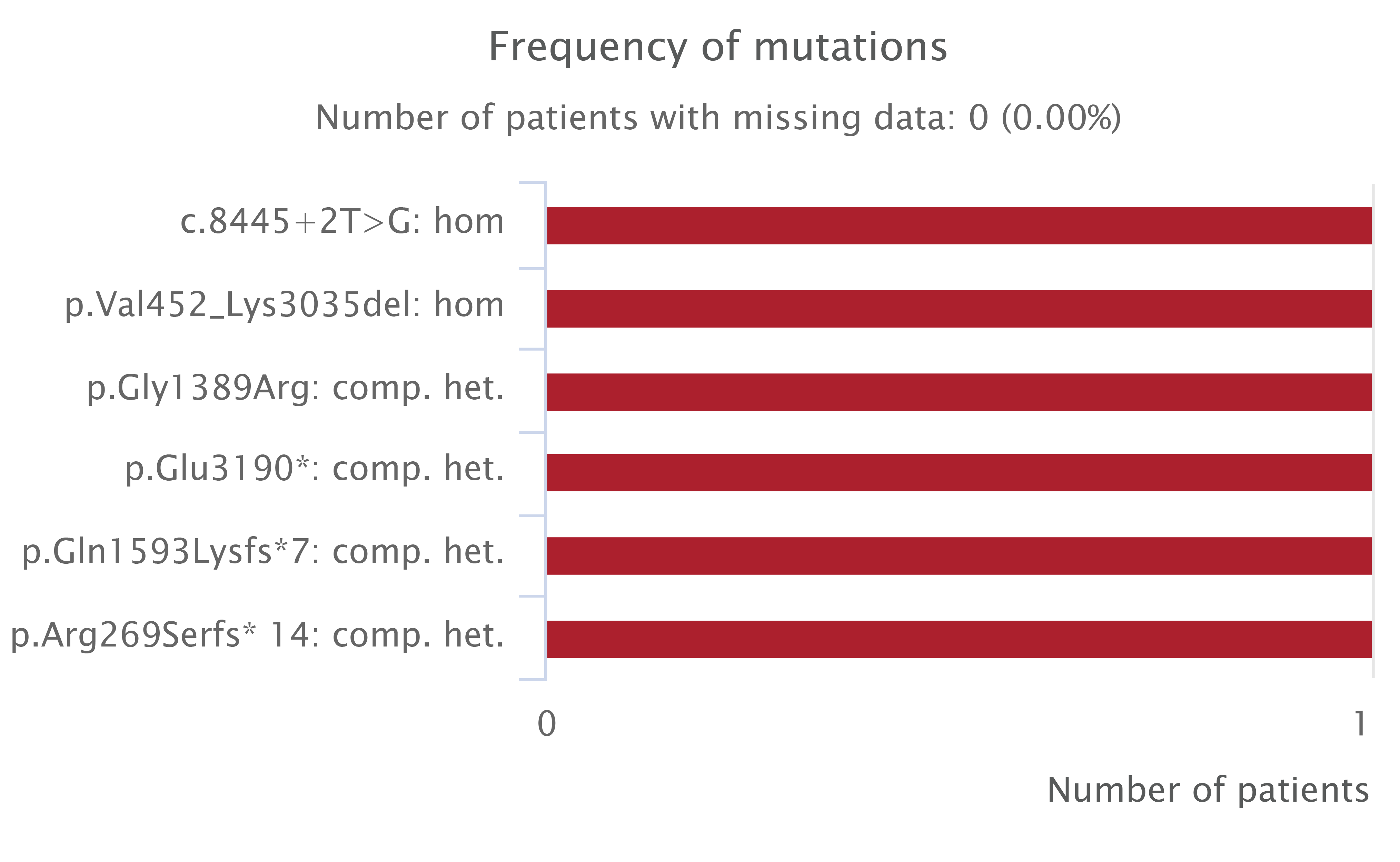


1. ***DCTN1:***


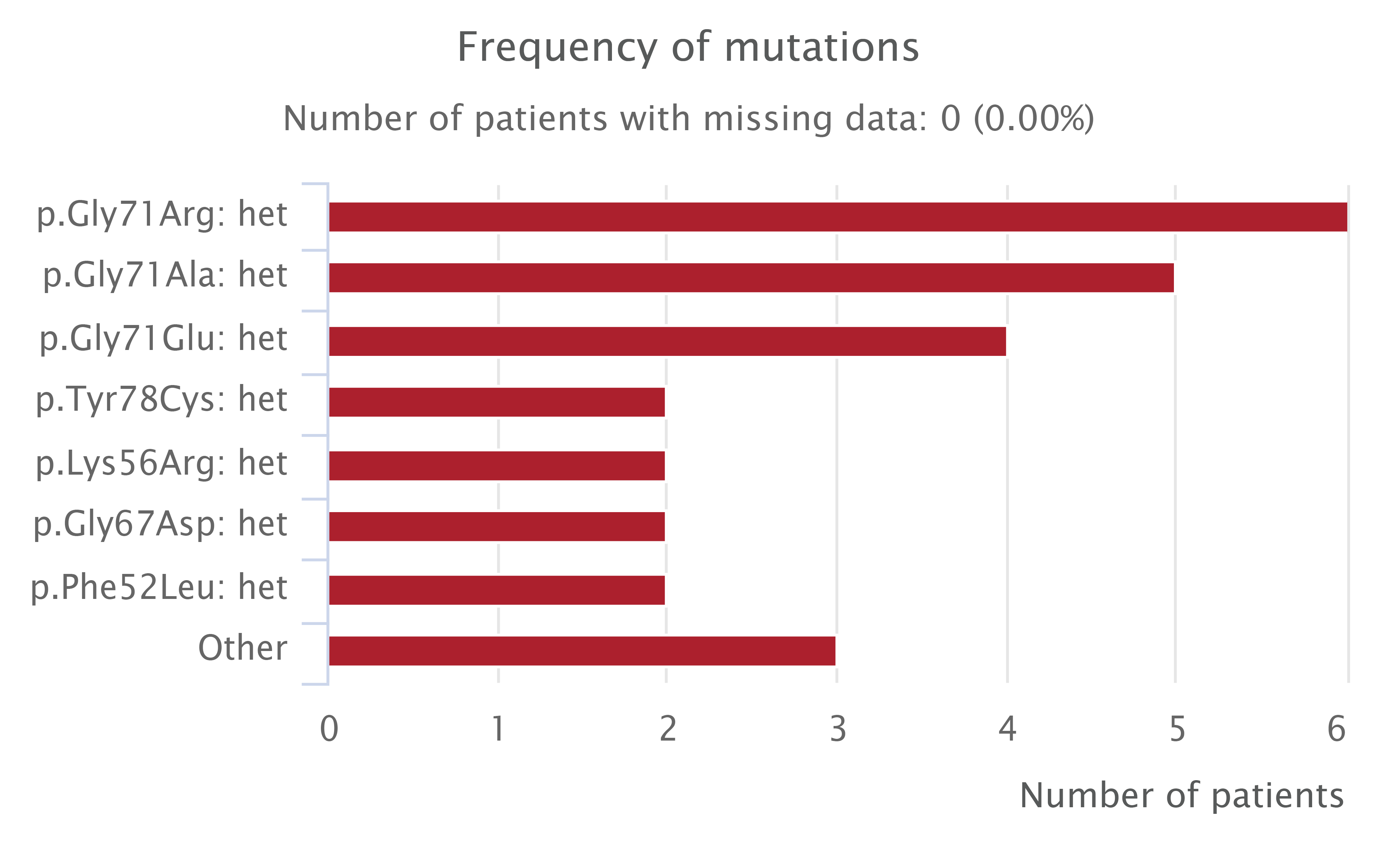


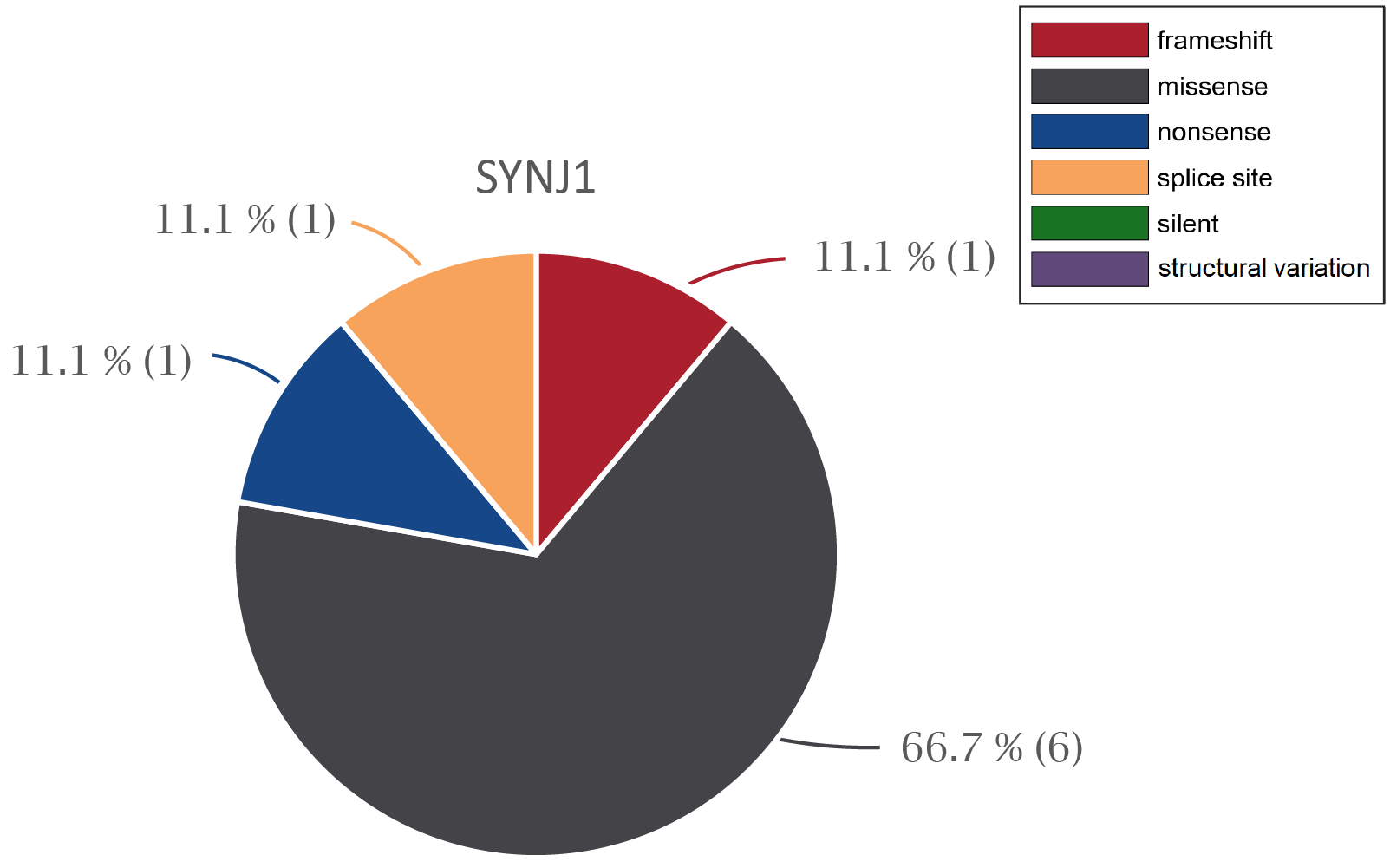


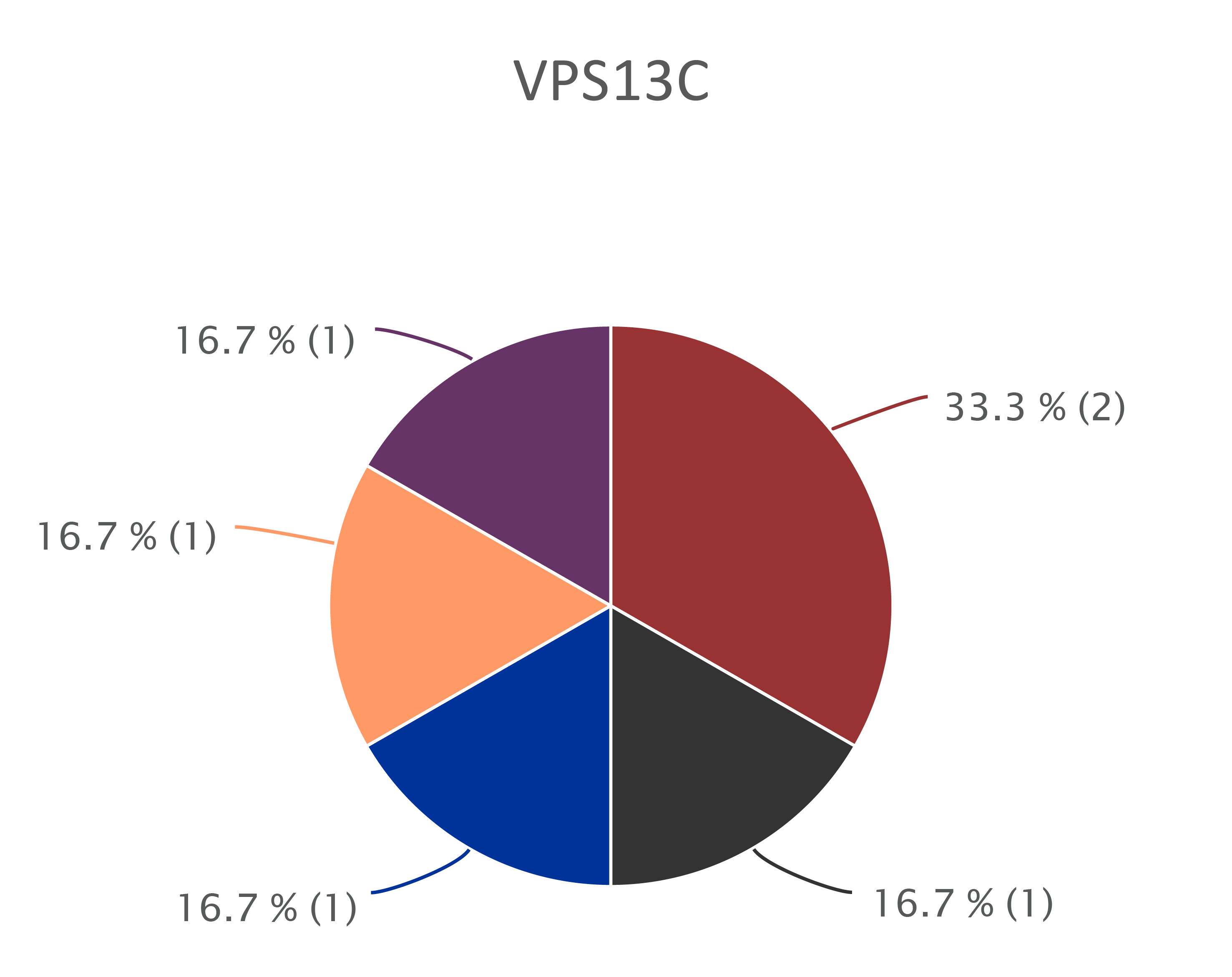


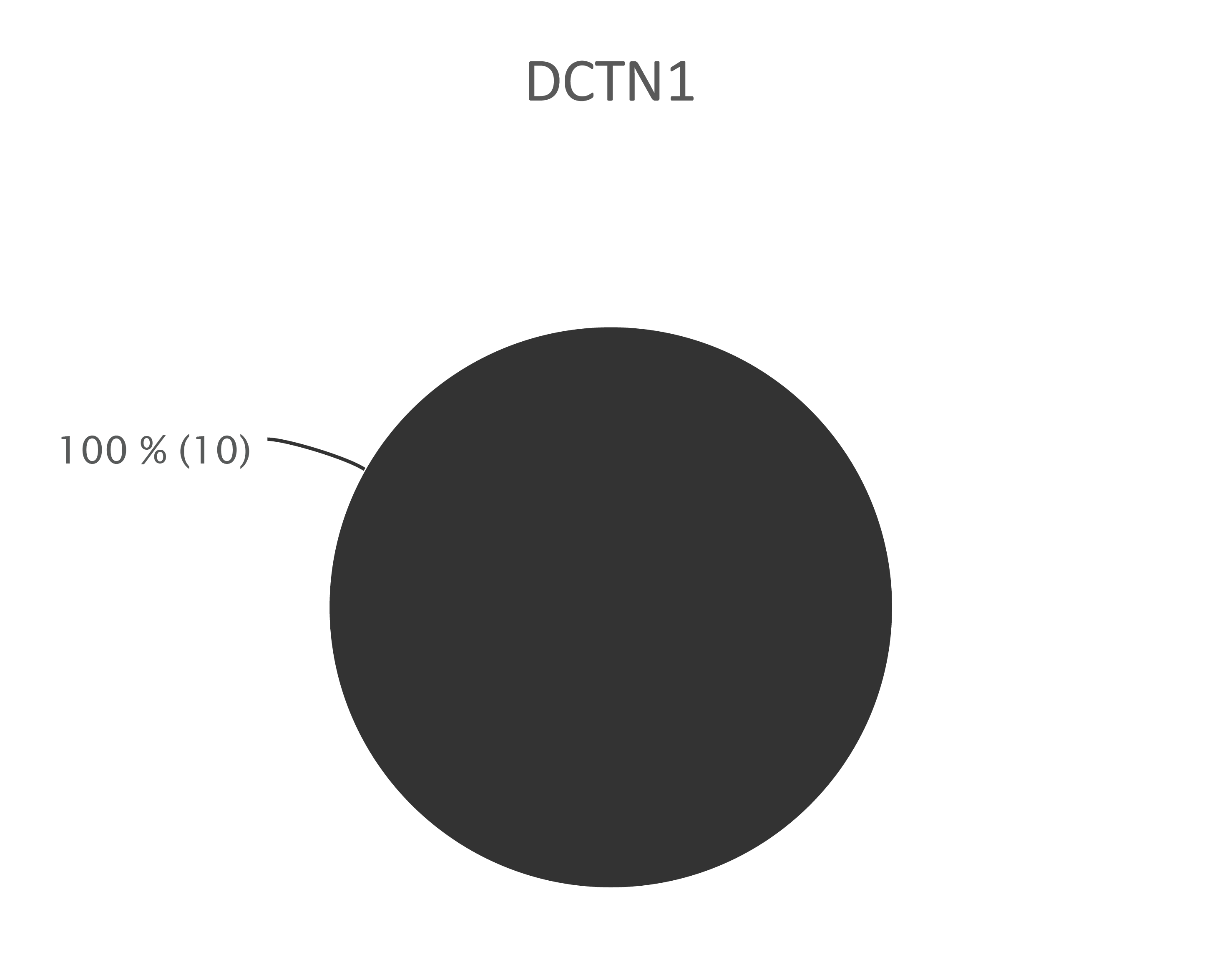


Figure legend. For the assessment of functional consequences, every mutation was counted only once, regardless of the actual frequency in mutation carriers.

**Supplementary Figure 5. Response quantification for Levodopa in monogenic atypical parkinsonism patients**

**A) *ATP13A2*:**


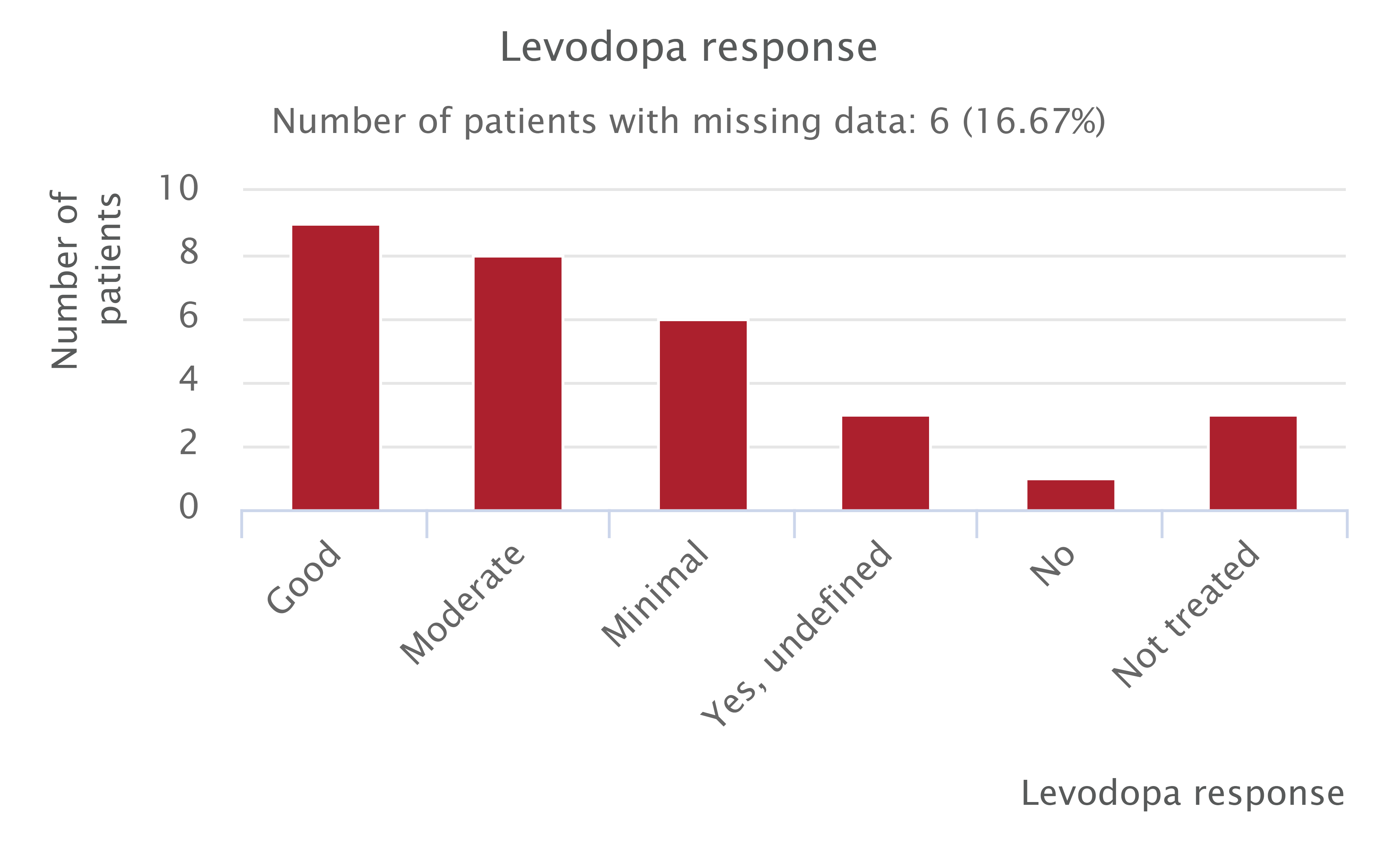


**B) *DNAJC6*:**


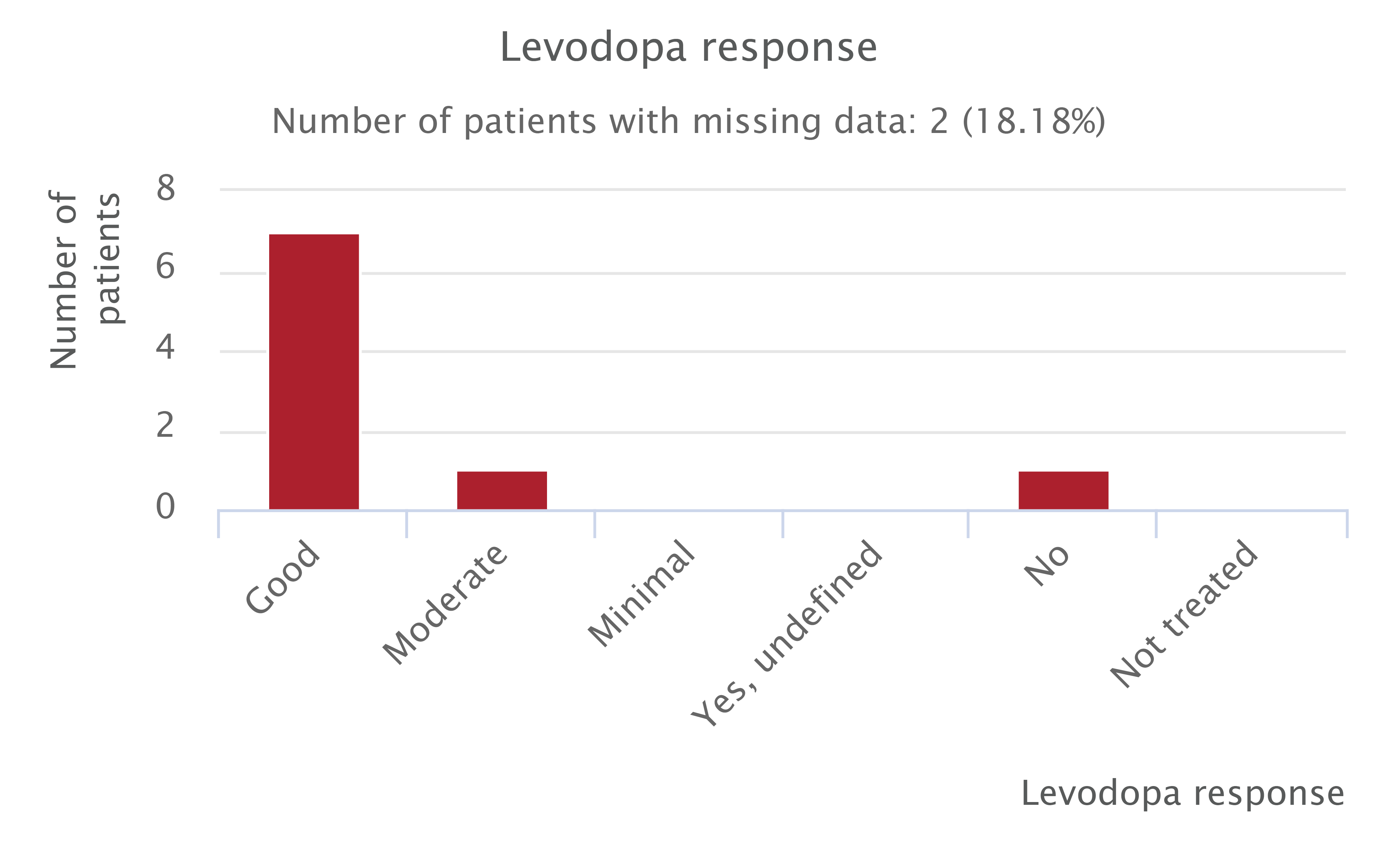


**C) *FBXO7*:**


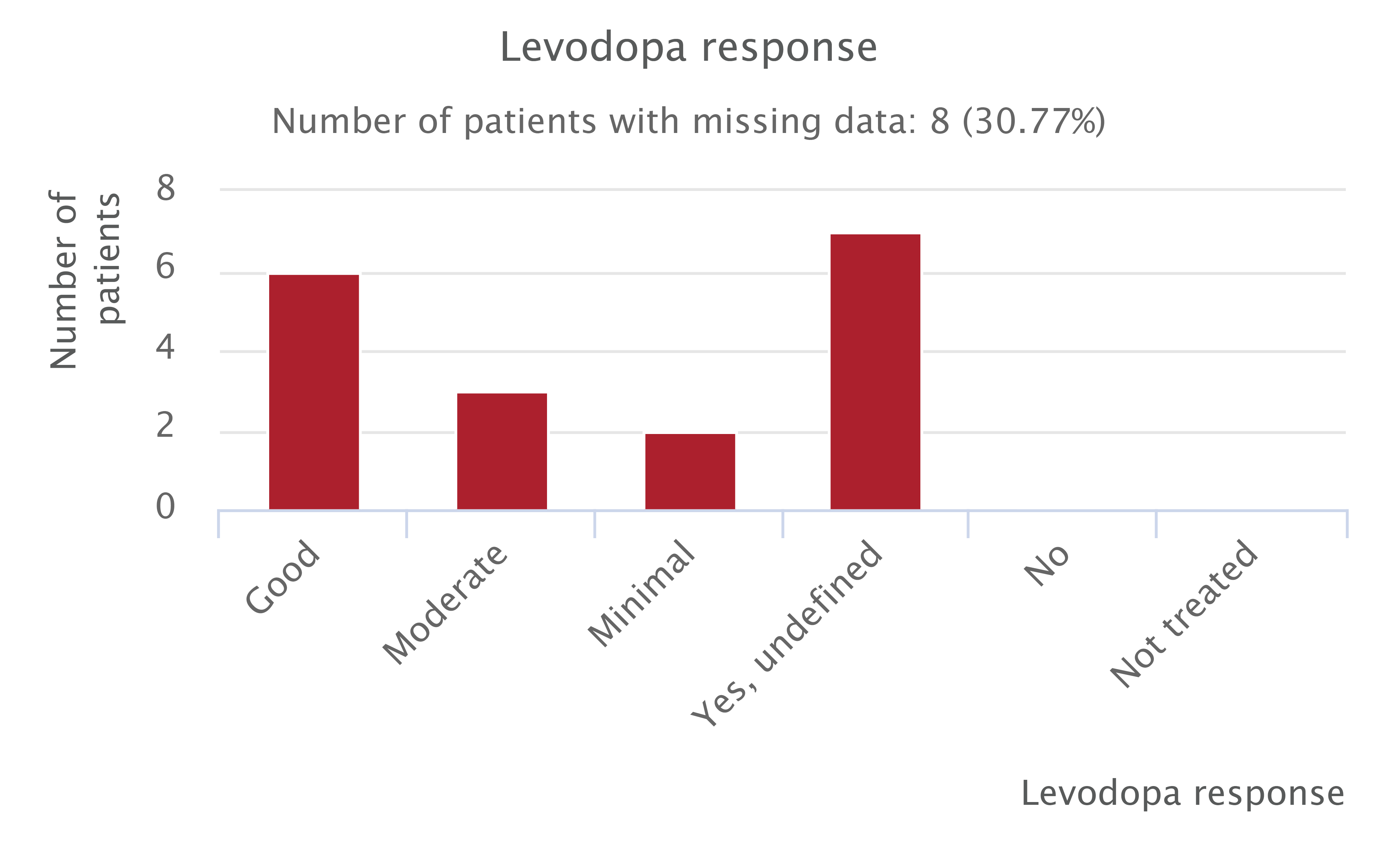


**D) *SYNJ1*:**


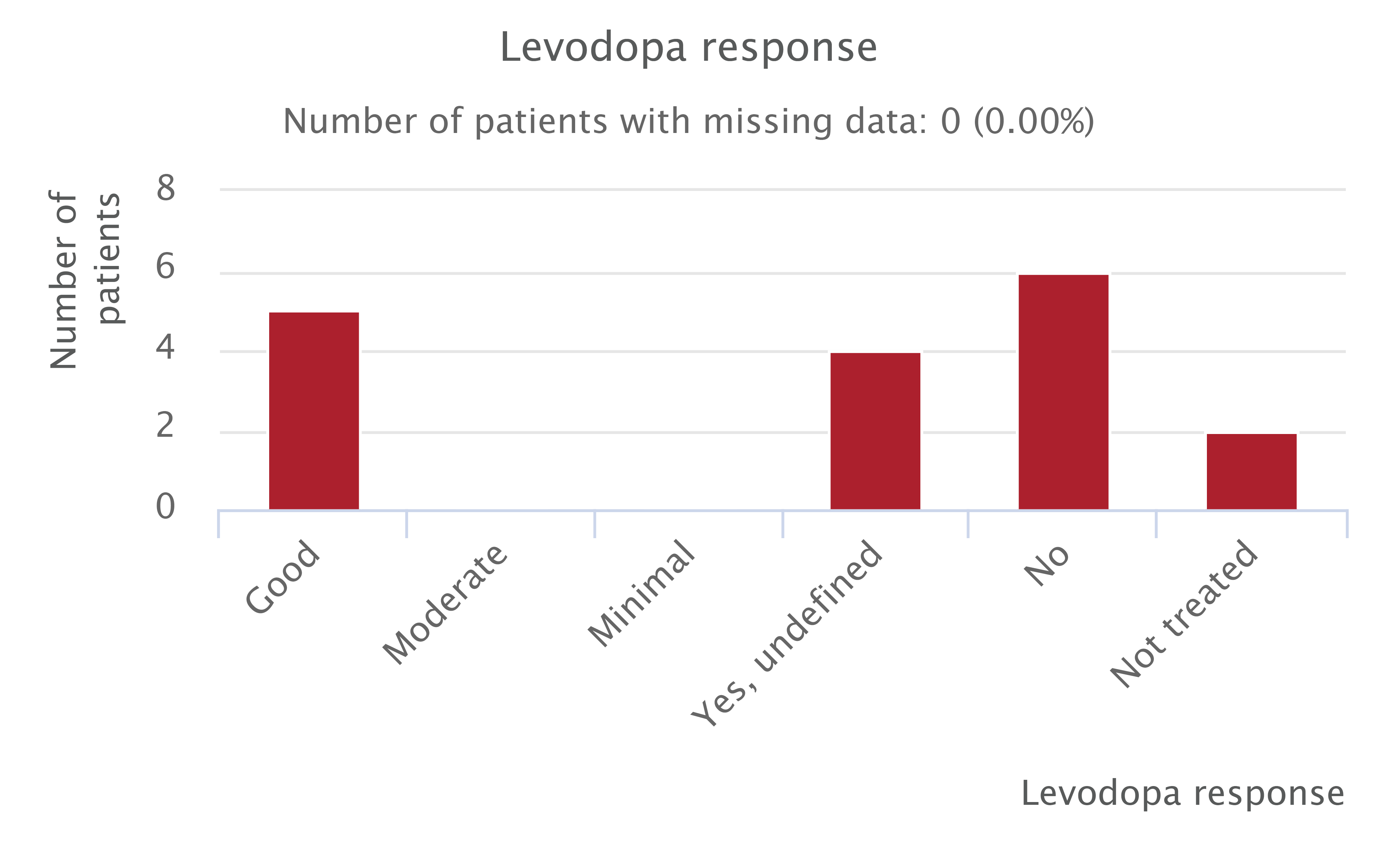


**E) *VPS13C***


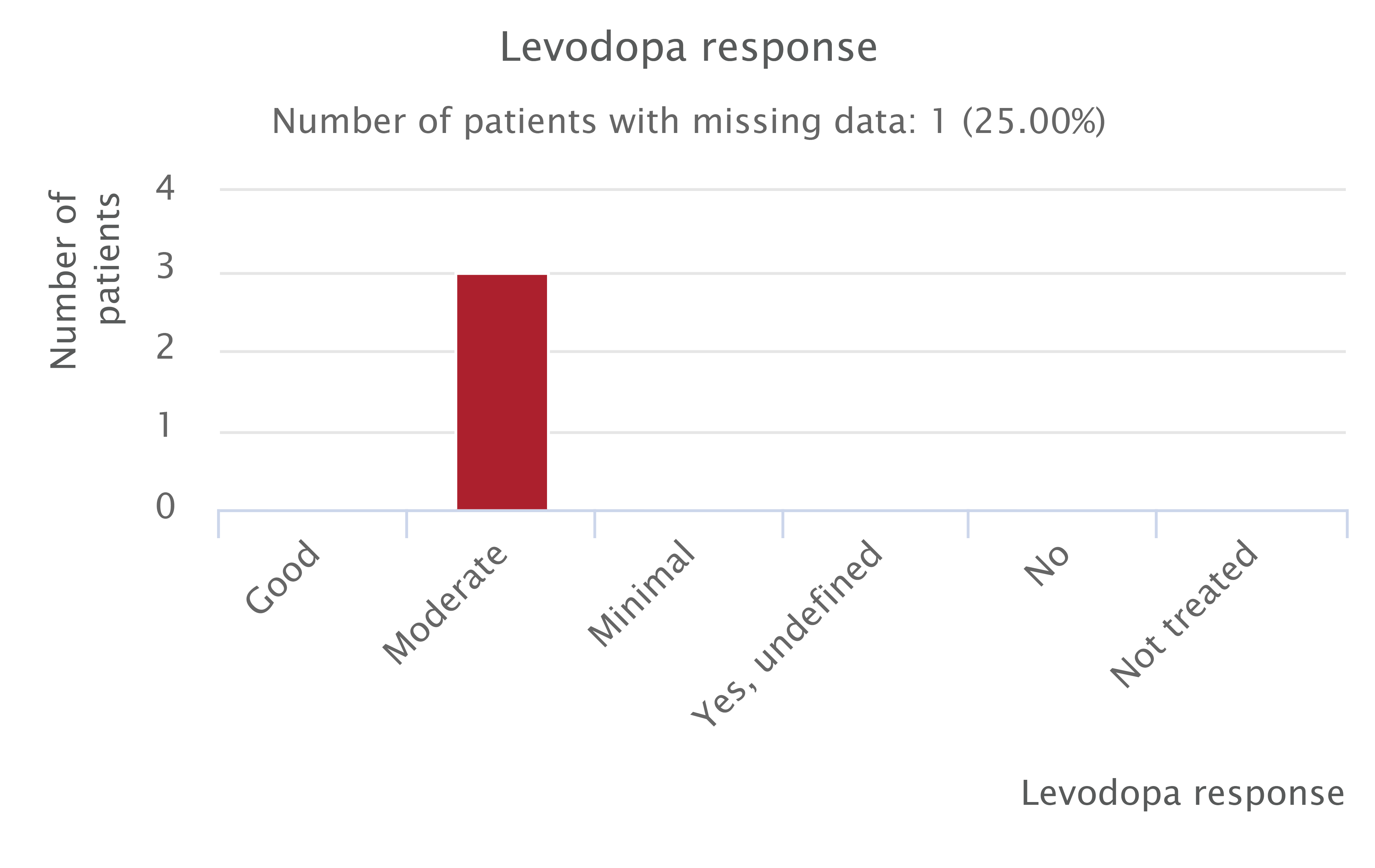


**F) *DCTN1*:**


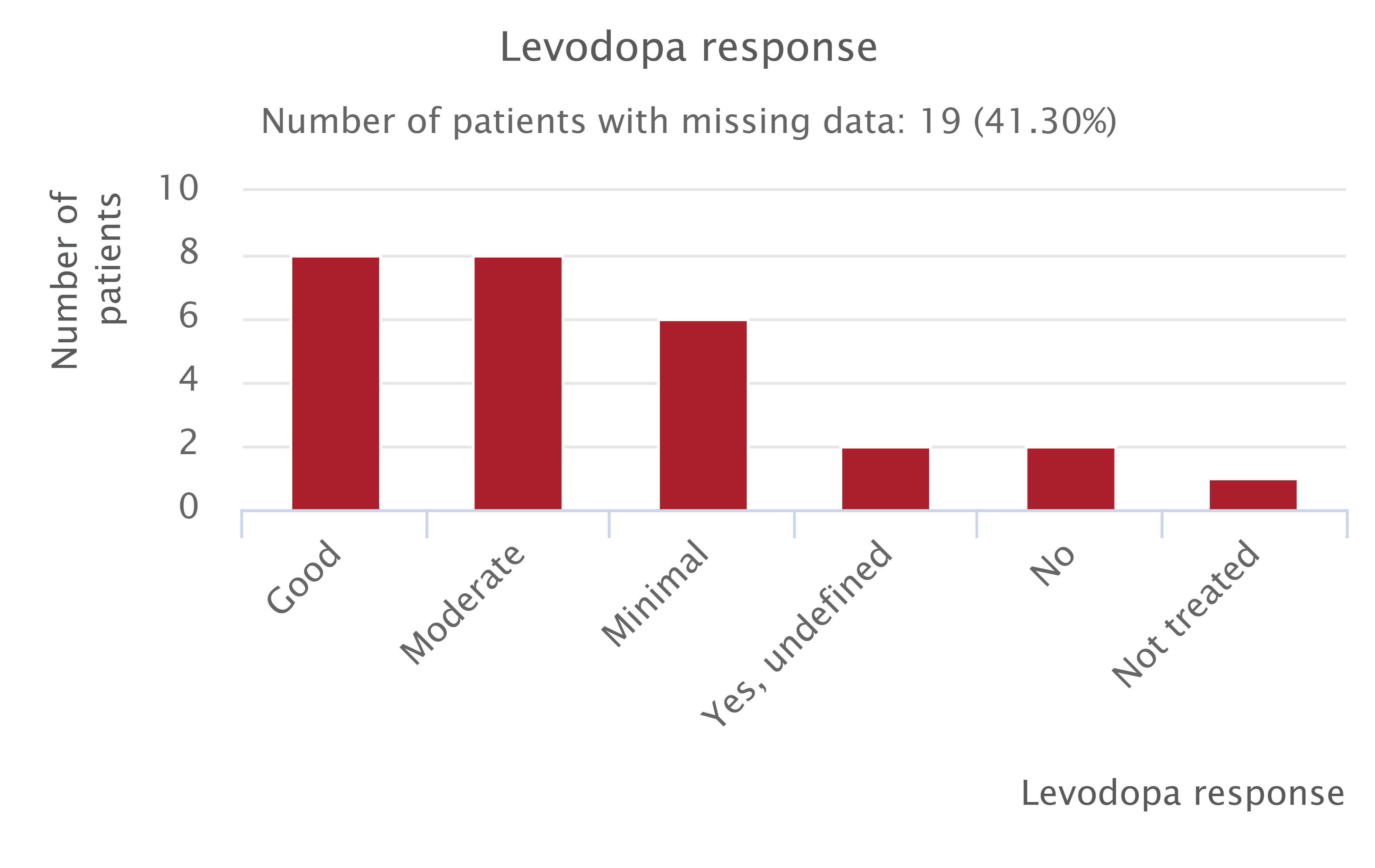


**Supplementary Figure 6. A) Reported signs and symptoms in patients with *DNAJC6* mutations B) Schematic representation of the *DNAJC6* gene (upper scheme) and protein (lower scheme) and mutations listed in MDSGene**

**A)**


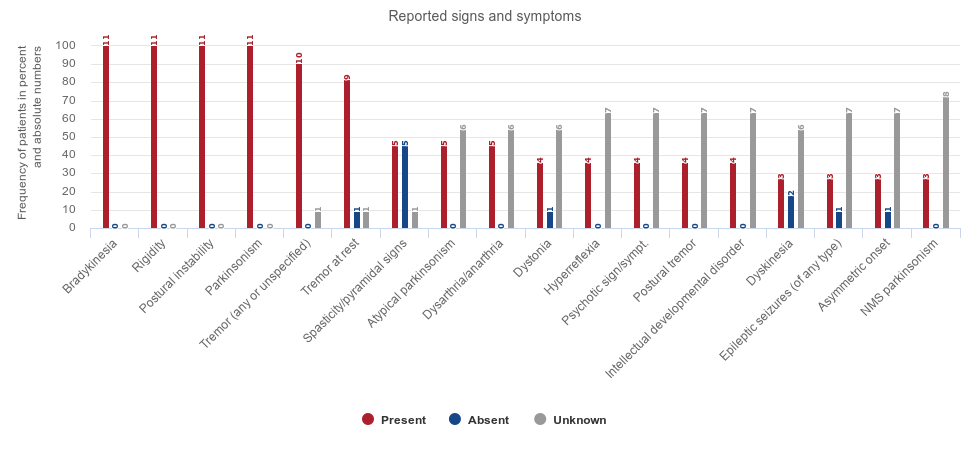


**B)**


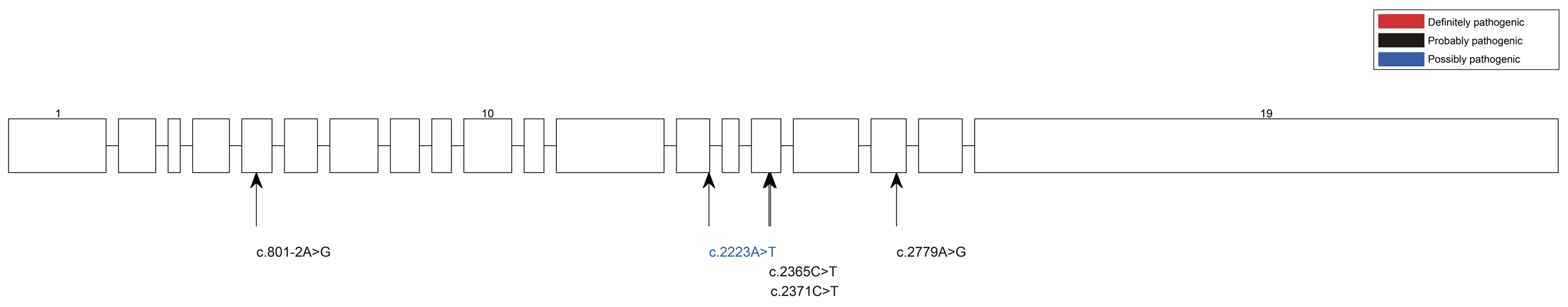


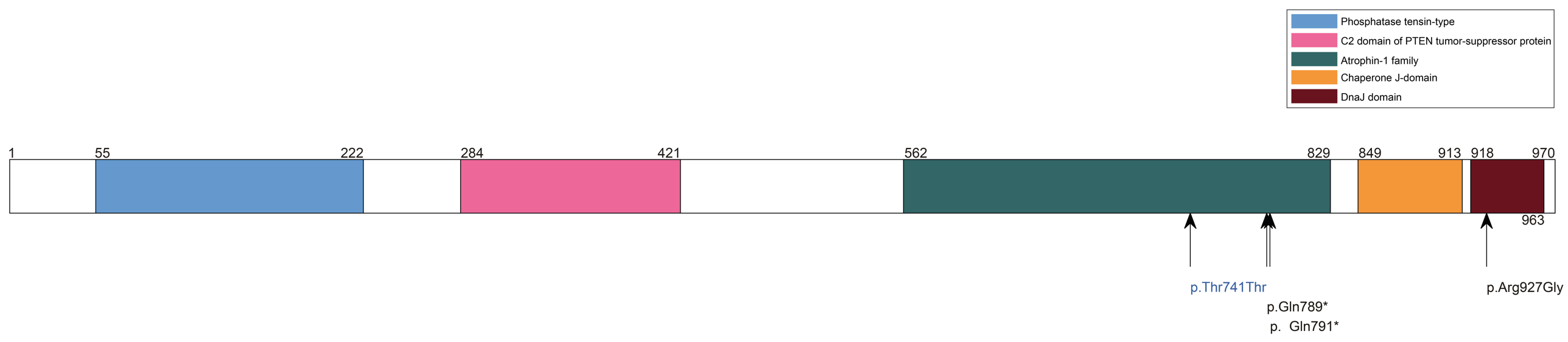


Figure legend. A) Signs and symptoms are listed according to their frequency in reported *DNAJC6* mutation carriers **(limited to signs and symptoms with a frequency of at least 25%)**. B) Splice site mutation is not illustrated because of an unpredictable effect.

**Supplementary Figure 7. A) Signs and symptoms in reported *FBXO7* mutation carriers B) Schematic representation of the *FBXO7* gene (upper scheme) and protein (lower scheme) and mutations listed in MDSGene.**

**A)**

**
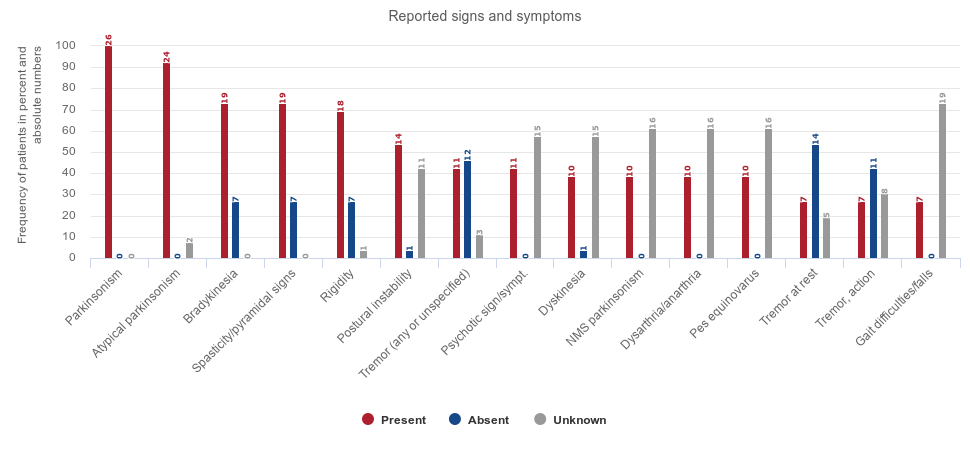
**

**B)**


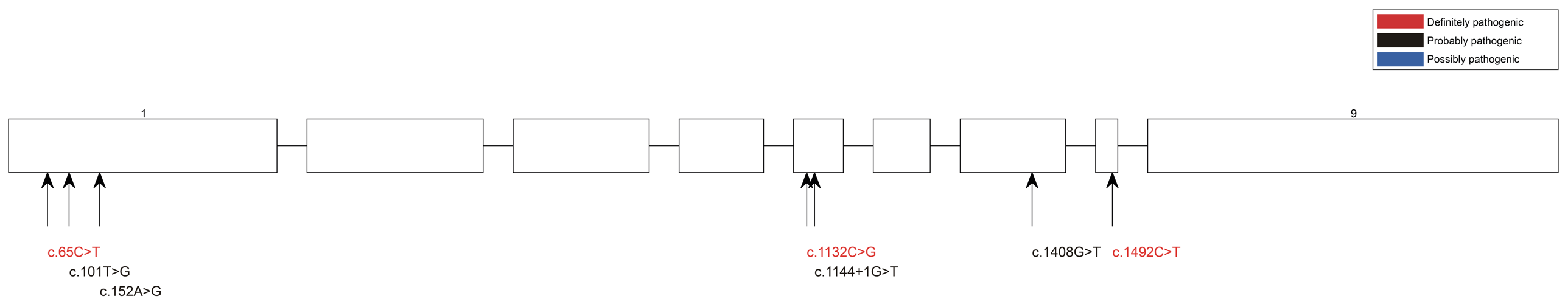


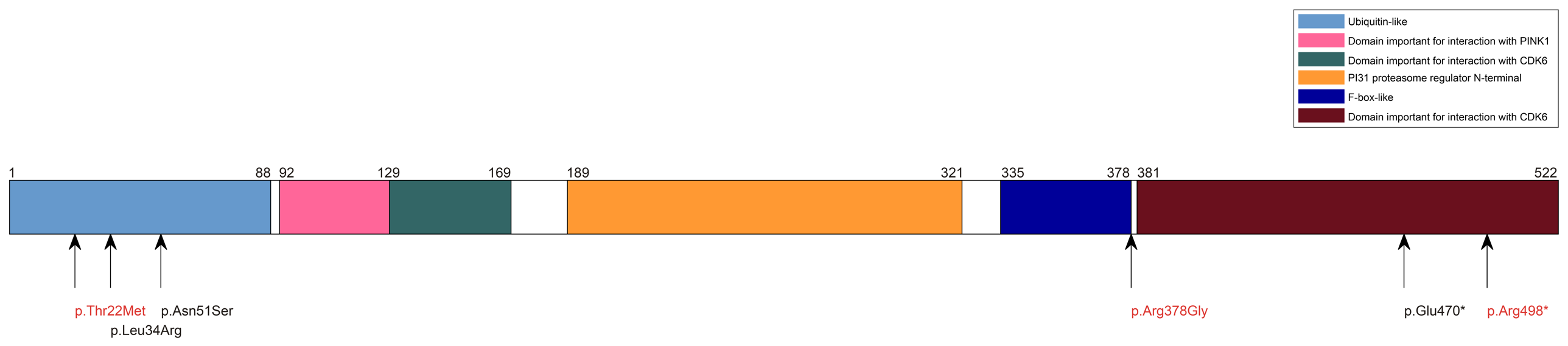


Figure legend. A) Signs and symptoms are listed according to their frequency in reported *FBXO7* mutation carriers **(limited to signs and symptoms with a frequency of at least 25%)**. B) Splice site mutation is not illustrated because of an unpredictable effect.

**Supplementary Figure 8. A) Reported signs and symptoms in PD patients with *SYNJ1* mutations. B)** **Schematic representation of the *SYNJ1* gene (upper scheme) and protein (lower scheme) and mutations listed in MDSGene**

**A)**

**
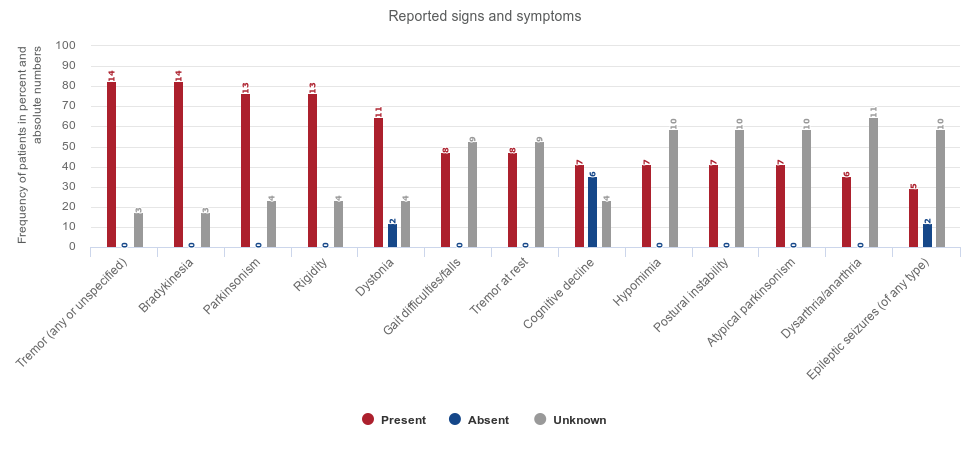
**

**B)**


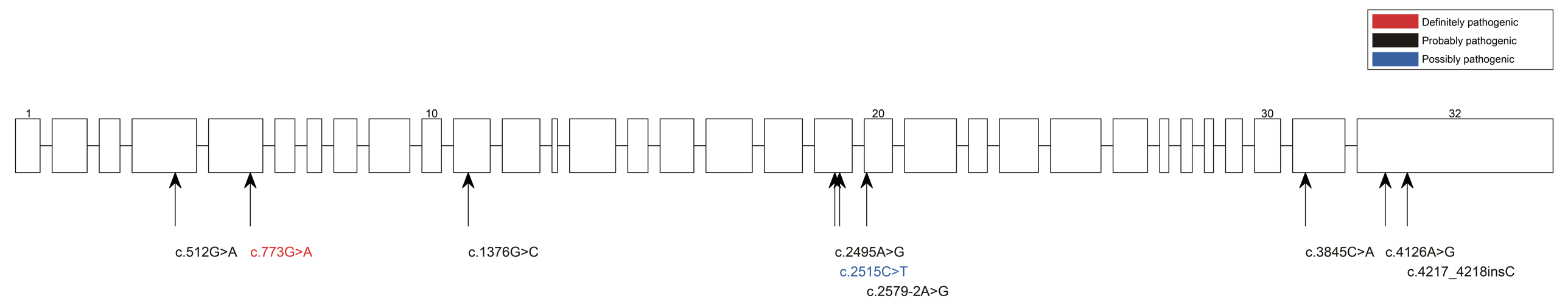

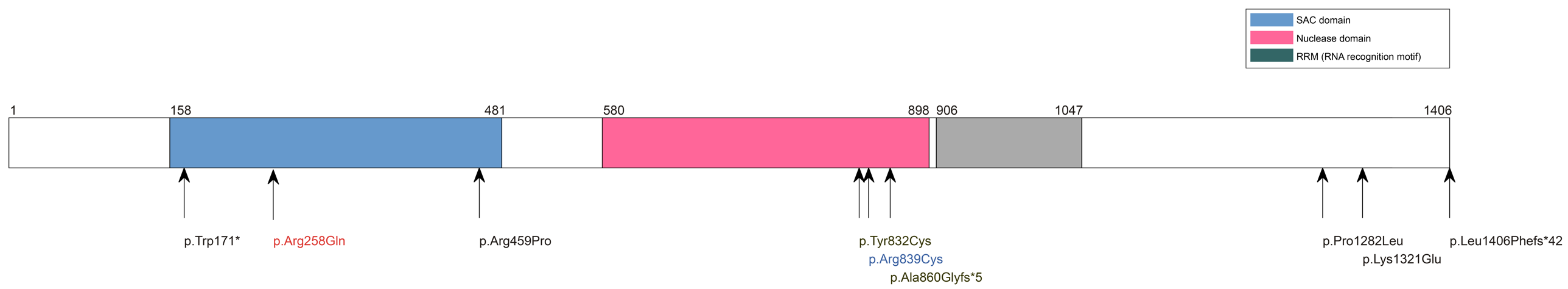


Figure legend. A) Signs and symptoms are listed according to their frequency in reported *SYNJ1* mutation carriers **(limited to signs and symptoms with a frequency of at least 25%)**. B) Splice site mutation results in skipping of exon 20 and a frameshift.**Supplementary Figure 9. A) Signs and symptoms in reported *VPS13C* mutation carriers B) Schematic representation of the *VPS13C* gene (upper scheme) and protein (lower scheme) and mutations listed in MDSGene**

**A)**

**
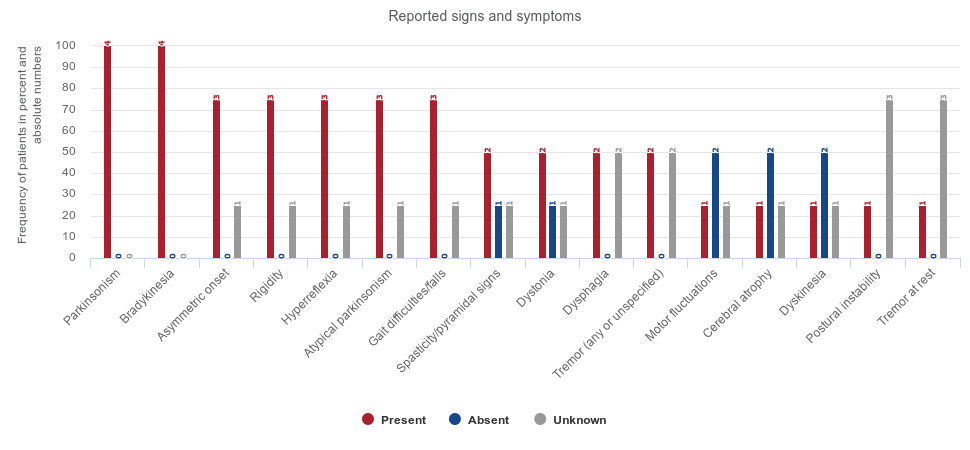
**

**B)**


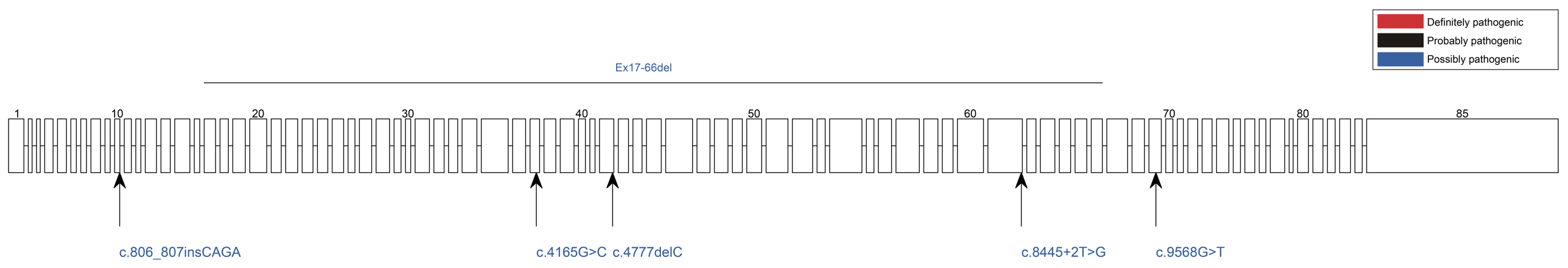


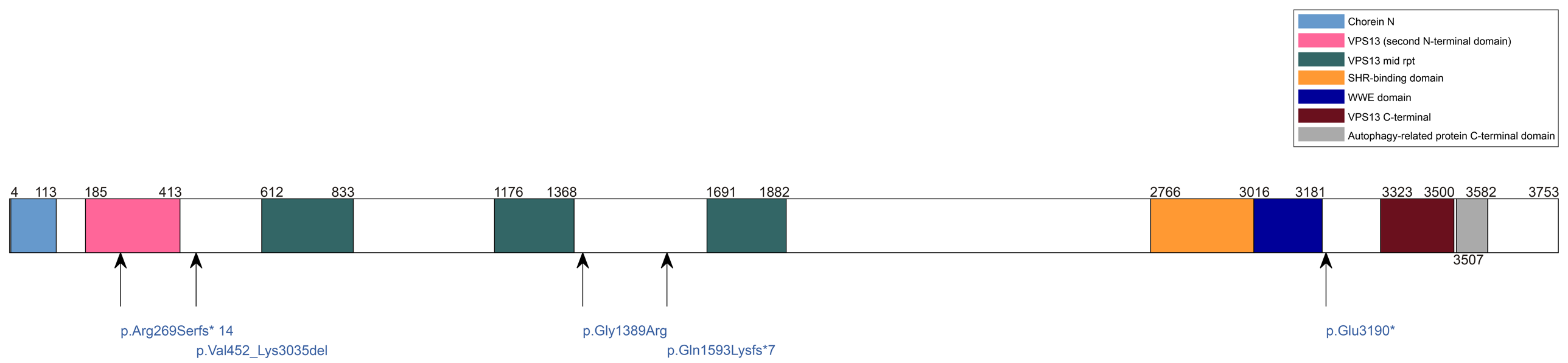


Figure legend. A) Signs and symptoms are listed according to their frequency in reported *VPS13C* mutation carriers **(limited to signs and symptoms with a frequency of at least 25%)**. B) Splice site mutation is not illustrated because of an unpredictable effect.

**Supplementary Figure 10. Classification results across all six genes linked to atypical parkinsonism**


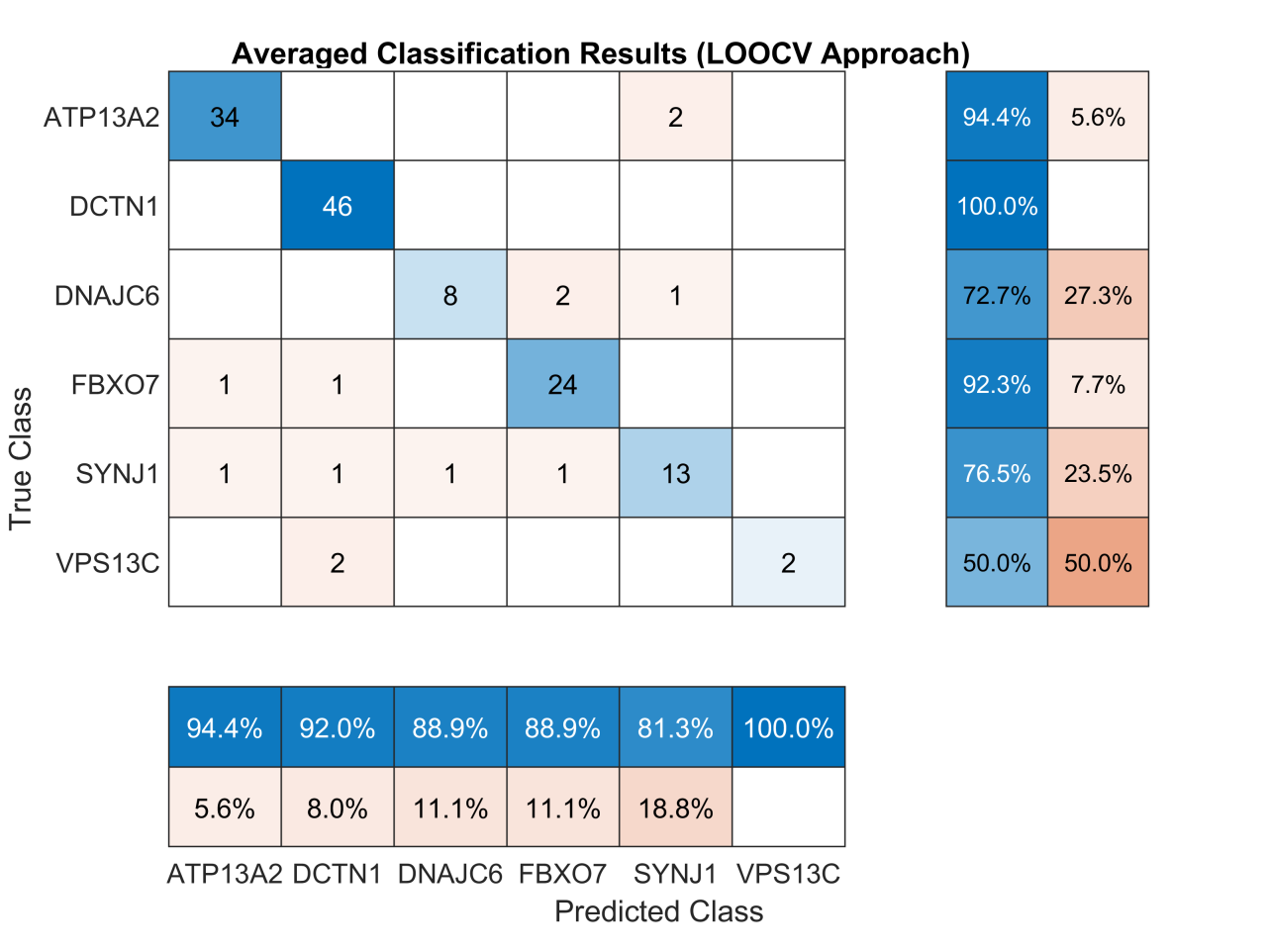


Figure legend. Main panel: Averaged classification results after 10 repetitions of leave-one-out cross-validation (LOOCV), presented as confusion matrix for all six gene groups. Right panel: recall or sensitivity (TPR) and false negative rates (FNR). Bottom panel: Summaries of positive predictive values (PPV; i.e. precision) and false discovery rates (FDR).

**Supplementary Figure 11. Overview of missing data for cardinal signs for monogenic atypical parkinsonism.**


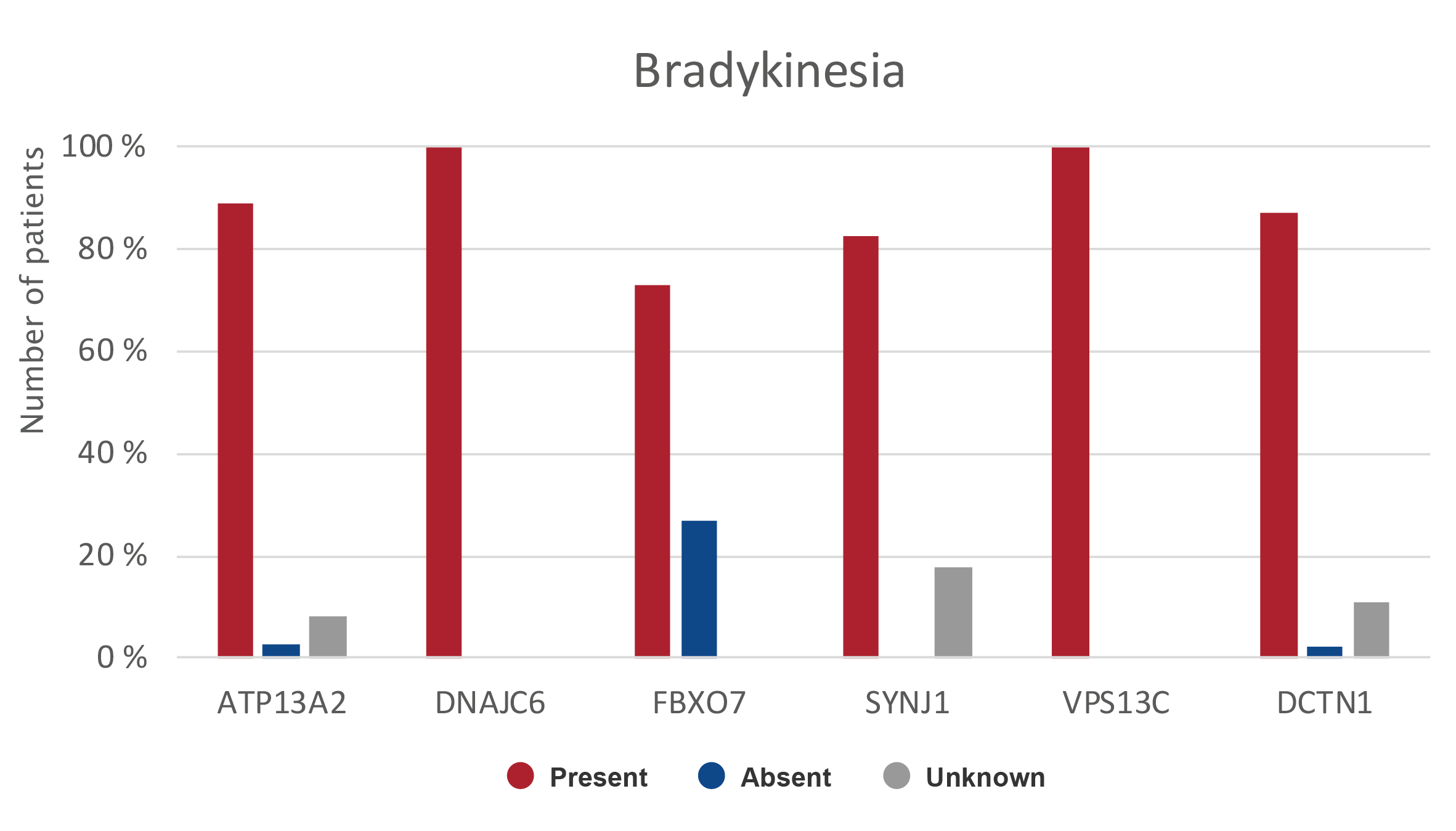


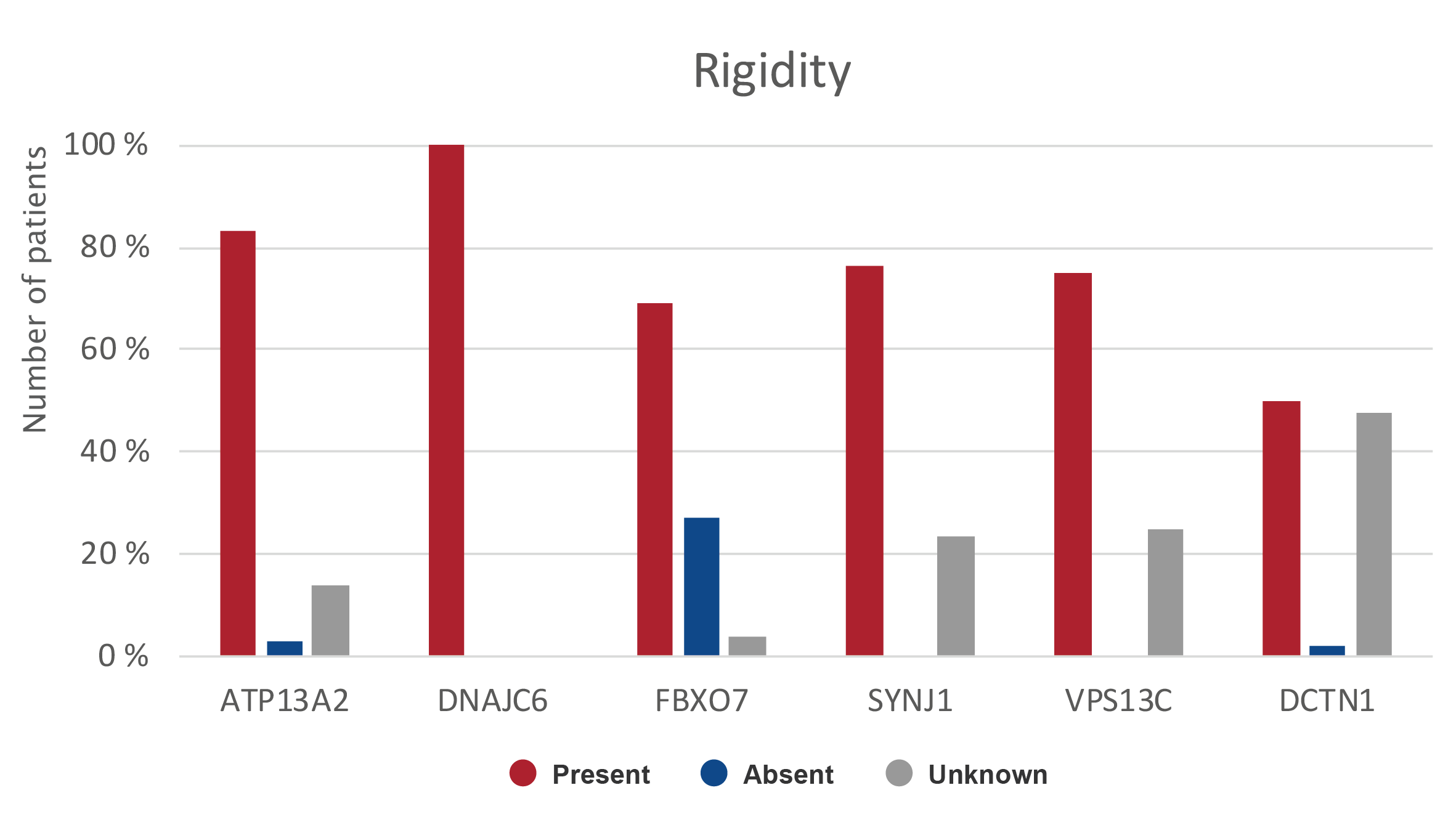


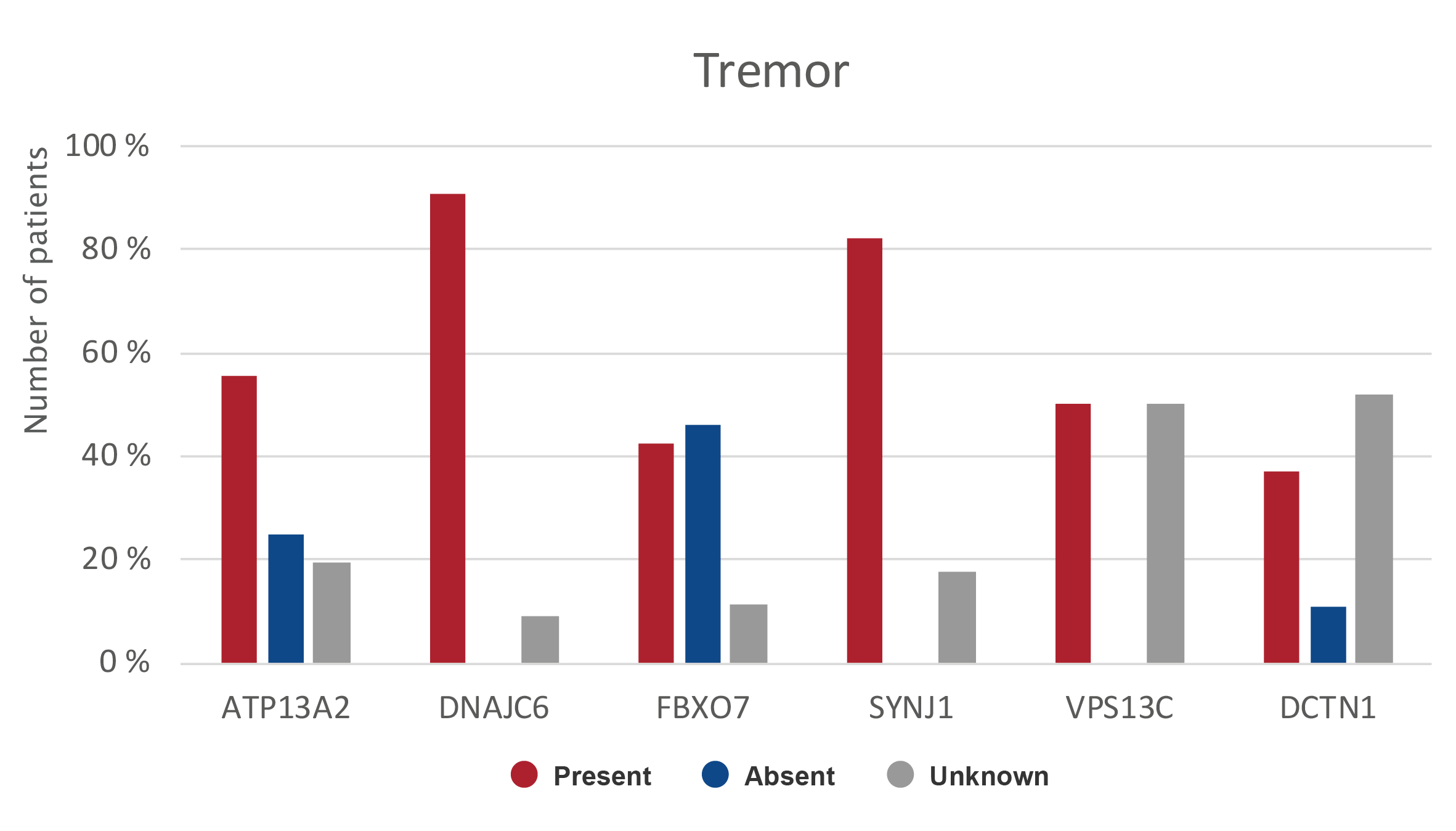

Supplement: Supplementary Material [file NIHMS1796175-supplement-Supplementary_Material.docx]
